# Integrated Self‐Powered Sensors for Continuous Foot Health Monitoring via Laser‐Induced MXene‐Composited Graphene Hybrids From Lignocellulose

**DOI:** 10.1002/advs.202516691

**Published:** 2025-10-22

**Authors:** Peilong Zhao, Xiaofei Mao, Jiashu Song, Man Liu, Luxue Cui, Mingyang Liu, Nan Zhao, Jingqing Gao, Yuguang Zhou

**Affiliations:** ^1^ School of Ecology and Environment Zhengzhou University Zhengzhou 450001 China; ^2^ College of Engineering China Agricultural University Beijing 100083 China

**Keywords:** laser induced graphene, lignocellulose, MXene, TENG, wearable sensor

## Abstract

Intelligent wearable devices based on laser‐induced graphene (LIG) have attracted significant attention for human health monitoring. This paper proposed an innovative all‐in‐one design for preparing a self‐powered smart insole using laser‐induced MXene‐composited graphene hybrid (LIG@MXene) from lignocellulose precursor. By incorporating MXene into the LIG, the composite achieved improved crystallinity and reduced defects, contributing to the electrical conductivity (17.2 Ω∙sq^−1^) and structural stability. The optimal laser processing parameters are 55% for laser power and 70 mm s^−1^ for etching rate. The optimized LIG@MXene composite functions as a versatile platform for integrating triboelectric nanogenerator (TENG) with a high output power of 35 V cm^−2^, supercapacitor with a superior areal capacitance of 71.4 mF cm^−2^ and the excellent cycling stability of 89.5% retention, Joule heater of the maximum heating temperature of 113 °C at 5 V, and various flexible sensors for pressure, humidity and sweat composition with high sensitivity and linearity. In particular, the minimum L‐tyrosine limit of detection in sweat is only 9.60 µM. These functional modules are embedded within an insole via a direct laser writing technology, which only emitted 9.10 kg CO_2_ eq during manufacturing. The direct laser‐patterned synthesis of LIG@MXene composite represents a significant step forward in advancing smart wearable electronic devices.

## Graphical abstract

This study presents a high‐performance foot‐monitoring platform based on lignocellulose‐derived laser‐induced MXenecomposited graphene hybrids (LIG@MXene). The LIG@MXene exhibits markedly improved electrical conductivity, structural stability, and multifunctionality, enabling integrated energy harvesting, sensing, Joule heating, and supercapacitor storage. The material enables continuous monitoring of foot pressure, humidity, and sweat biochemistry, offering strong support for sustainable wearable health technologies.

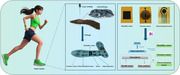

## 1 Introduction

With socioeconomic advancement and higher living standards, the demand for health management has steadily increased. Wearable devices have progressed from basic wrist‐based heart rate monitors to sophisticated multi‐dimensional health sensing systems.^[^ ^1^, ^2^ ^]^ While technologies for tracking respiratory rhythms and muscle activity in the thoracic‐abdominal region and monitoring motion postures at limb joints are advancing, deep monitoring solutions for the feet have emerged as a research hotspot due to their unique biomechanical significance.^[^ ^3^, ^4^ ^]^ As the primary weight‐bearing structures that continuously interact with the ground during movement, dynamic plantar pressure distribution and gait patterns not only reflect the functional status of the musculoskeletal system^[^ ^5^, ^6^ ^]^ but also offer early warning signals for conditions such as diabetic foot ulcers, arthritis, and neurodegenerative diseases through abnormal mechanical cues.^[^ ^7^, ^8^ ^]^ Flexible sensing systems embedded within insoles enable continuous measurement of 3D biomechanical parameters and sweat metabolite concentrations, supporting cross‐analysis models that correlate gait stability, pressure imbalance zones, and metabolic disorder markers.^[^ ^9^, ^10^ ^]^ This provides objective data for the early detection of peripheral neuropathy, muscle atrophy, and metabolic syndrome.^[^ ^11^, ^12^ ^]^ Compared to conventional metal‐based sensors constrained by rigid structures and high costs, flexible sensing technologies utilizing nanomaterials like graphene achieve a synergistic design of stretchable electrodes and biomimetic microstructures.^[^ ^13^, ^14^ ^]^ These innovations deliver highly sensitive pressure detection and precise electrochemical molecular recognition while significantly improving durability and comfort under high‐frequency foot deformation scenarios. By integrating physical signal acquisition with biochemical indicator analysis, smart insoles evolve from simple motion aids into multifunctional health management platforms encompassing chronic disease prevention, rehabilitation evaluation, and fall risk alerts for the elderly.^[^ ^8^ ^]^

In recent years, laser‐induced graphene (LIG) has been widely utilized in flexible sensors due to its notable advantages, including rapid patterning, mask‐free fabrication, in situ synthesis capability, and cost‐effective production.^[^ ^15^, ^16^ ^]^ The 3D porous structure of LIG can provide numerous active sites for chemical reactions and physical interactions.^[^ ^17^, ^18^ ^]^ When interfacing with electrolytes, metabolites, hormones, and other chemical markers, LIG induces changes in electrical potential, current, and resistivity, thereby enabling the detection and analysis of analytes through these variations.^[^ ^19^ ^]^ Importantly, precise control over LIG's surface morphology, internal structure, and porosity can be achieved by adjusting laser processing parameters, offering a simple yet effective way to tailor the microtopography and electrical properties of LIG‐based sensing elements.^[^ ^20^ ^]^ Currently, LIG has been integrated into various sensing devices, including electrochemical sensors^[^ ^21^ ^]^ and pressure sensors.^[^ ^22^ ^]^ However, most LIG precursors are derived from non‐renewable fossil resources, posing conflicts with national sustainable development strategies. As one of the most abundant renewable resources, lignocellulose, composed of lignin, cellulose, and other components tightly interwoven through complex physicochemical interactions, can offer an ideal carbon source for sustainable graphene synthesis.^[^ ^23^, ^24^ ^]^ Its natural hierarchical structure makes it highly suitable for green production processes.^[^ ^25^ ^]^ Most existing research on lignocellulose‐based LIG involves directly converting raw lignocellulose into LIG.^[^ ^26^, ^27^ ^]^ However, due to the heterogeneous distribution of its key components (lignin and cellulose), the resultant LIG often exhibits inconsistent quality, thereby reducing the performance reliability of electronic devices.^[^ ^28^ ^]^

The incorporation of highly conductive materials has emerged as a promising strategy for enhancing the conductivity of LIG. Common post‐processing doping approaches, such as electrodepositing nanoparticles,^[^ ^29^ ^]^ spin‐coating conductive materials onto the LIG surface,^[^ ^30^ ^]^ and performing secondary laser induction of metallic precursor solutions,^[^ ^31^ ^]^ have been widely explored. Recently, MXene has gained significant attention in the sensor field as a highly conductive material. The synergistic integration of LIG and MXene has increasingly been investigated, providing an innovative solution to overcome the limitations associated with using MXene independently. For instance, Su et al.^[^ ^32^ ^]^ developed a high‐performance sensing platform based on femtosecond laser‐induced MXene composite LIG (LIMG), which was fabricated in situ by embedding MXene into polyamide acid precursors. Similarly, Deshmukh et al.^[^ ^33^ ^]^ proposed a micro‐supercapacitor based on picosecond laser‐induced MXene‐functionalized graphene, which achieved interface synergistic enhancement through Ti─O─C covalent bonds. Most currently utilized LIG precursors are petroleum‐based materials, such as polyimide, which contradict sustainability strategy goals. As a viable alternative, lignocellulose combined with MXene for LIG fabrication will not only decrease dependence on petroleum‐derived precursors but also significantly enhance electrical conductivity. Therefore, further investigation into the formation mechanisms of LIG from lignocellulose/MXene composites under laser induction and its resulting material properties holds substantial research value.

This paper employed direct laser writing to fabricate LIG@MXene composite functional materials on lignocellulose/MXene composite films using blue laser etching. Systematic optimization of laser parameters was performed to regulate the material compounding process, which significantly enhanced the electrical conductivity and structural properties of the composite. The triboelectric nanogenerator (TENG) developed from this composite system delivered a high output performance, and the assembled supercapacitor also exhibited exceptional energy storage capacity and cycling stability.^[^ ^34^, ^35^, ^36^ ^]^ Additionally, the joule heater demonstrated rapid thermal response capabilities, the pressure sensor achieved precise and linear signal output, and the electrochemical sensor offered low detection limits for biomolecular analysis. This study presented an innovative technical solution for smart, flexible, and wearable systems that integrated power generation, energy storage, and sensing functionalities in an insole. Its chemical additive‐free and green fabrication process can provide valuable insights for the sustainable development and industrial applications of foot health monitoring.

## 2 Results and Discussion

### 2.1 Laser‐Induced Graphene

The quality of LIG was closely linked to laser parameters such as power, etching rate, and focal length.^[^ ^37^ ^]^ Figure S2 (Supporting Information) presents the SEM images of lignocellulose and LIG, illustrating the structural transformations induced by the laser etching process. As shown in Figure S2a,b (Supporting Information), lignin was uniformly distributed across cellulose surfaces, forming an interconnected lignocellulosic architecture stabilized by robust intermolecular interactions. The morphology of post‐etching LIG, illustrated in Figure S2c,d (Supporting Information), revealed that the liberation of volatile gases during laser irradiation produced a porous and foam‐like structure,^[^ ^38^ ^]^ contributing to the LIG's exceptionally high specific surface area. This study then prioritized optimizing laser power, with the results detailed in Figure S3a (Supporting Information). As shown in the data, under a constant laser etching rate of 70 mm∙s^−1^, lignocellulose‐based LIG fabricated at 35% laser power exhibited a significantly higher sheet resistance of 108 Ω∙sq^−1^. When the laser power increased to 55%, the sheet resistance was reduced markedly to 25.1 Ω∙sq^−1^. This change was attributed to the insufficient carbonization and graphitization of lignocellulose at lower laser energy densities, while higher power could facilitate a more complete structural transformation into graphene. However, the laser power more than a certain range also had an impediment on the LIG quality. When the laser power was 75%, the sheet resistance rose progressively to 55.2 Ω∙sq^−1^. These results indicate that both excessive and insufficient laser energy densities compromise the formation of high‐quality LIG. Furthermore, when the laser etching rate was 100 mm∙s^−1^, the sheet resistance of LIG at 55% laser power was measured as 106 Ω∙sq^−1^. A gradual decrease in the etching rate resulted in a corresponding reduction in sheet resistance. Specifically, when the etching rate was 70 mm∙s^−1^, the sheet resistance of LIG was lowest. However, further reducing the etching rate also proved detrimental. When the etching rate decreased to 40 mm∙s^−1^, the sheet resistance of LIG increased to 35.5 Ω∙sq^−1^ because of the prolonged exposure of the laser beam on a single position of the lignocellulosic fiber, which would lead to the over‐etching. Such over‐etching disrupted the material's structural integrity, which was deemed harmful to the formation of high‐quality LIG.^[^ ^39^ ^]^

Figure S3b (Supporting Information) presents the Raman spectra of LIG with the varying laser power under a constant laser etching rate of 70 mm∙s^−1^, where no distinct characteristic peaks were observed in the Raman profile of lignocellulose. After laser etching, the Raman spectra of the resulting LIG consistently showed three prominent peaks: D (1350 cm^−1^), G (1580 cm^−1^), and 2D (2680 cm^−1^).^[^ ^40^ ^]^ Figure S3c (Supporting Information) displays the I_D_/I_G_, I_G_/I_2D_, and L_a_ values for lignocellulose‐based LIG under different laser intensities. The data reveal that as the laser power increased from 35% to 55%, the I_D_/I_G_ ratio decreased from 2.56 to 1.26, I_G_/I_2D_ decreased from 24.3 to 1.15, and L_a_ increased from 7.49 to 15.2. These changes indicated the reduced lattice defects, fewer graphene layers, and increased crystallite size when lignocellulose was transferred into LIG. However, further increases in laser power would lead to a gradual rise in both I_D_/I_G_ and I_G_/I_2D_ ratios, accompanied by a reduction in L_a_ value, suggesting the development of enhanced lattice defects, decreased graphene layers, and diminished crystallite size. These results also confirmed that both insufficient and excessive laser power were detrimental to achieving high‐quality lignocellulose‐based LIG.^[^ ^41^ ^]^

The Raman spectral data curves of lignocellulose‐based LIG under different laser etching rates at 55% laser power are shown in Figure S3d (Supporting Information), where distinct D, G, and 2D peaks were observed across all samples. At a laser etching rate of 100 mm∙s^−1^, the I_D_/I_G_, I_G_/I_2D_, and L_a_ values for lignocellulose‐based LIG were recorded as 2.86, 11.4, and 6.72, respectively. Reducing the etching rate to 70 mm∙s^−1^ minimized the I_D_/I_G_ and I_G_/I_2D_ ratios to 1.26 and 1.15, respectively, while increasing L_a_ value to 15.2, which indicated the reduced lattice defects, fewer graphene layers, and an increase in crystallite size. However, further decreasing the etching rate would result in the elevated I_D_/I_G_ and I_G_/I_2D_ ratios, accompanied by a decline in L_a_ value, signifying a deterioration in graphene quality (Figure S3e, Supporting Information).

Figure S3f,h (Supporting Information) display the XPS spectra of lignocellulose‐based LIG under varying laser intensities and etching rates, respectively. In all lignocellulose‐based LIG samples, the distinct C, O, and P elements were detected, with their relative concentrations influenced by laser parameters. While lignocellulose primarily consisted of C and O elements, the presence of P was attributed to the application of a nitrogen‐phosphorus flame retardant during pre‐etching sandblasting. Figure S3g (Supporting Information) summarizes the elemental composition under different laser power levels. Before laser etching, lignocellulose was dominated by O, which then combined with H to form water vapor and was released during laser etching, leaving C as the predominant element. As laser power increased incrementally from 35% to 55%, the content of C progressively rose, peaking at 86.7% at 55% laser power. However, the further increase in laser power caused a decrease in C content, dropping to 73.7% at 75% intensity. Figure S3i (Supporting Information) presents the elemental composition data for LIG under varying laser etching rates. The findings demonstrated that both excessively low and high etching rates would lead to reduced C content. The highest C concentration (86.7%) was achieved at an intermediate etching rate of 70 mm∙s^−1^ at 55% laser power.

Figure S4 (Supporting Information) presents the C1s fitting data of LIG under varying laser processing parameters. In Figure S4a (Supporting Information), the C1s profile of raw lignocellulose was characterized by predominantly sp^3^‐hybridized C─C bonds, along with substantial C─O(H) and C─O─C bonds, and a high oxygen content. After laser etching, a gradual transition from sp^3^ to sp^2^ hybridized C─C bonds was observed. The highest sp^2^‐hybridized C‐C content was achieved at 55% laser power when the laser etching was 70 mm∙s^−1^ (Figure 2l). However, the further increase in laser power resulted in a decline in sp^2^‐hybridized C─C bonds, demonstrating that both excessively high (Figure S4d,e, Supporting Information) and low (Figure S4b,c, Supporting Information) laser power were unfavorable for graphene formation. These results aligned with earlier Raman spectroscopy and sheet resistance measurements. Similarly, the reduced sp^2^‐hybridized C‐C content was also detected when the laser etching rate was either too low (Figure S4f,g, Supporting Information) or too high (Figure S4h,i, Supporting Information).

### 2.2 Laser‐Induced MXene‐Composited Graphene

**Figure 1:**
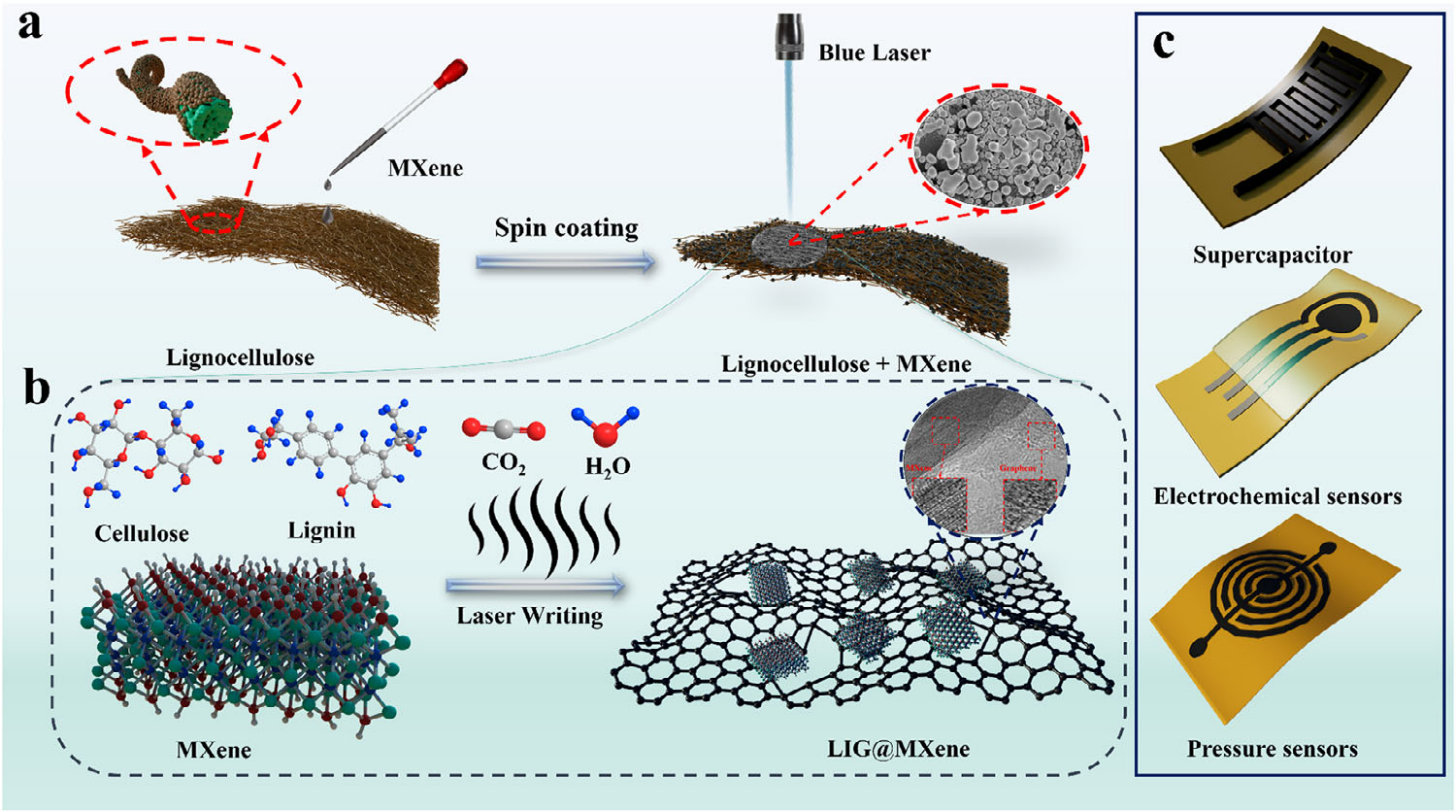
a) Schematic diagram of the synthesis of LIG@MXene using laser etching, b) mechanism diagram of LIG@MXene fabricated by laser etching, c) LIG@MXene for manufacturing supercapacitors, biosensors, and pressure sensors.

SEM images of MXene prior to delamination are shown in Figure S5a,b (Supporting Information), exhibiting an accordion‐like multilayer stacked structure. After delamination using DMSO, MXene adopted a 2D and sheet‐like morphology (Figure S5c,d, Supporting Information). This 2D architecture integrated seamlessly with LIG, enhancing the electrical conductivity of LIG and stabilizing the performance of the resulting LIG@MXene composite.^[^ ^42^, ^43^ ^]^ Following MXene delamination, a 5 wt.% dispersion was prepared by mixing delaminated MXene with DI water. This dispersion was then uniformly spin‐coated onto the lignocellulose substrate at 500 rpm for 60 s. Post‐coating, the MXene‐coated lignocellulose was irradiated with a blue laser for patterning, as depicted in **Figure** **1**a. The binding mechanism between LIG and MXene is illustrated in Figure 1b. When the laser focused on the lignocellulose/MXene composite, field‐induced ionization (via multiphoton and tunnel ionization) generated excited electrons. These electrons transitioned from bonding to antibonding states, weakening the electronic bonds near C atoms at the valence band maximum and directly triggering ionization of oxygen and carbon atoms.^[^ ^44^ ^]^ Upon laser etching, a substantial population of free electrons was generated, rupturing C─O─C and C─O(H) bonds in lignocellulose as well as Ti bonds in MXene.^[^ ^39^ ^]^ Subsequently, the electron‐hole recombination occurred at the LIG/MXene interfaces, promoting the formation of the novel LIG@MXene composite.^[^ ^32^ ^]^ During this process, some C, O, and H atoms were liberated as CO_2_ and H_2_O gases, leading to the creation of a 3D porous architecture.^[^ ^45^ ^]^ Residual free C atoms reorganized into graphene lattice frameworks, while reconstituted MXene particles integrated into the graphene structure, yielding a composite that synergistically incorporated both LIG and MXene. The resulting composite retained MXene's high sensitivity to biomolecules and LIG's conductivity, porosity, flexibility, and biocompatibility,^[^ ^46^ ^]^ making it highly suitable for high‐performance sensors and detection systems in wearable electronics. Wearable devices fabricated from LIG@MXene in this study, including supercapacitors, biosensors, and pressure sensors, are illustrated in Figure 1c.

**Figure 2:**
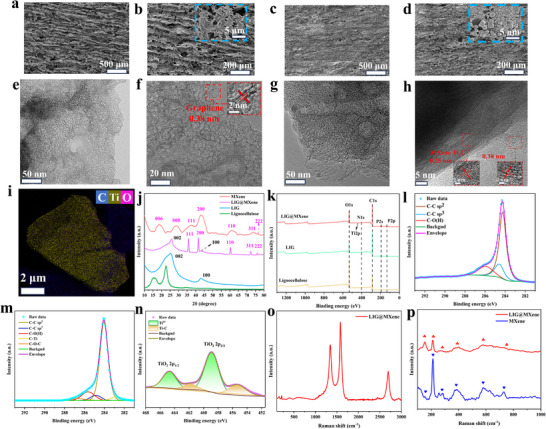
a) SEM and b) local magnification images of lignocellulose‐based LIG, c) SEM and d) partial magnification images of LIG@MXene composite, e) and f) TEM images of lignocellulose‐based LIG, g) and h) TEM images of LIG@MXene composite, i) the elemental mapping image of the layer structure for LIG@MXene with the distribution of carbon (blue), titanium (yellow), and oxygen (purple), j) XRD pattern, k) XPS energy spectrum and l) C1s energy spectrum of lignocellulose‐based LIG, m) C1s spectrum and n) Ti2p spectrum of LIG@MXene composite, Raman spectra of o) LIG@MXene, p) Raman spectra of LIG@MXene and MXene at low wavenumber.

**Figure** **2**a,b presents the SEM images of LIG formed under 55% laser power, showing a high specific surface area and distinct 3D porosity. SEM images of LIG@MXene prepared using the same laser power and etching rate (Figure 2c,d) revealed that the numerous nanoparticles were distributed across its surface (Figure 2d), indicating the successful integration of MXene into the lignocellulose‐based LIG. The sheet resistance of LIG@MXene was measured to be 17.2 Ω sq^−1^, significantly lower than that of pristine lignocellulose‐based LIG (25.1 Ω sq^−1^). Both LIG and LIG@MXene exhibited randomly stacked network architecture in their porous nano‐sheets, attributed to the rapid gas release during thermal decomposition and carbonization caused by the cleavage of C─O(H) and C─O─C bonds.^[^ ^26^ ^]^ TEM images of LIG (Figure 2e,f) demonstrated the extensive grid‐like structures across the laser‐etched lignocellulose surface, with a lattice spacing of 0.38 nm corresponding to the (002) crystallographic plane of graphene. TEM images of LIG@MXene (Figure 2g,h) further depicted the interconnected graphene nanoparticles and dispersed MXene nanoparticles. In Figure 2h, the multiple lattice fringes at varying distances were observed, including a lattice spacing of 0.38 nm assigned to the (002) crystallographic plane of graphene and a 0.25 nm spacing corresponding to the (004) plane of MXene.^[^ ^47^ ^]^ Elemental mapping images of LIG@MXene (Figure 2i) illustrated the distribution of carbon and titanium within the fabricated layered structures.

XRD analysis provided the additional structural insights (Figure 2j). The strongest peak at 45.1°, attributed to the (100) plane reflection, highlighted the highly crystalline nature of graphene within LIG@MXene. Meanwhile, an asymmetric (002) peak between 20° and 30° suggested the presence of amorphous carbon structures, while other characteristic peaks aligned closely with those of pristine MXene. XPS analysis further confirmed the chemical states and composition of LIG@MXene, revealing the presence of Ti, C, O, and P (Figure 2k). The fitted C1s spectrum for LIG (Figure 2l) demonstrated that most carbon exists in the sp^2^ hybrid configuration, indicating substantial graphitic carbon content. For the LIG@MXene composite, the deconvoluted C1s spectrum (Figure 2m) revealed the contributions from C─Ti, C─C, C─O(H), and C─O─C bonds, while the Ti2p spectrum (Figure 2n) validated the presence of C─Ti bonds, confirming the successful incorporation of MXene into LIG, consistent with the results of previous literature studies.^[^ ^32^, ^33^ ^]^ Raman spectroscopy provided further structural characterization of LIG@MXene (Figure 2o,p). Two prominent peaks at 1351 and 1588 cm^−1^ correspond to the D and G bands, respectively, reflecting the carbon‐rich composition. The observed 2D peak at 2660 cm^−1^ confirmed the formation of multilayer graphene.^[^ ^48^ ^]^ The I_D_/I_G_ intensity ratio was measured to be 0.89, further corroborating the multilayer character of the graphene structure. This suggested that the LIG substrate in the LIG@MXene composite had a low graphene lattice defect density and high crystallinity, inherently contributing to the improved conductivity. Meanwhile, the TEM observations showed the densely packed nanoparticles, primarily originating from the stacking morphology of the MXene component itself. More importantly, these MXene‐derived particles formed a strong conductive interface with the LIG substrate. This was the synergistic effect between the high‐quality LIG substrate and functional MXene‐derived particles that led to a significant enhancement in the overall conductivity of the composite material, consistent with the previous research findings.^[^ ^33^ ^]^ Additionally, the low‐wavenumber Raman shifts observed in Figure 2p aligned with MXene‐specific peaks, which also demonstrated the successful incorporation of MXene into the graphene matrix.

### 2.3 TENG

**Figure 3:**
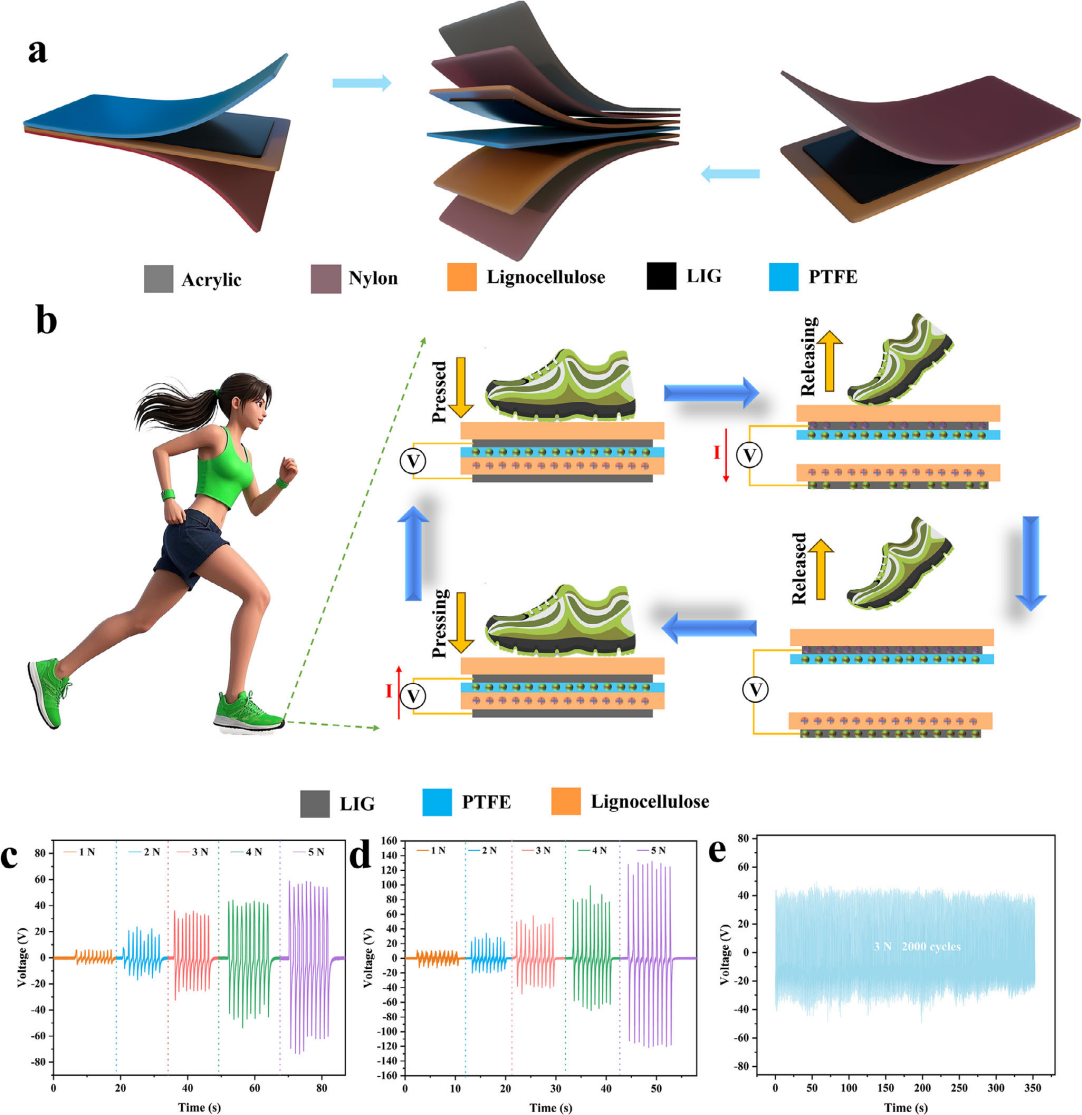
a) Schematic diagram of TENG assembly, b) working mechanism diagram of TENG, the power generation effect of TENG based on c) LIG and d) LIG@MXene composite under different pressures, e) the power generation effect of TENG based on LIG@MXene composite after 10 000 cycles under 3 N action.

The development of TENG based on LIG@MXene composite was reported in this study. The original LIG@MXene layer functioned as the positive electrode, interacting synergistically with the residual lignocellulose layer to construct a collaborative triboelectric system. To enhance the structural durability, double‐layer nylon membranes encapsulated the electrode terminals, while an acrylic plate was incorporated as a load‐bearing layer (**Figure** **3**a). This fabrication approach highlighted the advantages of LIG technology in enabling rapid and precise processing of complex electrode architectures, showcasing application potential in environmental energy harvesting and smart wearable electronics. The working mechanism of the TENG (Figure 3b) operates based on a contact‐separation mode driven by the triboelectric effect. First, charge generation occurred at the PTFE‐lignocellulose interface during contact due to the difference in their triboelectric properties.^[^ ^49^, ^50^ ^]^ Second, electrostatic induction caused reverse charge accumulation in the LIG@MXene electrodes during separation, which triggered current flow through the external circuit. Upon subsequent contact, the charge attraction restored dynamic equilibrium.^[^ ^51^, ^52^ ^]^ As shown in Figure 3c, the open‐circuit voltage of the LIG‐based TENG significantly increased from 1.33 to 12.5 V cm^−2^ as the applied pressure rose from 1 to 5 N. With the incorporation of MXene into LIG, a significant enhancement in voltage output was observed. The maximum output voltage could reach 35 V cm^−2^, demonstrating a 158% increase compared to the pure LIG system (Figure 3d). Meanwhile, this study also tested the output current of the TENG under different pressure ranges (Figure S6, Supporting Information). When the applied pressure increased from 1 to 5 N, the maximum output current of the LIG‐based TENG increased from 0.49 to 5.45 µA (Figure S6a, Supporting Information). After incorporating MXene, the maximum output current of the TENG increased from 0.84 to 12.5 µA (Figure S6b, Supporting Information). This improvement primarily arose from MXene's 2D lamellar structure and high dielectric constant, which provided abundant charge trapping sites and amplified interfacial polarization effects.^[^ ^53^ ^]^ The surface of MXenes was rich in ‐F groups, which would endow them with superior triboelectric properties compared to conventional materials like PTFE. When coupled with LIG, this surface property of MXene enhanced the composite's tribonegative characteristics, leading to an improvement in the power generation performance.^[^ ^54^ ^]^ Additionally, the oxygenated surface functional groups (─O, ─OH) also promoted the electron transfer through dipole–dipole interactions with PTFE, as supported by recent studies on 2D material‐enhanced triboelectric systems.^[^ ^55^ ^]^ The effective power density (P) of the LIG@MXene‐based TENG was calculated using the equation *P = U^2^/(RA)* (U is the output voltage, R is the load resistance, and A is the effective area of the TENG). The output power density of the TENG was evaluated with varying external load resistances ranging from 100 Ω to 1000 MΩ. As shown in Figure S7 (Supporting Information), the output voltage increased with the external load resistance, whereas the current decreased according to Ohm's law. The instantaneous power density initially increased with the load resistance, reaching a maximum of ≈97.1 mW∙m^−2^ at 9 MΩ, and then decreased. To demonstrate the practical applicability of the developed TENG, the charging capability was tested by connecting it to a capacitor through a full‐wave bridge rectifier (Figure S8a, Supporting Information). As shown in Figure S8b (Supporting Information), the capacitor (25.0 µF) could be charged to ≈17.8 V within 70 s. When compared with existing TENG research (Table S4, Supporting Information), this enhancement in power generation performance was particularly notable. Durability tests validated the structural reliability of the device, showing an 84.3% output retention after 10 000 cycles under 3 N pressure (Figure 3e). This high retention rate could be attributed to the MXene's intrinsic mechanical stability, which prevented the structural degradation during the repeated contact‐separation cycles.^[^ ^30^ ^]^

### 2.4 Supercapacitor

**Figure 4:**
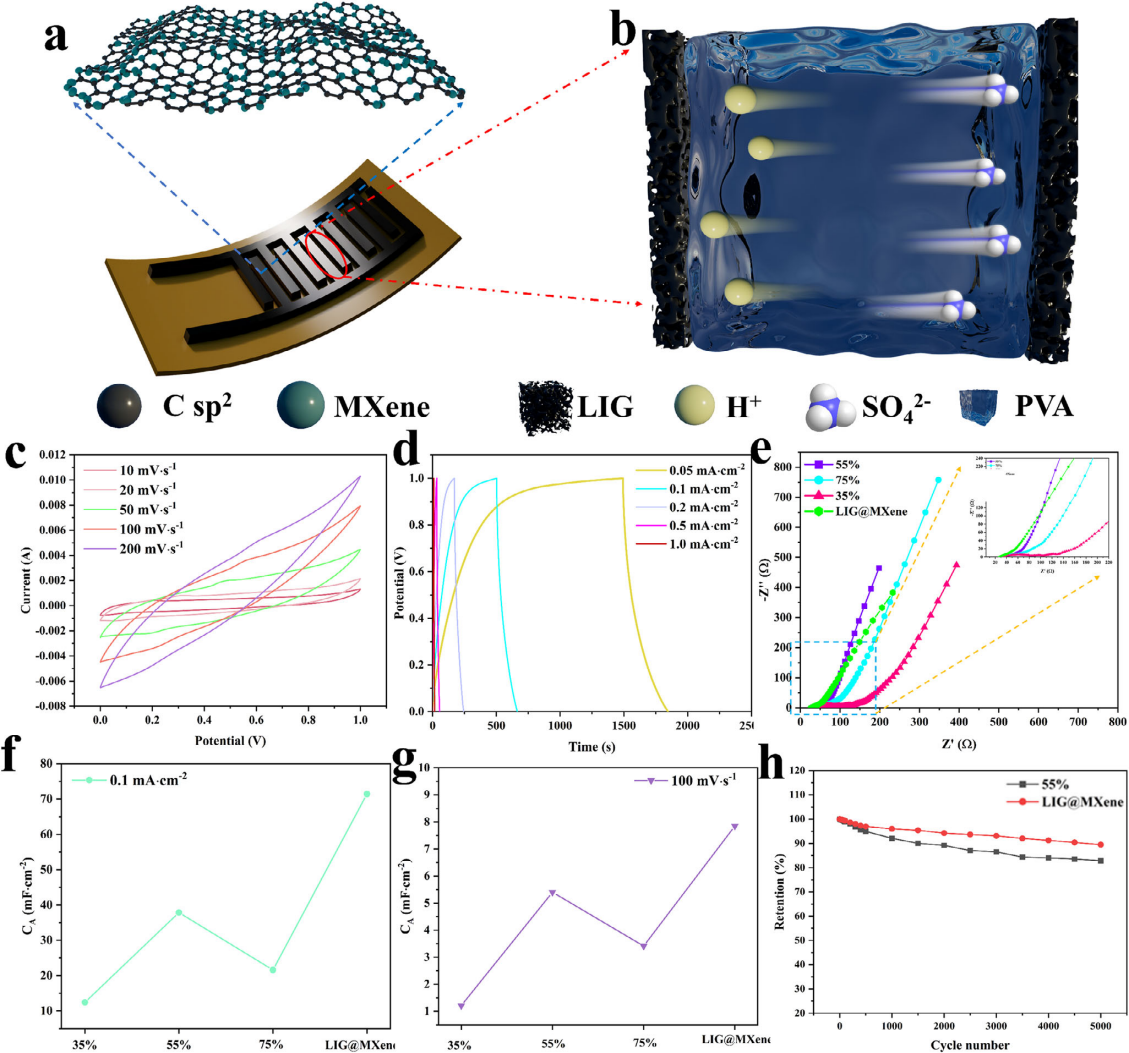
a) Macroscopic structure of supercapacitors and microscopic graphene grids, b) ion migration mechanism of supercapacitor electrolyte, c) CV curves and d) GCD curves of supercapacitor based on LIG@MXene composite at different scanning rates and current densities (55% laser power and 70 mm∙s^−1^), e) EIS of supercapacitors, f) C_A_ of the supercapacitor at a current density of 0.1 mA∙cm^−2^, g) C_A_ of the supercapacitor at a scanning rate of 100 mV∙s^−1^, h) C_A_ retention rate of the supercapacitor after 5000 charge–discharge cycles at a current density of 0.1 mA∙cm^−2^.

As shown in **Figure** **4**a, the interdigital‐shaped electrodes were fabricated in this study for supercapacitors. After coating the LIG with an H_2_SO_4_/PVA solid electrolyte, H^+^ and SO_4_^2−^ ions migrated in opposite directions between the electrodes (Figure 4b), effectively storing charge within the LIG matrix. Supercapacitors based on pure LIG were fabricated by etching lignocellulose at varying laser power levels (35%, 55%, and 75%), followed by their electrochemical characterization. LIG electrodes produced at all three laser power levels exhibited quasi‐rectangular CV curves and nearly triangular GCD curves (Figures S6–S8, Supporting Information), indicative of an electric double‐layer capacitor (EDLC) charge storage mechanism.^[^ ^56^ ^]^ Representative electrochemical performance data for supercapacitors fabricated at 35% and 75% laser power are presented in Figure S9 (Supporting Information). Specifically, Figure S9a,e (Supporting Information) illustrated the CV curves obtained at different scan rates, demonstrating the pseudo‐rectangular shapes for the supercapacitors at 35% and 75% laser power, respectively. Concurrently, GCD curves displayed in Figure S9b,f (Supporting Information) exhibited the symmetrical triangular profiles, corroborating favorable electrochemical behavior. However, the C_A_ values of supercapacitors prepared at 35% and 75% laser power were lower than those observed for the sample produced at 55% laser power. This was attributed to the incomplete conversion of lignocellulose to LIG at lower laser power (35%), which increased the concentration of defects within the LIG structure. These defects elevated surface resistance, thereby diminishing energy storage performance. Conversely, higher laser power (75%) also disrupted the aromatic ring structures essential for LIG formation due to excessive energy input, leading to the generation of amorphous carbon and a subsequent reduction in energy storage capacity. Figure S9c,d (Supporting Information) depicted the C_A_ variations derived from CV and GCD curves for the supercapacitor based on LIG at 35% laser power, showing a decrease in C_A_ with the increasing scan rates and current densities. For the supercapacitor at 75% laser power, C_A_ also decreased with the increasing scan rates and current densities (Figure S9g,h, Supporting Information). The supercapacitors exhibited the C_A_ retention of only 63.1% (35%) and 75.6% (75%) after 5000 cycles at a current density of 0.1 mA cm^−2^ (Figure S9i, Supporting Information). Supercapacitors were also fabricated at three different scanning rates (40, 70, and 100 mm s^−1^) to evaluate their electrochemical performance (Figures S10 and S11, Supporting Information). Excessively fast laser etching rates would result in insufficient dwell time on lignocellulose, leading to incomplete conversion and the formation of LIG with significant defects and high non‐carbon heteroatom content (e.g., oxygen), which then adversely impacted the supercapacitors performances. On the other hand, excessively slow etching rates also prolonged laser irradiation at localized sites, disrupting the aromatic ring structures of lignocellulose and producing more amorphous carbon, consequently degrading LIG quality and reducing energy storage performance. The supercapacitors fabricated at 40 and 100 mm s^−1^ exhibited the C_A_ retention of only 76.5% and 72.9% after 5000 cycles at a current density of 0.1 mA cm^−2^, respectively.

With the introduction of MXene, the macropores on the LIG@MXene surface facilitated the electrolyte diffusion, while micropores increased the electrochemical active surface area (ESA).^[^ ^57^ ^]^ The incorporation of MXene enhanced the porosity and roughness of LIG@MXene composite, improving the electrolyte ion accessibility within the porous structure.^[^ ^33^ ^]^ This unique composite architecture, achieved through the uniform integration of nano‐scale MXene onto porous LIG, provided both substantial specific surface area and abundant functional groups. These features demonstrated the potential of the LIG@MXene composite as an electrode material for supercapacitors. Figure 4c,d illustrates the CV and GCD data obtained during the testing of supercapacitors based on LIG@MXene at 55% laser power and 70 mm s^−1^, respectively. For pure LIG (Figure S11a, Supporting Information), the CV curves exhibited similar pseudo‐rectangular shapes across varying scan rates, while the GCD curves also displayed similar symmetrical triangular profiles at different current densities (Figure S11b, Supporting Information). The C_A_ values of both supercapacitors based on pure LIG and LIG@MXene decreased with the scan rates and current densities increased (Figure S12, Supporting Information). The impedance data of the supercapacitor were fitted using the commercial software ZView, with the equivalent circuit model (Figure S13, Supporting Information). The selected model was based on a simplified Randles circuit, consisting of a series resistance and a parallel branch. In this configuration, the ESR represented the uncompensated solution resistance, while the parallel branch comprised the double‐layer capacitance and an impedance element. To account for the non‐uniformity of the electrode surface, the double‐layer capacitance was replaced by a constant phase element (CPE1), providing a more accurate description of the electrochemical behavior of the system. As the laser power increased from 35% to 55%, the equivalent series resistance (ESR) of the supercapacitor based on pure LIG decreased from 82.7 to 34.9 Ω, and then rose again to 54.1 Ω when the laser power increased to 75%. This trend was similar to the surface resistance of pure. After the incorporation of MXene, the ESR value significantly decreased to only 19.3 Ω (Figure 4e). The C_A_ value also increased from 37.9 to 71.4 mF cm^−2^ at a current density of 0.1 mA cm^−2^ (Figure 4f), surpassing benchmarks reported in existing research (Table S5, Supporting Information). Meanwhile, the C_A_ value increased from 5.40 to 7.85 mF cm^−2^ at a scan rate of 100 mV s^−1^ (Figure 4g). Beyond boosting capacitance, MXene also extended the cycling life of supercapacitors. After incorporating Mexene into LIG, the C_A_ retention after 5000 charge–discharge cycles at a current density of 0.1 mA cm^−2^ increased from 82.9% to 89.5% (Figure 4h). Figure S14a (Supporting Information) showed the CV curves of the LIG‐C‐based supercapacitor under several 15° bends, collected at the same scan rate of 100 mV∙s^−1^. All CV curves exhibited nearly identical shapes. The C_A_ remained almost unchanged in the convex, concave, and platform states (Figure S14b, Supporting Information), indicating that the device held great potential for applications in flexible electronics.

The supercapacitors of this study were further connected in series and parallel (Figure S15, Supporting Information), and the results were presented in Figures S16 and S17 (Supporting Information). As shown in Figure S16a (Supporting Information), the CV curves of the series‐connected devices exhibited the pseudo‐parallelogram shapes, while the GCD curves (Figure S16b, Supporting Information) displayed the pseudo‐triangular profiles. Both of the above‐mentioned results were typical characteristics of the electric double‐layer effect. The supercapacitors connected in series could result in a reduction in the total capacitance. As illustrated in Figure S16c (Supporting Information), the experimentally measured capacitance of the series‐connected supercapacitors was lower than the theoretical value. For instance, the measured areal capacitance of five supercapacitors connected in series was 13.7 mF∙cm^−2^, slightly lower than the theoretical value (71.4 ÷ 5 = 14.3 mF∙cm^−2^). This deviation was primarily attributed to the capacitance loss induced by the interconnection among the individual supercapacitors. Nevertheless, the series configuration significantly enhanced the voltage tolerance of the circuit, thereby broadening the application potential of LIG‐based supercapacitors. In contrast, when the supercapacitors were connected in parallel (Figure S17a,b, Supporting Information), the CV and GCD curves also clearly exhibited the parallelogram and pseudo‐triangular shapes. The supercapacitors connected in parallel could lead to an increase in total capacitance. However, the measured areal capacitance of five supercapacitors connected in parallel (263 mF∙cm^−2^) was also lower than the theoretical value (71.4 × 5 = 357 mF∙cm^−2^) due to losses caused by the interconnection among the connected supercapacitors. Despite this, the parallel configuration could improve the voltage quality and reduce the energy loss, further enhancing the supercapacitor efficiency.

### 2.5 Joule Heater

Joule heaters based on LIG and LIG@MXene composite, with dimensions of 1 cm × 1 cm, were fabricated via blue laser etching. Characterization involved connecting the heating devices to an electrochemical workstation and monitoring their temperature profiles with an infrared camera (Figure S18, Supporting Information). Specifically, Figure S18a (Supporting Information) showed the resistance change curves of Joule heaters under varying DC voltages (0–6 V). Unlike metals, which typically exhibit positive resistance changes due to increased phonon scattering at elevated temperatures, LIG prepared at 55% laser power and 70 mm s^−1^ demonstrated a negative resistance change, characterized by the decreasing resistance with the increase of temperature. This phenomenon arose from graphene's semi‐metallic nature, where high temperature would improve the charge carrier mobility (electrons and holes) and thereby reduce resistance.^[^ ^58^ ^]^ Following the incorporation of MXene into the lignocellulose‐based LIG, the resistance variation under the identical applied voltages significantly expanded. At 5 V, the ΔR/R_0_ reached −58.3% for heaters based on LIG@MXene composite, while the pure LIG‐derived one was only −19.0%. Figure S18b (Supporting Information) further revealed the ΔR/R_0_ vs voltage relationships, demonstrating a strong linear correlation. The Joule heater based on pure LIG and LIG@MXene composite exhibited the *R^2^* values of 0.986 and 0.998. The comparison of temperature response curves (Figure S18c, Supporting Information) also indicated a remarkable enhancement in heating performance when incorporating MXene into the LIG matrix. When the applied voltage was 5 V, the LIG@MXene heater could reach a maximum temperature of 113 °C, 79.5% higher than that of the pure LIG‐derived one (62.8 °C). It was worth noting that directly comparing the temperature changes of LIG and LIG@MXene under the same applied voltage was inappropriate due to their differing initial resistances. Therefore, this study also employed a resistance normalization to further assess the impact of MXene incorporation on heating performance. The corresponding applied voltages for the LIG@MXene heater were calculated when 1–6 V were applied to the LIG heater, respectively. These voltages were 4.97, 4.14, 3.31, 2.48, 1.65, and 0.83 V, respectively. As shown in Figure S19 (Supporting Information), the heating performance of the LIG@MXene heater was significantly higher than that of the LIG heater after resistance normalization, further demonstrating the substantial enhancement in heating performance due to MXene incorporation. This was because MXene could decrease the electrical resistance of LIG to 17.2 Ω∙sq^−1^. Additionally, MXene integration improved the operational stability of the Joule heater, as evidenced by the *R^2^* value of the temperature‐voltage curves, where the LIG@MXene composite and pure LIG devices were 0.998 and 0.971, respectively (Figure S18d, Supporting Information). Infrared images shown in Figure S18e (Supporting Information) also consistently illustrated the higher temperature rise for the Joule heaters based on LIG@MXene composite than pure LIG under the identical voltage conditions. Furthermore, the 25 consecutive long‐term heating tests on the LIG@MXene‐based heater were performed under an applied voltage of 5 V (Figure S20, Supporting Information). The results demonstrated that the heating performance remained stable, with the equilibrium temperature maintained at ≈121 ± 4.93 °C. Subsequent characterizations of the heated Joule heater revealed a slight increase in defects (I_D_/I_G_ = 0.93), which can be attributed to the interaction with ambient oxygen at elevated temperatures (Figure S21, Supporting Information). In addition, the prolonged heating would cause a partial degradation of the lignocellulosic substrate, as clearly observed in the SEM images (Figure S22, Supporting Information). Overall, the LIG@MXene heater exhibited excellent thermal stability, confirming the enhancing effect of MXene incorporation.

### 2.6 Pressure Sensor

**Figure 5:**
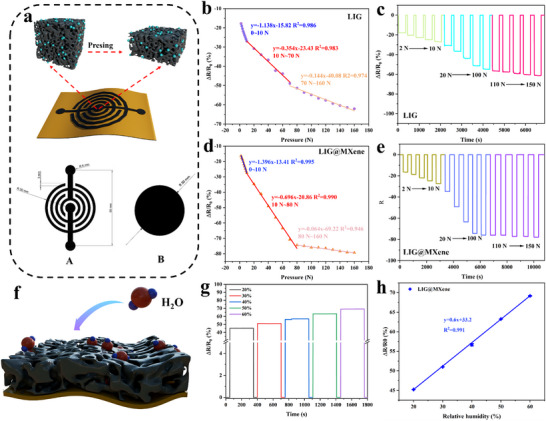
a) Working schematic of pressure sensor, b) the fitting curve of relative resistance change under external pressure and c) ΔR/R_0_ for pressure sensor based on pure LIG, d) the fitting curve of relative resistance change under external pressure and e) ΔR/R_0_ for pressure sensor based on LIG@MXene composite, f) working mechanism of humidity sensor, g) the sensor resistance response at different humidity levels and h) the corresponding fitting curve.

**Figure** **5**a illustrates the operational mechanism of pressure sensors fabricated via direct blue laser etching and patterning. In this design, surface A acted as the working layer, displaying a high resistance in the absence of applied pressure, while surface B served as the conductive layer. When pressure was applied, the contact between surfaces A and B caused an abrupt resistance decrease, with the magnitude of this decrease being directly correlated to the applied pressure. The resistance changes observed in the pressure sensors based on pure LIG (55% laser power and 70 mm s^−1^) and LIG@MXene composite under varying pressures are depicted in Figure 5b–e. Within the pressure range of 0–10 N, the pressure sensor based on LIG@MXene composite demonstrated a superior curve fitting (*R^2^* = 0.995) compared to a pure LIG‐based one (*R^2^* = 0.986). This improved fit was also evident across higher pressure ranges, including 20–100 N and 110–150 N. In the range of 0–10 N, the contact between surfaces A and B induced an abrupt resistance reduction, yielding a sensitivity of 3.95 kPa^−1^ for the pressure sensor based on LIG@MXene composite, notably higher than that of a pure LIG‐based one (3.22 kPa^−1^). This was primarily attributed to the porous framework of LIG, providing sufficient compression space, and the MXene filling, reinforcing the conductive network.^[^ ^32^ ^]^ The synergistic effect of these two factors significantly enhanced the sensitivity of the pressure sensor. When the applied pressure increased from 10 to 70 N, the sensitivity of the sensor based on LIG@MXene composite decreased to 1.97 kPa^−1^, while the sensitivity of the pure LIG‐based one was 1.0 kPa^−1^. At the higher pressures (70–160 N), the pressure sensor based on LIG@MXene composite still exhibited a significantly reduced sensitivity of 0.181 vs 0.407 kPa^−1^ for the pure LIG‐based one, indicating a reversal of performance trends in this range. This reversal might be attributed to the pressure‐induced sliding, reorganization, or densification of MXene sheets under high‐pressure conditions, leading to a stabilization of the conductive network. Ultimately, this process would enhance the electrical conductivity of the LIG@MXene pressure sensor and result in a reduced resistance variation rate. To validate this hypothesis, this study conducted a cyclic pressure test under a 160 N load for 10 000 cycles, followed by the characterization of the tested LIG@MXene composite. As shown in Figure S23 (Supporting Information), the LIG@MXene composite still demonstrated a good stability effect throughout the 10 000‐cycle test. Raman spectroscopy revealed that the LIG@MXene composite exhibited the minimal defect variation (I_D_/I_G_ = 0.903) and an increased stacking order (I_G_/I_2D_ = 1.39) (Figure S24, Supporting Information). The SEM images of the LIG@MXene composite were further examined after the 150 N pressure test (Figure S25, Supporting Information), and the corresponding surface resistances were also measured (Figure S26, Supporting Information). The pores on the surface of the LIG@MXene composite decreased under 150 N pressure, leading to a denser structure. The resistance change of LIG@MXene composite was also significantly weaker than that of pure LIG. Both of the above‐mentioned testing results supported the proposed explanation. Similar observations have also been reported in previous studies, further confirming our interpretation.^[^ ^59^, ^60^ ^]^ In contrast, the relatively rigid graphene network within pure LIG maintained a consistent resistance‐pressure response gradient under high compression.^[^ ^60^ ^]^ When the applied pressure was low, the incorporation of MXene generally enhanced the sensor's pressure response characteristics. As shown in Figure 5e, the pressure sensor based on LIG@MXene composite exhibited a substantially higher ΔR/R_0_ value than the pure LIG‐based one (Figure 5c) under the identical pressures.

### 2.7 Humidity Sensor

LIG possessed a porous and foam‐like structure that afforded strong adsorption capacity and efficient capture of atmospheric water vapor (Figure 5f). The resistance responses of the humidity sensors based on pure LIG (55% laser power and 70 mm s^−1^) and LIG@MXene composite are shown in Figure 5g and Figure S27a (Supporting Information). Both sensors exhibited increasing resistances when the environmental humidity rose because of the dissociation of water molecules. This dissociation produced the flowing protons and charge carriers, such as H_3_O^+^, leading to some changes in resistance.^[^ ^61^ ^]^ Additionally, this study used lignocellulose as the LIG precursor, which would undergo swelling when exposed to moisture.^[^ ^62^ ^]^ This swelling increased the separation between the conductive carbon‐based components, disrupting the conductive network and further contributing to the increasing resistance. The linearity of the relationship between humidity and the resistance changing rate demonstrated that the sensor based on LIG@MXene composite had a superior performance than a pure LIG‐based one (Figure 5h). The coefficients of determination for the humidity sensors based on LIG@MXene composite and pure LIG were 0.991 and 0.986 (Figure S27b, Supporting Information). The incorporation of MXene into the LIG matrix also significantly enhanced the sensitivity of the humidity sensor. This enhancement was primarily attributed to the hydrophilicity and interlayer expansion of MXene. The hydrophilic surface groups readily adsorb water molecules through hydrogen bonding, resulting in an increased interlayer spacing and a corresponding rise in electrical resistance. In addition, the porous structure of LIG offered a structural advantage by increasing the specific surface area and facilitating the rapid diffusion of water molecules, thereby further amplifying the sensing response.^[^ ^63^ ^]^ The sensitivity of humidity based on LIG@MXene composite was 0.600/%RH, markedly higher than that of a pure LIG‐based one (0.227/%RH). This substantial improvement underscored the role of MXene in enhancing both the accuracy and sensitivity of the humidity sensor's response to atmospheric humidity. In addition, a long‐term stability test of the LIG@MXene humidity sensor was performed at 40% RH for 10 h (Figure S28, Supporting Information), during which the sensor exhibited a stable performance. Subsequent characterization revealed no significant change in defect levels (I_D_/I_G_ = 0.88) after the prolonged operation, which highlighted the long‐term operational stability (Figure S29, Supporting Information). Furthermore, SEM images also showed that the porous foam‐like structure remained essentially unchanged (Figure S30, Supporting Information), providing additional evidence for the structural stability of the LIG@MXene composite.

### 2.8 Electrochemical Sensor

**Figure 6:**
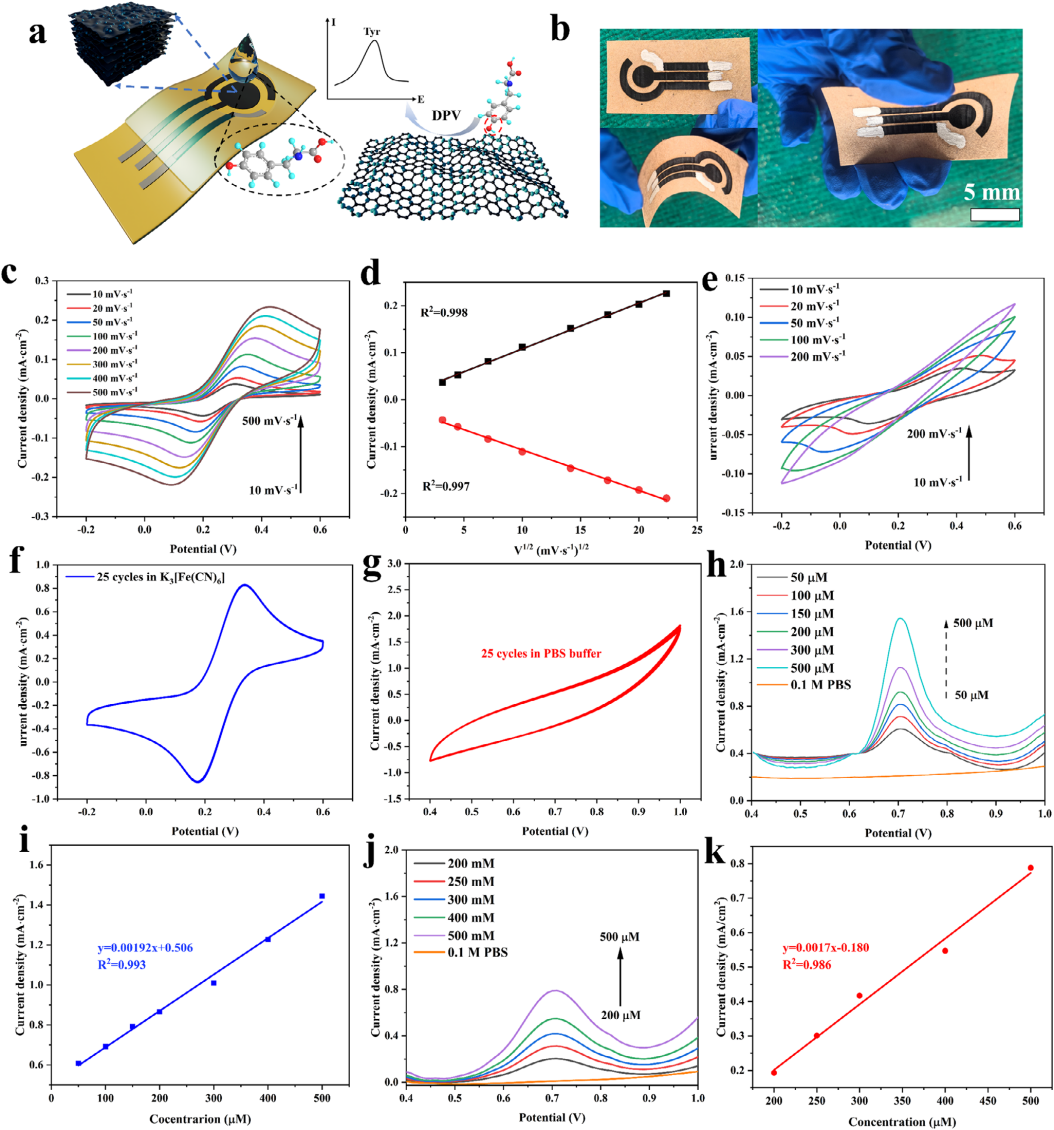
a) Working mechanism schematic of electrochemical sensor, b) photographs of flexible electrochemical chip under various bending angles, c) CV measurements of WE based on LIG@MXene composite in 5 mM K_3_[Fe(CN)_6_] and 0.1 m KCl, d) the peak current plotted vs square root of scan rate with the fitted linear regression curve in 5 mM K_3_[Fe(CN)_6_] and 0.1 m KCl, e) CV measurements of pure LIG‐based WE in 5 mM K_3_[Fe(CN)_6_] and 0.1 m KCl, f) CV recorded for 25 cycles at 50 mV∙s^−1^ scan rate for LIG@MXene‐based WE in 5 mM K_3_[Fe(CN)_6_] and 0.1 m KCl; g) CV recorded for 25 cycles at 50 mV∙s^−1^ scan rate for LIG@MXene‐based WE in 0.1 m PBS, h) DPV curves of LIG@MXene‐based WE in different Tyr concentrations and 0.1 m PBS, i) the fitting function of DPV peak currents for electrochemical sensor based on LIG@MXene composite, j) DPV curves of pure LIG‐based one in the different Tyr concentrations and 0.1 m PBS, k) the fitting function of DPV peak currents for electrochemical sensor based on LIG composite.

The concentration of Tyr in foot sweat is an important index for monitoring human health. As depicted in Figure S1 (Supporting Information), a co‐planar triple‐electrode system, consisting of a working electrode (WE), counter electrode (CE), and reference electrode (RE), was fabricated directly using direct laser writing technology. RE was constructed by applying silver paste to the composite film.^[^ ^32^ ^]^ Selective passivation with PDMS was employed to isolate the sensor region containing the active components. **Figure** **6**a presents the sensing principle of the electrochemical sensor of this study. At the interface containing sensitive LIG (55% laser power and 70 mm s^−1^) or LIG@MXene composite, Tyr molecules underwent oxidation and electron loss, resulting in changes to WE potential. This phenomenon was confirmed through differential pulse voltammetry, which validated the redox processes occurring at the modified electrode surface. Figure 6b provided a physical depiction of the electrochemical sensor, which highlighted its mechanical flexibility and showcased its ability to endure bending and twisting without the loss of structural integrity.

CV measurements were performed in a 5 mM K_3_[Fe(CN)_6_]/K_4_[Fe(CN)_6_] solution containing 0.1 m KCl at varying scan rates (10–500 mV∙s^−1^) to investigate the electrochemical characteristics of the three‐electrode system based on the fabricated sensor (Figure 6c). Figure 6d showed a strong linear correlation between the anodic (I_pa_) and cathodic (I_pc_) peak currents and the square root of the scan rate (v^1/2^). This linear relationship suggested that the redox reaction of [Fe(CN)_6_]^3‐/4−^ at the WE was diffusion‐controlled, consistent with the Randles–Sevcik equation.^[^ ^64^ ^]^ The linear regression equations were I_pa_ (mA cm^−2^) = 0.011 + 8.75 × 10^−3^ × v^1/2^ (mV s^−1^) (R^2^ = 0.998) and I_pc_ (mA cm^−2^) = −0.021–8.64 × 10^−3^ × v^1/2^ (mV s^−1^) (R^2^ = 0.997), indicating the efficient electron transfer within the three‐electrode system based on based on LIG@MXene composite.^[^ ^65^ ^]^ In comparison, the pure LIG‐based one showed lower *R^2^* values of 0.990 and 0.994 for I_pa_ and I_pc_, respectively (Figure S31, Supporting Information). Randles–Sevcik equation, often assuming the diffusion‐only mass transport, was used to estimate the ESA of the three‐electrode system based on based on LIG@MXene composite, which was 2.92 times larger than its actual geometric area. This value surpassed the currently reported ESA in the literature (e.g., 1.8 times), due to the enhanced current density from increased surface area and reduced electron transfer impedance.^[^ ^66^ ^]^ Nicholson analysis (Figure S32, Supporting Information) further revealed that the HET coefficient (k_0_) for the electrochemical sensor based on LIG@MXene composite was 11.3 × 10^−2^ cm^2^ s^−1^, significantly faster than that of the pure LIG‐based one (k_0_ = 1.97 × 10^−2^ cm^2^ s^−1^). Figure 6e presents the diminishing oxidation and reduction peaks at higher scan rates (>100 mV∙s^−1^), suggesting the poor electrochemical redox activity in the pure LIG‐based sensor.

Continuous CV tests conducted on the three‐electrode system in K_3_[Fe(CN)_6_] solution and PBS buffer (50 mV∙s^−1^) showed a stable electrochemical response over 25 consecutive cycles without performance degradation (Figure 6f,g). Additionally, DPV measurements were performed to detect Tyr at varying concentrations for both electrochemical sensors based on pure LIG and LIG@MXene composite. Figure 6h,j showed the DPV curves at different Tyr concentrations. The peak values of the electrochemical sensor based on LIG@MXene composite vs Tyr concentrations are fitted in Figure 6i, exhibiting a good linearity when Tyr concentration ranges from 50 to 500 µM (R^2^ = 0.993). In comparison, the pure LIG‐based one showed a narrower response (Figure 6k) when the Tyr concentration range of 200–500 µM (R^2^ = 0.986). The LOD value for the electrochemical sensor based on the LIG@MXene composite for Tyr sensing, calculated using the SD and S of the fitted curve at a Tyr concentration of 50 µM, was determined to be 9.6 µM, which is lower than the detection limits reported in other literature (Table S6, Supporting Information). The value of the pure LIG‐based one was only 49.7 µM. Additionally, the electrochemical sensor based on LIG@MXene composite also exhibited a higher sensitivity (0.0192 mA∙µM^−1^∙cm^−2^) than that of the pure LIG‐based one (0.017 mA∙µM^−1^∙cm^−2^). These improvements highlighted that the incorporation of MXene into the LIG matrix enhanced the electrochemical properties and enabled the wider detection ranges, lower limits of detection, and higher sensitivity for Tyr monitoring. In addition, this study also tested the bending performance of the electrodes. As shown in Figure S33, Supporting Information, the electrochemical performance of the electrodes was not significantly affected by torsional deformation, as deduced from the nearly unchanged peak current, thus indicating the remarkable flexibility and stability of the electrodes. The exceptional flexibility and stability rendered the LIG@MXene electrodes advantageous for potential applications in wearable devices. These phenomena were primarily attributed to the introduction of MXene, which provided the abundant ─O and ─OH functional groups, thereby increasing the number of redox‐active sites. In addition, the incorporation of MXene would also reduce the electrical resistance of the composite, facilitating faster electron transfer.^[^ ^32^ ^]^

### 2.9 Integrated Foot Monitoring System

**Figure 7:**
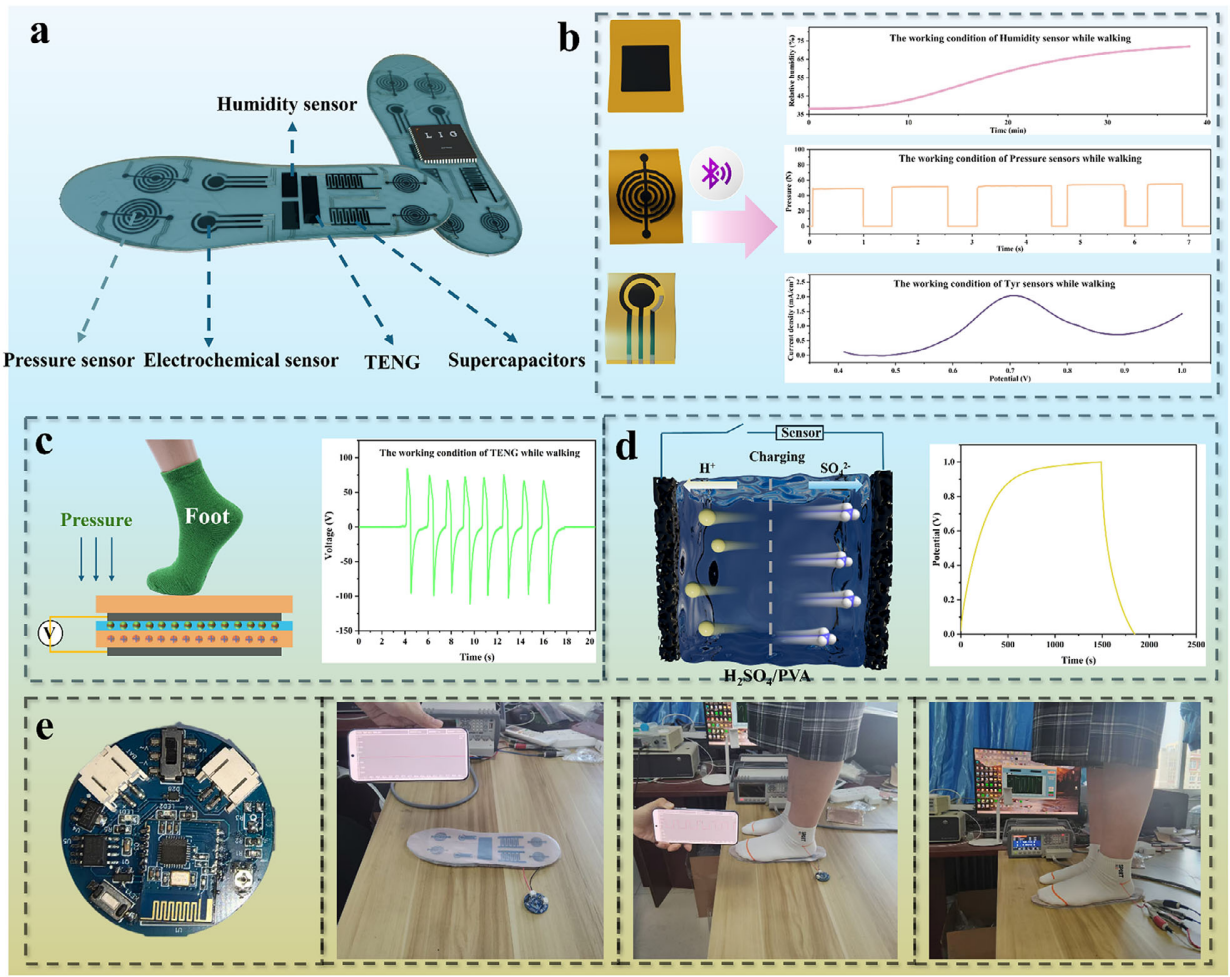
a) Functional layout of the smart insole, b) schematic of the sensor for pressure, humidity, and sweat of the foot, working mechanism of c) TENG and d) supercapacitor modules, e) schematic of the signal conversion device and signals received by the mobile phone and LCR bridge when a human foot is on the insole.

The above‐mentioned devices based on LIG@MXene all exhibited notable advantages, including exceptional flexibility, high sensitivity, and a broad detection range, making them well‐suited for monitoring various physiological signals of the foot. As shown in **Figure** **7**a, the designed flexible smart insole integrates multiple functional modules, including TENG, supercapacitor, Joule heater, and a sensor for detecting the pressure, humidity, and sweat of the foot. In this monitoring system, TENG and supercapacitor modules could enable the self‐powered operation. TENG module utilized the foot motion to drive contact‐separation cycles within the dual‐electrode structure (Figure 7c). The piezoelectric effects within the PTFE layer generated the electrical charges, which were then stored in the supercapacitor module. The working device and its mechanism are shown in Figure S34 (Supporting Information). The TENG generated current through the triboelectric effect, which was subsequently rectified by a bridge rectifier to charge the supercapacitor (Video S1, Supporting Information). As illustrated in Figure 7d, the generated charges induced the migration of H^+^ and SO_4_^2−^ ions within the electrolyte toward the LIG@MXene electrodes, thereby effectively storing electrical energy for powering the heater and sensor modules. Data collected by these sensors were transmitted wirelessly via a Bluetooth module to a signal conversion unit (Figure S35, Supporting Information). Figure S36 (Supporting Information) illustrates the process of TENG charging the supercapacitor. The experimental results showed that after 807 s of charging, the voltage of the supercapacitor reached 2.89 V, exceeding the 2.5 V required for the normal operation of the Bluetooth signal conversion device, indicating that it was sufficient to support the functioning of this module. After connecting the fabricated flexible sensor module to the supercapacitor (Figure 7b; Video S2, Supporting Information), the self‐powered system could operate normally and accurately provide gait data during human motion, monitoring foot physiological parameters. Furthermore, the self‐powered module was connected to the humidity sensor using the same method (Figure S37, Supporting Information). The humidity sensor operated normally and responded to different relative humidity levels. The self‐powered system could also directly supply power to the Joule heater (Video S3, Supporting Information). As shown in Figure S38 (Supporting Information), the Joule heater stabilized at around 35 °C after power was applied, which helped maintain the foot temperature and avoid hazards such as frostbite. The normal operation of these devices could improve human life and maintain health. For example, the Joule heater module can relax tired feet during walking or running. Meanwhile, the humidity sensor module can effectively track changes in relative humidity within the footwear environment. The sweat sensor module can also analyze foot sweat composition in real time, providing valuable insights into physiological changes and supporting potential interventions for disease prevention. A key feature of this monitoring system is its ability to synchronize all sensing signals in real time and transmit them to mobile devices via Bluetooth. The accompanying signal conversion unit is illustrated in Figure 7e, and the design details are shown in Figure S39 (Supporting Information). Preliminary testing, conducted using simplified insole prototypes interfaced with mobile devices and LCR meters, verified the satisfactory detection performance.

### 2.10 Life Cycle Assessment

**Figure 8:**
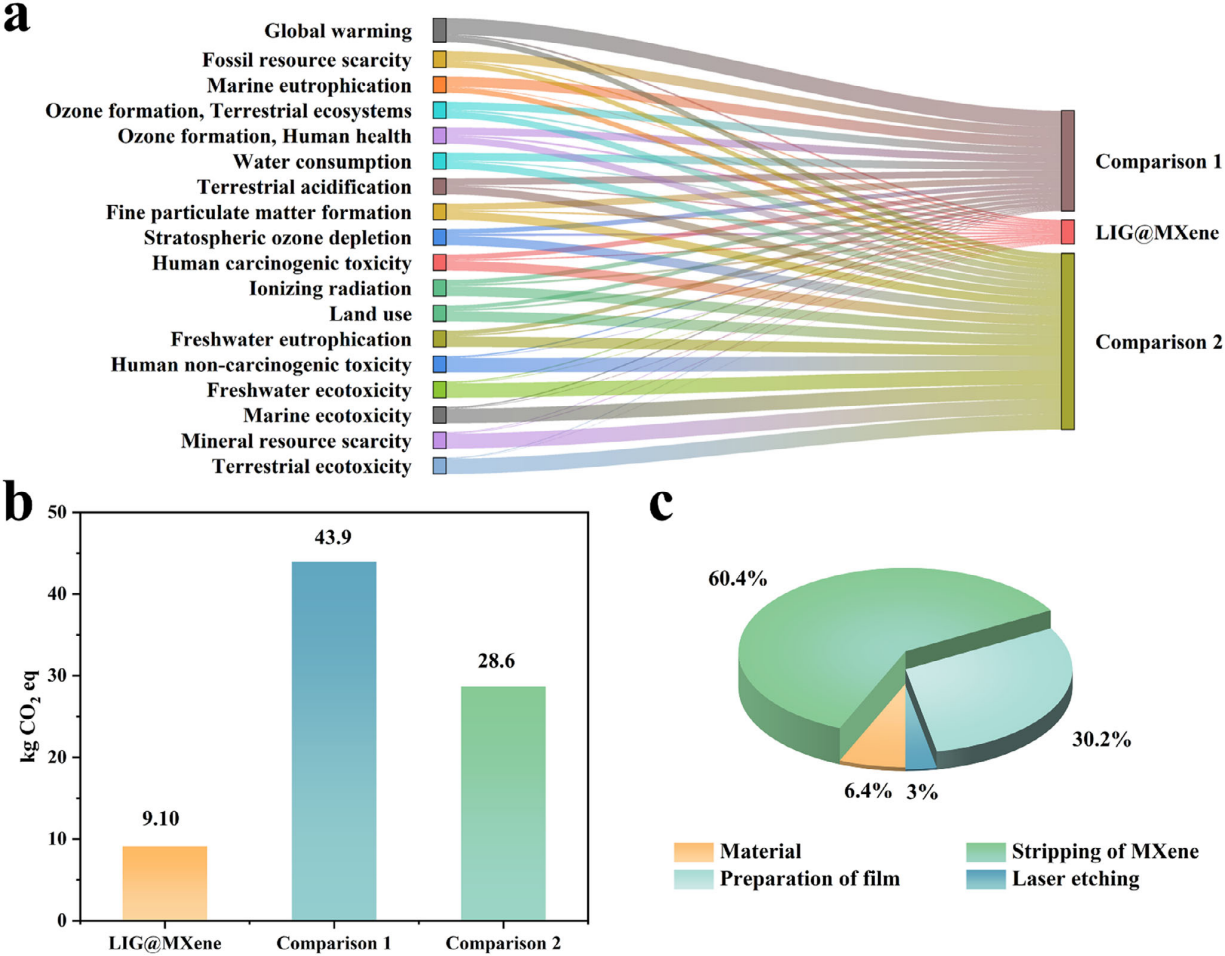
a) Environmental impact of the smart insole based on LIG@MXene composite as compared to two other similar insoles, b) the greenhouse gas emissions from the production of three smart insoles, c) the greenhouse gas emissions from each production step of the smart insole based on LIG@MXene composite.

By comparing the self‐powered smart insole based on LIG@MXene composite with two other similar insoles, it was found that the fabrication of a size 42 (≈150 cm^2^) smart insole using direct laser writing technology could significantly reduce the environmental impacts across all metrics (**Figure** **8**a). For instance, the LIG@MXene one emitted only 9.10 kg CO_2_ eq, which was 79.3% and 67.6% lower than the comparative insole 1 (43.9 kg CO_2_ eq) and 2 (28.1 kg CO_2_ eq), as depicted in Figure 8b. This demonstrated that direct laser writing technology had a considerably greener manufacturing method. During the production of the smart insole based on LIG@MXene composite, the largest source of greenhouse gas emissions was from the MXene exfoliation (Figure 8c), particularly the freeze‐drying step at −80 °C. However, the recent advancements in rapid single‐layer MXene preparation have shown promise in mitigating these emissions. Future refinement of MXene fabrication processes is expected to make production not only more environmentally friendly but also more accessible. For practical scenarios involving large‐scale implementation, broader considerations are necessary, including overall remediation efficiency, material availability, and economic cost. The integrated self‐powered smart insole for continuous foot health monitoring based on LIG@MXene composite can offer the long‐term feasibility in large applications with the consideration of a balance between environmental sustainability and economic viability.

## 3 Conclusion

This paper demonstrated a novel method for fabricating laser‐induced MXene composite graphene via advanced direct laser writing technology, and systematically explored its morphogenesis process and lattice construction mechanisms. LIG@MXene composite was successfully prepared by incorporating MXene into the graphene lattice. After optimizing the laser processing parameters, the fabricated LIG@MXene composite exhibited a low sheet resistance (17.2 Ω∙sq^−1^) and excellent chemical stability. Based on this optimal LIG@MXene composite, various functional devices were developed, including a double‐electrode TENG with a maximum power generation density of 35 V cm^−2^, a supercapacitor with an impressive surface capacitance of 71.4 mF cm^−2^, a Joule heater with a maximum heating temperature of 113 °C, and sensors for pressure, humidity, and Tyr in sweat. The minimum detection limit of Tyr was 9.6 µM. The above‐mentioned devices were integrated into a self‐powered smart insole for continuous foot health monitoring. LCA was used to underscore the environmental advantages of producing this smart insole, which confirmed that the greenhouse gas emission was only 9.10 kg CO_2_ eq. LIG@MXene composite of this study demonstrated an exceptional performance, versatility, and environmental benefits, making it a potential candidate for manufacturing wearable devices for health monitoring.

## 4 Experimental Section

### Materials

The lignocellulose films (0.1 mm thickness) were purchased from Guochu Technology (Xiamen) Co., Ltd. Dimethyl sulfoxide (DMSO, AR) was obtained from Shanghai Macklin Biochemical Technology Co., Ltd. Conductive silver paint (Product No. 05001‐AB) was procured from SPI Supplies (USA). Ag/AgCl paste (Product No. S487443) was acquired from Shanghai Aladdin Biochemical Technology Co., Ltd. Polytetrafluoroethylene (PTFE) membranes (80 µm thickness) were supplied by Sifu New Materials (Suzhou) Co., Ltd. Nylon membranes (0.45 µm pore size) were purchased from Amersham Biosciences (USA). The 0.1 m phosphate buffered saline (PBS, PH = 6.8) solution and L‐tyrosine (Tyr, AR) were both sourced from Shanghai Macklin Biochemical Technology Co., Ltd. The nitrogen‐phosphorus flame retardant (C_12_H_29_N_4_O_5_P_3_S_2_, 98%) was obtained from the same supplier. MXene powder was provided by XFNANO Materials Technology Co., Ltd. Polyvinyl alcohol (PVA, MW = 80 000–110 000), KCl (analytical grade), and polydimethylsiloxane (PDMS) were all purchased from Shanghai Macklin Biochemical Technology Co., Ltd. H_2_SO_4_ (98%) was acquired from Xilong Scientific Co., Ltd., while Dragon Skin was procured from Beijing Tiantong Huayi Technology Development Co., Ltd.

### Fabrication of LIG

Lignocellulose film was trimmed into square substrates with 10 cm edge lengths, which were then immersed in a flame‐retardant solution for 5 min as part of a pretreatment process. After immersion, the specimens were retrieved and placed into a thermostatic drying oven, where they were dried at 25 °C for 3 h under atmospheric pressure. The thermally stabilized substrates were then carefully aligned beneath a computer‐controlled laser emission system (Xtool D1 Pro, Shenzhen Maker Workshop Technology Co., Ltd., China) with the following parameters: maximum power of 5W, spot size of 0.06 mm, and the laser wavelength of 455 nm. Systematic parameter optimization was conducted by adjusting laser power intensities (35%, 45%, 55%, 65%, and 75% of the maximum 5 W output) in combination with programmable scanning velocities (40, 55, 70, 85, and 100 mm∙s^−1^), with the objective of achieving enhanced crystallinity and reduced defect density in the final carbonized architecture.

### Delamination of Ti_3_C_2_Tx MXene

Delamination was performed using DMSO as the intercalant, which resulted in an increased interlayer spacing of MXene. Initially, 1 g of MXene powder was dispersed in 20 mL of DMSO and stirred for 24 h at room temperature. The resulting colloidal suspension was then centrifuged (4000 rpm, 5 min) to separate the intercalated MXene powder. After the suspension was collected, deionized water was added, followed by bath sonication for 6 h. The suspension was centrifuged again, and the sediments were collected via vacuum‐based filtration. The resulting precipitate was mixed with deionized water at a mass ratio of 1:200 and sonicated for 2 h. The resulting supernatant was frozen at −80 °C for 2 h and then lyophilized in a freeze dryer for 48 h. Through this process, monolayer MXene powder was successfully obtained.^[^ ^33^, ^67^ ^]^

### Fabrication of LIG@MXene Hybrid

The lignocellulose film was initially immersed in flame‐retardant solution for 5 min, followed by drying in a thermostatic drying oven at 25 °C for 3 h. The exfoliated monolayer MXene powder, constituting 5 wt.% of the total mass, was dispersed into deionized water and subjected to 20 min of ultrasonication to achieve colloidal uniformity. The resultant homogeneous suspension was then uniformly coated onto the pretreated surface of lignocellulose substrate, with subsequent drying conducted at 25 °C for 30 min under controlled atmospheric conditions. Finally, the dried composite precursor was laser‐etched using a blue laser system to prepare the LIG@MXene composite. The fabricated LIG@MXene was assembled into various devices and then tested under standard atmospheric conditions at 25 °C and 50% relative humidity. For the humidity sensor, the relative humidity was precisely controlled during the measurements.

### Characterization

The sheet resistance of LIG was measured by a four‐probe square resistivity meter. The surface morphology of LIG samples was observed using a scanning electron microscope (SEM, HITACHI S‐4800). The grid spacing of LIG was observed by an electron transmission microscope (TEM, FEI Tecnai G2 F30). The surface elemental composition of the LIG was analyzed using X‐ray photoelectron spectroscopy (XPS, Thermo escalab 250XI). The LIG powder sample was also characterized by an X‐ray diffractometer (XRD, D8 Advance X) with a scanning rate of 0.02° per step from 3° to 160°. Raman analysis of all samples was conducted using Raman spectroscopy (RENISHAW).

Raman spectra data were used to calculate the crystalline size of LIG in the a‐axis (L_a_) from the ratio of the intensity of the G peak (I_G_) and the D peak (I_D_) by Equation (1).^[^ ^68^ ^]^

(1) $$L_{a}=\left(2.4\times 10^{-10}\right)\times {\lambda_{I}}^{4}\times \frac{I_{G}}{I_{D}}$$

where λ_I_ is the wavelength of the Raman laser (532 nm).

### Fabrication of TENG

After the LIG (2 × 2 cm^2^) was fabricated, the graphene layer's surface was covered with a PTFE film, serving as both the negative electrode and one friction interface of TENG. Concurrently, another pristine LIG layer acted as the positive electrode and the other friction interface. The PTFE film of the negative electrode and the residual polyimide layer of the positive electrode were brought into contact to form the friction pair. Finally, a nylon film was applied to the outer surface of both the positive and negative electrodes, and an acrylic plate was placed atop both nylon films to create a load‐transferring layer. To enable data collection, conductive silver paint was uniformly applied to the conductive side of the LIG, followed by the application of copper adhesive tape onto the coated surface, which was interfaced with an electrometer.

### Fabrication of Supercapacitor

The supercapacitors were fabricated with 12 interdigitated electrodes, each measuring 1 mm in height × 6 mm in width, with a 1 mm gap between adjacent interdigitated electrodes. Subsequently, a polymeric hydrogel (PVA/H_2_SO_4_) electrolyte was prepared by mixing 1 g of PVA with 10 mL of Deionized water and stirring continuously for 3 h at 80 °C before adding 1 mL of H_2_SO_4_ dropwise to create a transparent gel‐like electrolyte. Finally, the PVA/H_2_SO_4_ hydrogel was uniformly applied to the IDE before being used as a micro‐supercapacitor device. The supercapacitor performance was tested by an electrochemical workstation (CHI 660E, CH Instruments, China). Cyclic voltammetry (CV) potentiostatic measurements were conducted under an operational voltage range of 0–1 V at varying scan rates (10, 20, 50, 100, and 200 mV∙s^−1^). The CV scans comprised 12 segments, with the data from the final cycle being selected for subsequent processing. Galvanostatic charge–discharge (GCD) measurements were simultaneously performed at distinct current densities (0.05, 0.1, 0.2, 0.5, and 1 mA∙cm^−2^) within the identical voltage window (0–1 V), where both the upper and lower voltage limits were maintained at 0 s holding time. The GCD test protocol included 12 scanning segments, and analogous to the CV analysis, the last cycle's data were exclusively utilized for evaluation. The areal capacitance (C_A_, mA∙cm^−2^), derived from both CV and GCD curves, was calculated through Equations (2) and (3), respectively.^[^ ^18^ ^]^

(2) $$C_{A}=\frac{\int idV}{2\times S\times v\times \Delta V}$$

(3) $$C_{A}=\frac{I}{S\times \frac{dV}{dt}}$$

where, *S* is the surface area (cm^2^) of the LIG electrodes, *v* is the voltage scan rate (V/s), ∆*V* is the voltage range (V), *i* is the voltammetry current response (A) during CV scan, ∫*i*dV is the integrated area of CV curve, *I* is the discharge current (A), and d*V*/d*t* is the slope of galvanostatic discharge curve.

The total C_A_ of supercapacitors connected in series can be calculated as:

(4) $$\frac{1}{C_{A}}=\frac{1}{C_{1}}+\frac{1}{C_{2}}+\frac{1}{C_{3}}+\cdot \cdot \cdot \;\frac{1}{C_{n}}=\sum_{i=1}^{n}\left(\frac{1}{C_{i}}\right)$$

where C_i_ is the capacitance of each unit. For first‐order approximation, C_1_ = C_2_ = C_3_ = C_i_, and this results in C_A_ = C_1_/n.

The total C_A_ of supercapacitors connected in parallel can be calculated as:

(5) $$C_{A}=C_{1}+C_{2}+C_{3}+\cdot \cdot \cdot C_{n}=\sum_{i=1}^{n}C_{i}$$

where C_i_ is the capacitance of each unit. For first‐order approximation, C_1_ = C_2_ = C_3_ = C_i_, and this results in C_A_ = nC_1_.

### Fabrication of Joule Heater

A blue laser etching machine was used to fabricate LIG with dimensions of 2 cm on the surface of pretreated lignocellulose substrate. Subsequently, both sides of the LIG were uniformly coated with conductive silver paint and extended with copper tape. Once the two ends were connected to an electrochemical workstation, electric power was supplied under varying direct current (DC) voltages of 1, 2, 3, 4, 5, and 6 V. The thermal images of the stabilized LIG‐based joule heater at different voltages were captured using an infrared camera (ICI 8000 Series‐P, Infrared Cameras Inc., Reno, NV, USA). Additionally, the temperature variation data during the voltage application was recorded by an infrared thermometer (E40, FLIR Systems Inc., USA).

A reference power was selected, and by using the equation P = V^2^/R, the applied voltage V_MXene_for the LIG@MXene Joule heater was derived as follows:

(6) $$V_{MXene}=\sqrt{\frac{{V_{LIG}}^{2}\times R_{Mxene}}{R_{LIG}}}$$

where V_LIG_ is the voltage applied to the LIG Joule heater, and R_LIG_ and R_MXene_ represent the sheet resistances of LIG and LIG@MXene, respectively.

### Fabrication of a Pressure Sensor

A work surface (A) and a conductive surface (B) were etched onto lignocellulose substrate using a blue laser system. Conductive silver paint was then uniformly applied to both lateral sides of the LIG structure on surface A, with electrical contacts extended via copper tape application. On the reverse side of the LIG, the *Dragon Skin* polymer solution (Part A/Part B = 1:1, w/w) was spin‐coated at 3000 rpm, followed by curing overnight in a thermostatic drying oven at 25 °C. Upon complete crosslinking of the polymer matrix, surfaces A and B were precisely aligned and assembled. The sensor's electrical contacts were interfaced with an LCR meter (TH2830, Changzhou Tonghui Electronic Co., Ltd., China), and its pressure‐dependent electrical responses were evaluated systematically using a calibrated pressure testing apparatus. The sensitivity of the pressure sensor was subsequently quantified.^[^ ^32^ ^]^

(7) $$s=\frac{\Delta R}{R_{0}\Delta P}\times 100\%\;=\frac{\Delta R}{R_{0}}\times \frac{s}{\Delta F}\times 100\%$$

where R_0_ represents the initial resistance, ΔR signifies the difference between the real‐time resistance value and the initial value R_0_, ΔP and ΔF indicate the amount of pressure and stress change, respectively, and s represents the area of force application.

### Fabrication of Humidity Sensor

A blue laser etching machine was used to fabricate 2 × 2 cm^2^ LIG on the pretreated surface of lignocellulosic substrate, followed by the uniform coating of both sides of the LIG with conductive silver paint. Copper tape was subsequently applied to extend the electrical connections, and the terminals were interfaced with an LCR meter. The assembled device was tested in a humidity chamber (RSR0320‐040PSE‐5, Tinghua Instrument Co., Ltd., China) to systematically measure resistance variations across various relative humidity (RH) levels (20%, 30%, 40%, 50%, and 60%). The sensitivity of the humidity sensor was quantified as follows:

(8) $$s=\frac{\Delta R}{R_{0}\Delta RH}\times 100\%$$

where R_0_ represents the initial resistance, ΔR signifies the difference between the real‐time resistance value and the initial value R_0_, and ΔRH indicates the amount of relative humidity.

### Fabrication of an Electrochemical Sensor

The LIG@MXene electrodes were fabricated according to the shape and dimensions specified in Figure S1 (Supporting Information), followed by the uniform coating of a PDMS layer while leaving the working area exposed. The coated electrodes were then cured in air drying oven at 25 °C for 5 h. Ag/AgCl paste was selectively applied to one electrode and dried for 20 min. An electrolyte solution was prepared by dissolving varying amounts of Tyr in 100 mL of a 0.1 m PBS buffer (pH = 6.8), creating concentration gradients of 50, 100, 150, 200, 300, and 500 µM. Electrochemical measurements were performed using a three‐electrode system to evaluate the concentration‐dependent responses. CV parameter setting as follows: the range of potential, scan rate, and scan segment was set as 0.4–1.0 V, 10 to 200 mV∙s^−1^, and 12 segments, respectively. DPV parameter setting as follows: the range of potential, pulse amplitude, pulse time, and scan rate was set as 0.4–1.0 V, 50 mV, 50 ms, and 8 mV∙s^−1^, respectively. The peak height of the DPV and CV curves was obtained from the software of the electrochemical workstation. The limit of detection (LOD) of the Tyr biosensor was calculated by Equation (9).

(9) $$LOD=3\times \left(\frac{SD}{SL}\right)$$

where SD is the standard deviation of the response to different Tyr concentrations, and SL is the slope of the fitted calibration plots.

The electrochemically active surface area (A) of LIG electrodes was estimated based on the Randles–Sevcik equation.^[^ ^64^ ^]^

(10) $$I_{P}=2.69\times 10^{5}A\times D^{\frac{1}{2}}n^{\frac{3}{2}}v^{\frac{1}{2}}C$$

where n is the number of electrons in the redox reaction, D is the diffusion coefficient of the molecule (D = 7.63 × 10^−6^ cm^2^∙s^−1^ for [Fe(CN)_6_]^3−^), C is the concentration of the probe molecule in the solution (mol∙cm^−3^), and v is the scan rate (V∙s^−1^). The ratio of I_p_ to v^1/2^ was determined by the slope of the linear fitting curve, which resulted in the estimated electrochemically active surface area (A).

Nicholson analysis was used to determine the heterogeneous electron transfer (HET) rate (k^0^) according to the following equation:^[^ ^69^ ^]^

(11) $$\Psi =k^{0}{\left(\frac{D_{O}}{D_{R}}\right)}^{\frac{\alpha }{2}}\sqrt{\frac{RT}{\pi nFD_{O}v}}$$

where Ψ is the dimensionless kinetic parameter related to peak potential separation ΔE_P_, and ΔE_P_ is the potential difference between the anodic and cathodic peaks. D_O_/D_R_ is the diffusion coefficient ratio of the oxidized to the reduced form of the electroactive species, α is the transfer coefficient, F = 96489 C∙mol^−1^ is the Faraday constant, R is the universal gas constant (8.314 J∙K^−1^∙mol^−1^), and T is the absolute temperature. Assuming the approximately equal D_O_ and D_R_ and symmetrical redox kinetics (α≈0.5), simplified Equation (12) to:

(12) $$\Psi =Ck^{0}v^{-\frac{1}{2}}$$

The dimensionless kinetic parameter Ψ was calculated from the following equation for a one‐electron process.

(13) $$\Psi =\frac{\left(-0.6288+0.0021\Delta E_{P}\times n\right)}{1-0.017\Delta E_{P}\times n}$$

Therefore, k^0^ was determined from the slope of Ψ vs Cv^−1/2^.

### Life Cycle Assessment

This paper conducted a life cycle assessment (LCA) for evaluating the preparation process of LIG@MXene‐based smart insole in accordance with *ISO 14040* standards and performed a comparative analysis with two other similar insole systems. The detailed materials and energy inventory required to produce a single size 42 insole (≈150 cm^2^) is provided in Tables S1–S3 (Supporting Information). Data on material and energy inputs for the production of various smart insoles were sourced from the Ecoinvent database V3.11, enabling a quantitative assessment of the overall environmental impact. The ReCiPe Midpoint (H) V1.07 method was used for the comparative analysis across the different systems, focusing on the contribution proportions of significant inputs within each system.

## Conflict of Interest

The authors declare no conflict of interest.

## Supporting information



Supporting Information

Supplemental Video 1

Supplemental Video 2

Supplemental Video 3

## Data Availability

Research data are not shared.
