# Supplementary material for: Integrated Self‐Powered Sensors for Continuous Foot Health Monitoring via Laser‐Induced MXene‐Composited Graphene Hybrids From Lignocellulose
Source: Adv Sci (Weinh). 2025 Oct 22;13(2):e16691. doi: 10.1002/advs.202516691 (PMC12786368; doi:10.1002/advs.202516691)
Supplement: Supplementary file 1 — Supporting Information [file ADVS-13-e16691-s003.docx]

**Integrated Self-Powered Sensors for Continuous Foot Health Monitoring via Laser-Induced MXene-Composited Graphene Hybrids**

Peilong Zhao ^a, b^, Xiaofei Mao ^a^, Jiashu Song ^a^, Man Liu ^a^, Luxue Cui ^a^, Mingyang Liu ^b^, Nan Zhao ^a,^ *, Yuguang Zhou ^b^

^a^ School of Ecology and Environment, Zhengzhou University, Zhengzhou 450001, China

^b^ College of Engineering, China Agricultural University, Beijing 100083, China

^*^ Corresponding author: Nan Zhao, PhD, Professor, Tel: +86 13838315063, E-mail address: [nan.zhaoca@outlook.com](mailto:nan.zhaoca@outlook.com)/zhaonan@zzu.edu.cn

**List of contents**

**Figure S1.** Schematic diagram of electrochemical sensor.

**Figure S2.** SEM images of lignocellulose and LIG.

**Figure S3.** Lignocellulose-based LIG laser parameter optimization characterization.

**Figure S4.** C1s spectra of lignocellulose-based LIG under different laser parameters.

**Figure S5.** SEM images of MXene before and after exfoliation.

**Figure S6.** Current of the TENG on the applied force.

**Figure S7.** Output voltage, current and Instantaneous output power density with a vertical force of 4 N as a function of the load resistance.

**Figure S8.** Charging curve of a 25 μF capacitor by the TENG through a bridge rectifier.

**Figure S9.** Performance testing of lignocellulose-based LIG supercapacitors at different laser power.

**Figure S10.** Performance tests of lignocellulose-based LIG supercapacitors at different etching rates.

**Figure S11.** Performance test of LIG supercapacitors fabricated at 55% laser power and a laser etching rate of 70 mm∙s^-1^.

**Figure S12.** The CA of CA of LIG@MXene and LIG supercapacitors.

**Figure S13.** Equivalent circuit used to fit EIS spectra.

**Figure S14.** Performance of LIG@Mxene supercapacitors under 15° bending.

**Figure S15.** Schematic diagrams of series and parallel connection of supercapacitors.

**Figure S16.** Performance of LIG@MXene based supercapacitors in series.

**Figure S17.** Performance of parallel supercapacitors based on LIG@MXene.

**Figure S18.** Performance tests of lignocellulose-based LIG and LIG@MXene joule heaters.

**Figure S19.** Performance Comparison of LIG and LIG@MXene Joule Heaters after Resistance Normalization.

**Figure S20.** Temperature profile of the LIG@MXene Joule heater under 5 V during 25 heating cycles (each cycle consists of 300 s voltage application and 300 s cooling at room temperature).

**Figure S21.** Raman spectra of the LIG@MXene Joule heater under 5 V after 25 heating cycles.

**Figure S22.** SEM image of the LIG@MXene Joule heater after 25 heating cycles at an applied voltage of 5 V.

**Figure S23.** Cycle-dependent pressure response of the LIG@MXene pressure sensor over 10,000 loading–unloading cycles under a constant 160 N force (inset: enlarged view of a local segment).

**Figure S24** Raman spectra of the LIG@MXene pressure sensor after 10,000 tests at 160N pressure.

**Figure S25.** SEM image of the LIG@MXene pressure sensor after 10,000 tests at 160N pressure.

**Figure S26.** Sheet resistance of the LIG@MXene pressure sensor after testing at different pressures.

**Figure S27.** Performance testing of humidity sensors.

**Figure S28.** The resistance change of the LIG@MXene humidity sensor was tested at 40%RH for 10 hours.

**Figure S29.** Raman spectra of the LIG@MXene humidity sensor after 10 hours of testing at 40% RH.

**Figure S30.** SEM image of the LIG@MXene humidity sensor after 10 hours of testing at 40% RH.

**Figure S31.** The relationship between the peak current and the square root of the scanning rate of the LIG electrochemical sensor at 5 mM K_3_[Fe(CN)_6_] and 0.1 M KCl was plotted using fitted.

**Figure S32.** The linear fitting curve for calculating the HET constant.

**Figure S33.** Peak currents for different bending states of the sensor in 5 mM K₃[Fe(CN)₆]/K₄[Fe(CN)₆] and 0.1 M KCl, tested at a scan rate of 10 mV∙s^-1^ with the electrode bent.

**Figure S34.** Schematic diagram of the self-powered system device

**Figure S35.** A working schematic diagram of smart insoles.

**Figure S36.** TENG charged the supercapacitor for 2,600 seconds.

**Figure S37.** The test data after the self-powered module was connected to the humidity sensor.

**Figure S38.** The test data after the self-powered module was connected to the Joule heater.

**Figure S39.** Schematic diagram of the working design of the signal conversion device.

**Table S1.** Inventory for 1 LIG@MXene based smart insole.

**Table S2.** Inventory for 1 S-POF smart insole.

**Table S3.** Inventory for 1 FRdL-insole smart insole.

**Table S4.** The performance of the TENG based on LIG@MXene was compared with other reported TENG.

**Table S5.** The performance of the supercapacitor based on LIG@MXene was compared with other reported supercapacitor.

**Table S6.** The performance of the single-layer piezoresistive pressure sensor based on LIMG was compared with other reported sensors.

**Video S1.** The TENG charges the supercapacitor through a rectifier bridge.

**Video S2.** TENG powers the Bluetooth device and enables the pressure sensor to operate through a rectifier bridge.

**Video S3.** The TENG supplies power to the Joule heater through a rectifier bridge.


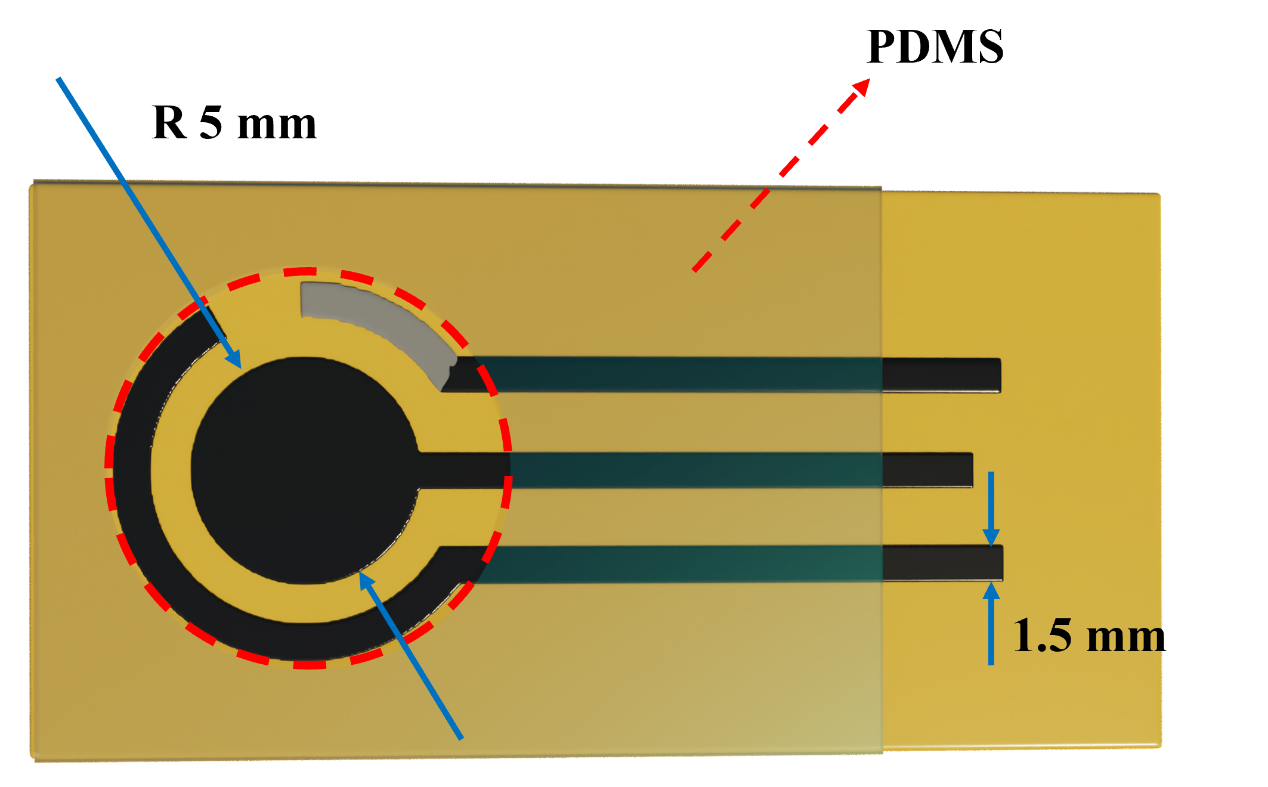


**Figure S1** Schematic diagram of electrochemical sensor.


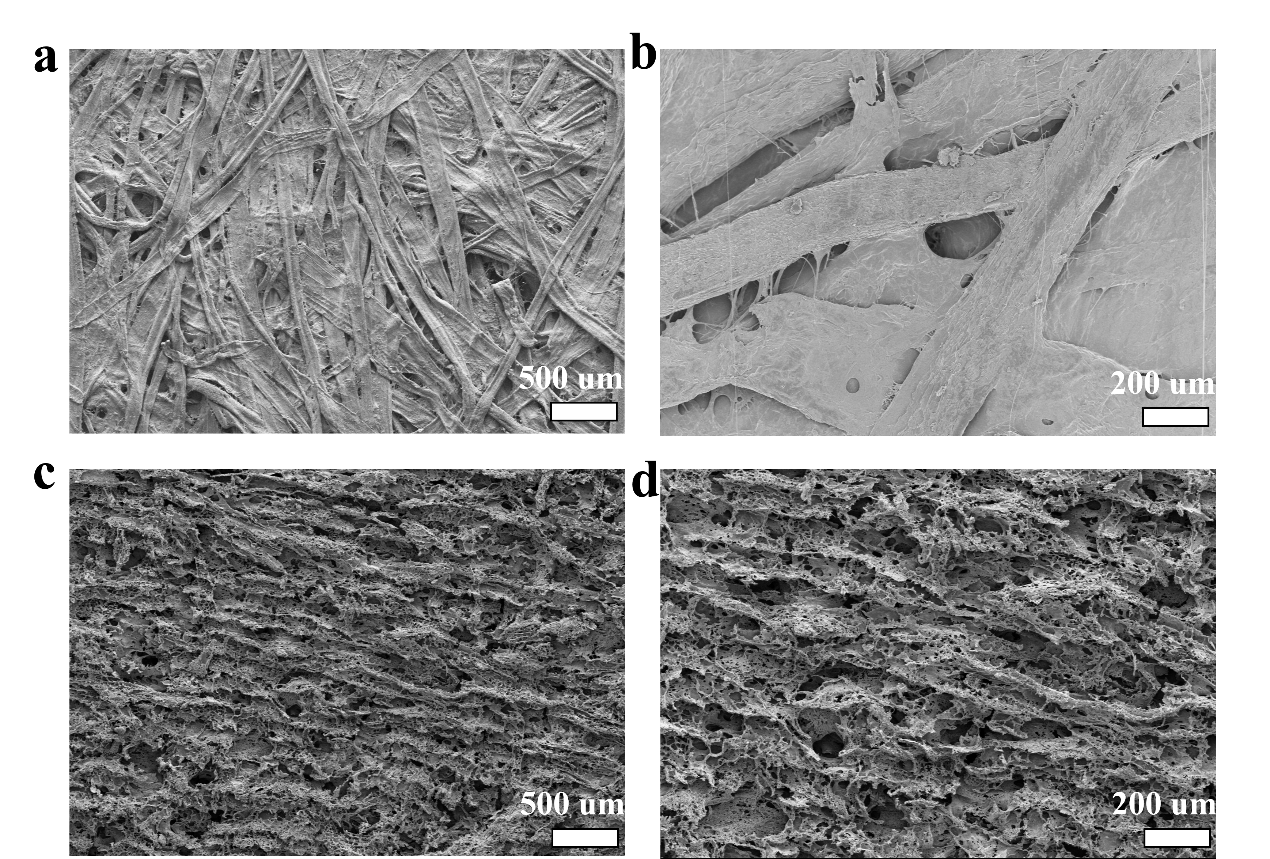


**Figure S2** SEM images of lignocellulose and LIG. a) and b) SEM images of lignocellulose; SEM images of lignocellulose-based LIG prepared with 55% laser power and 70 mm∙s^-1^ laser etching rate in c) and d).


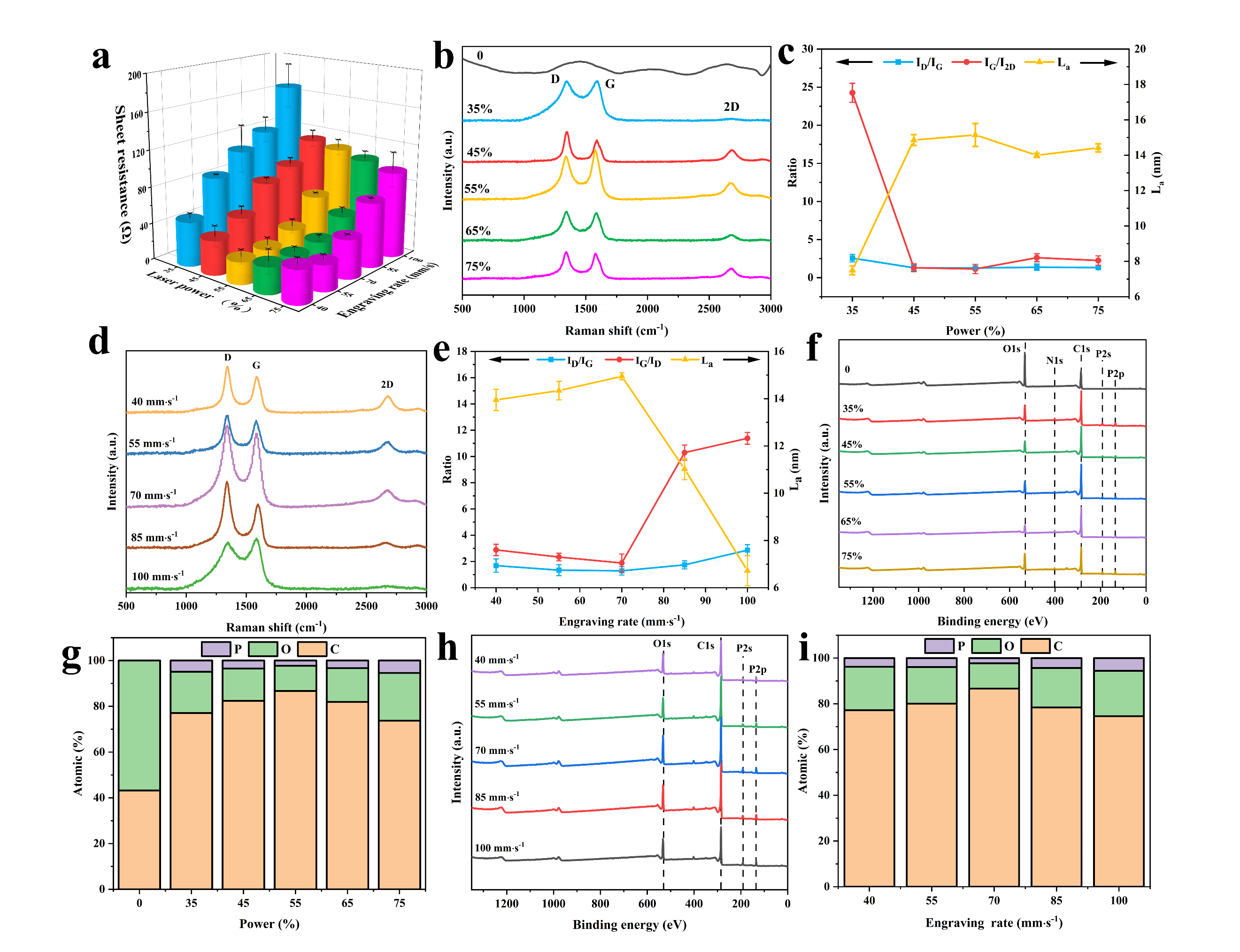


**Figure S3** Lignocellulose-based LIG laser parameter optimization characterization. a) Surface resistance of LIG b) Raman curves of LIG at a laser etching rate of 70 mm∙s^-1^ and c) variation curves of I_D_/I_G_, I_G_/I_2D_ and L_a_; d) Raman curves of LIG at 55% laser power and e) variation curves of I_D_/I_G_, I_G_/I_2D_ and L_a_; f) XPS data and g) element content of LIG at a laser etching rate of 70 mm∙s^-1^; h) XPS data and i) element content of LIG at 55% laser power.


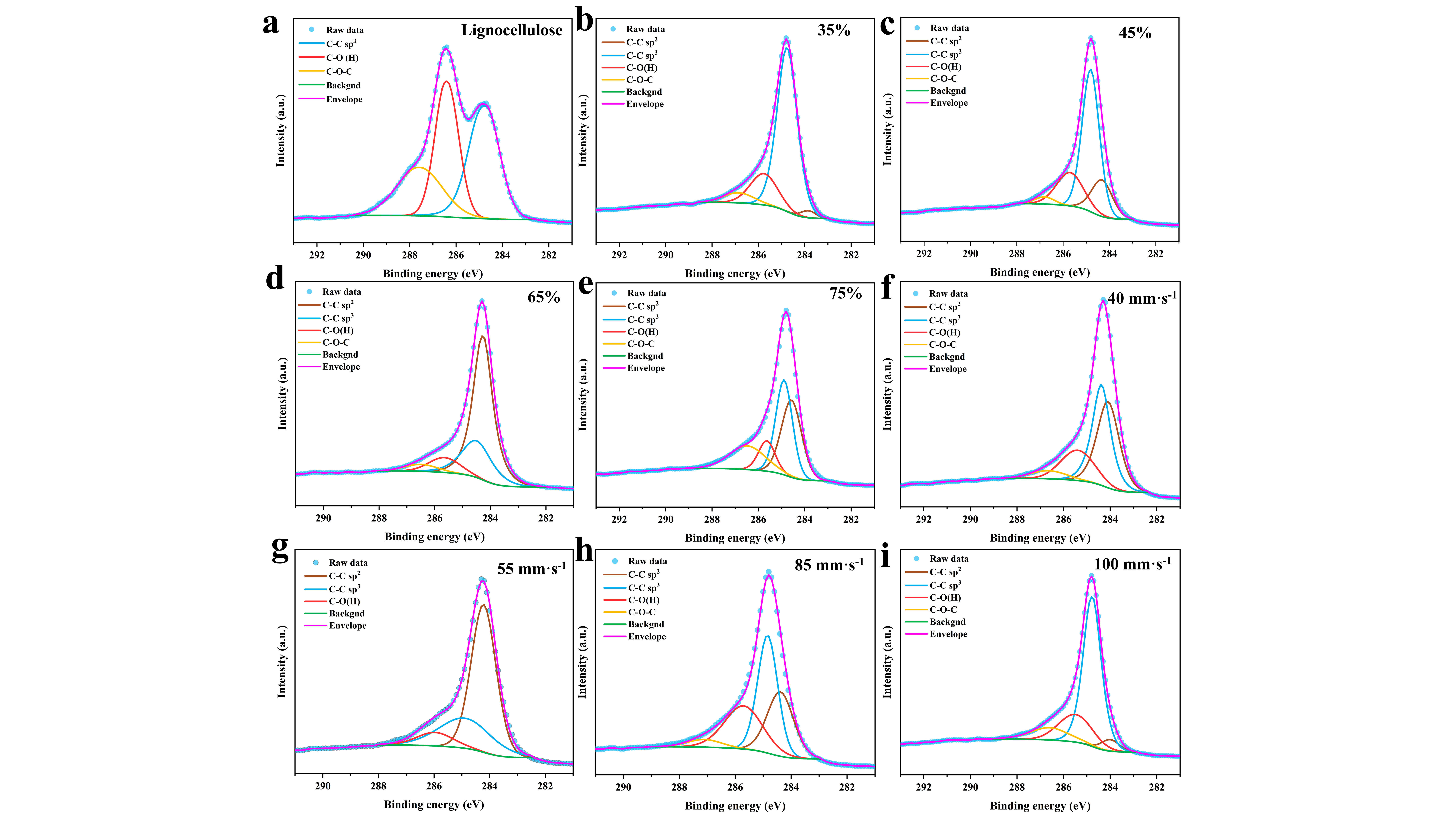


**Figure S4.** C1s spectra of lignocellulose-based LIG under different laser parameters. a) Lignocellulose b) 35% laser power; c) 45% laser power; d) 65% laser power; e) 75% laser power; (f) Laser etching rate of 40 mm∙s^-1^; g) 55 mm∙s^-1^ laser etching rate; h) Laser etching rate of 85 mm∙s^-1^; i) 100 mm∙s^-1^ laser etching rate.


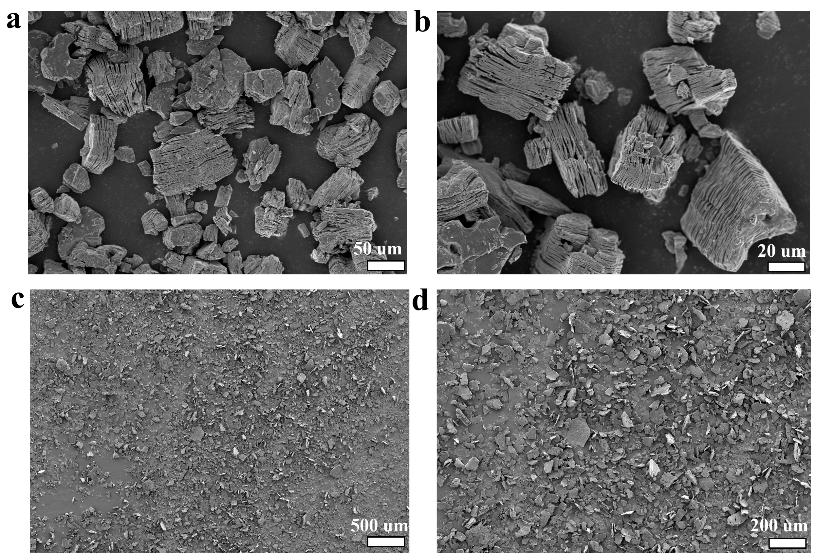


**Figure S5.** SEM images of MXene before and after exfoliation. a) and b) are the SEM images of MXene before exfoliation; c) and d) are the SEM images of the exfoliated MXene.


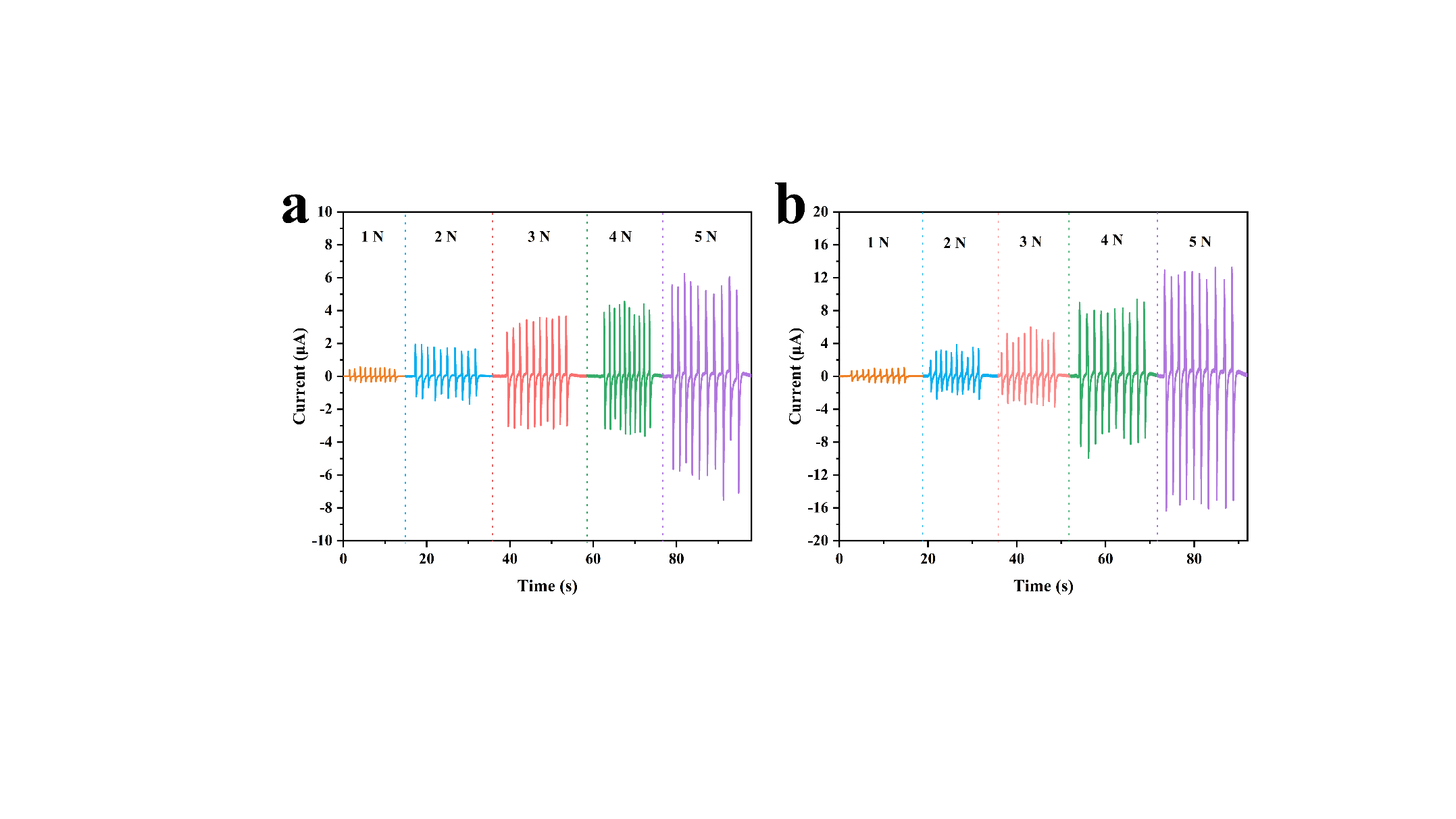


**Figure S6.** Current of the TENG on the applied force.a) LIG; b) LIG@MXene.


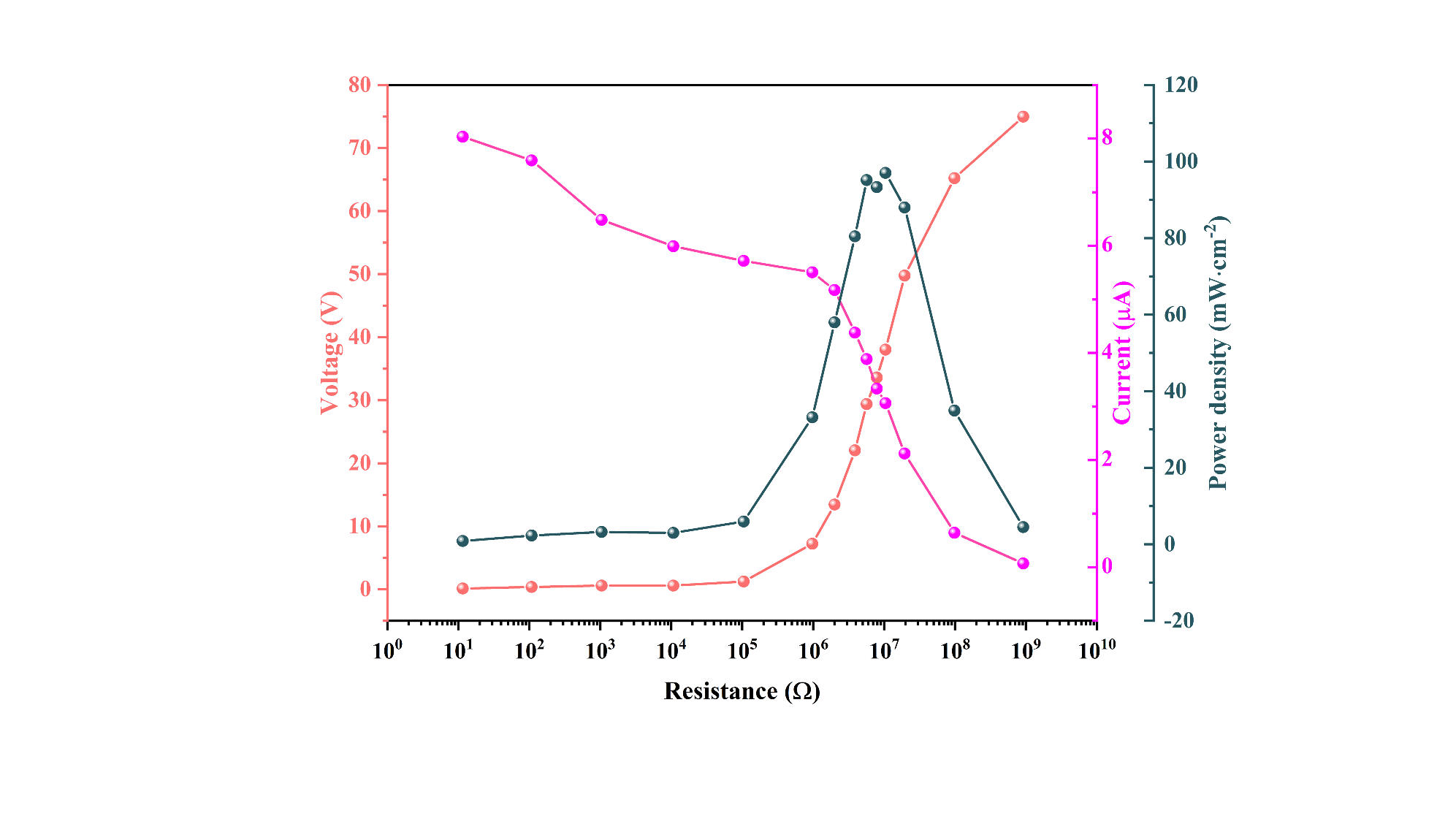


**Figure S7.** Output voltage, current and Instantaneous output power density with a vertical force of 4 N as a function of the load resistance.


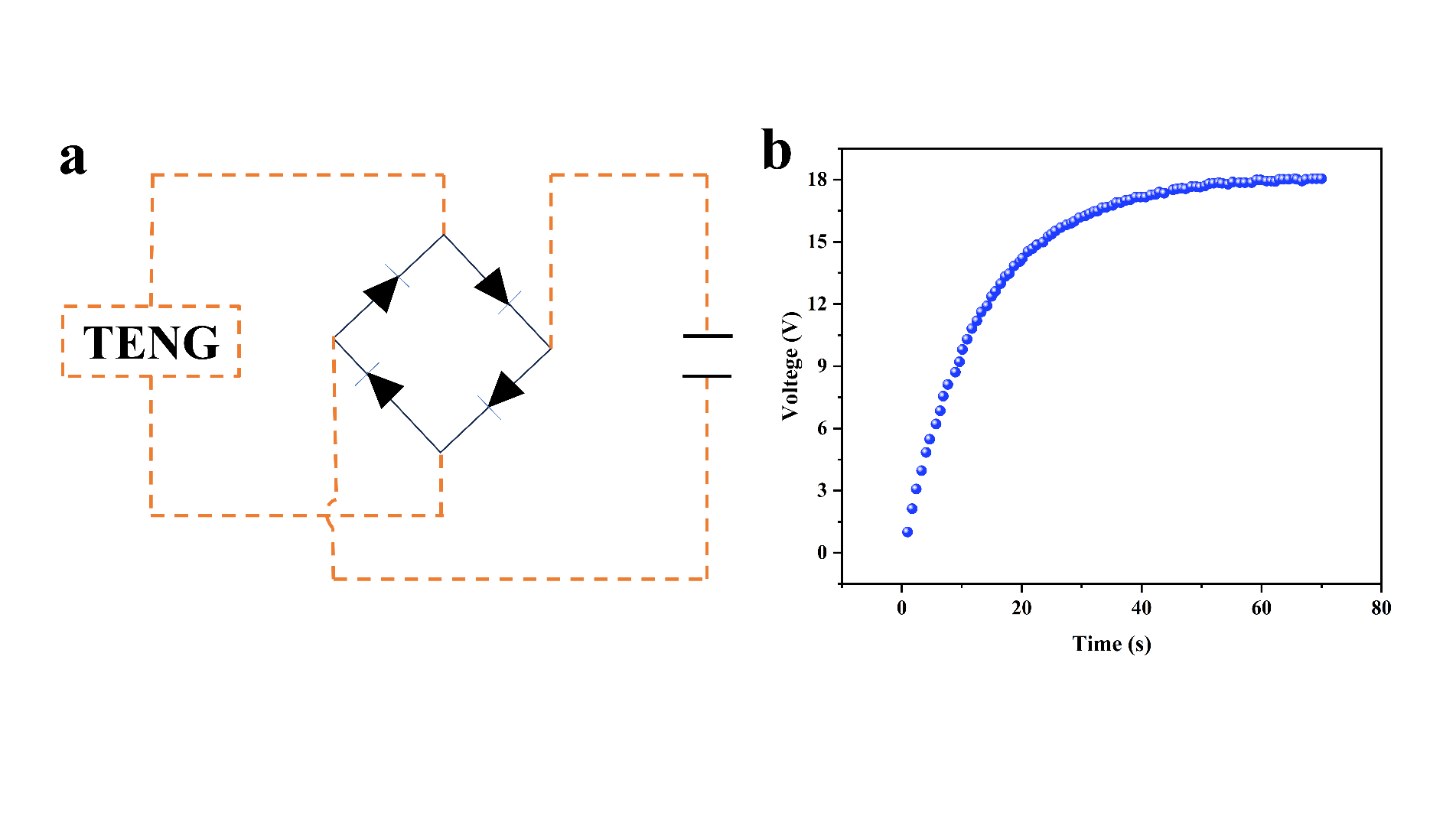


**Figure S8.** Charging curve of a 25 μF capacitor by the TENG through a bridge rectifier. a) charging circuit diagram; b) the curve of capacitor voltage versus charging time.


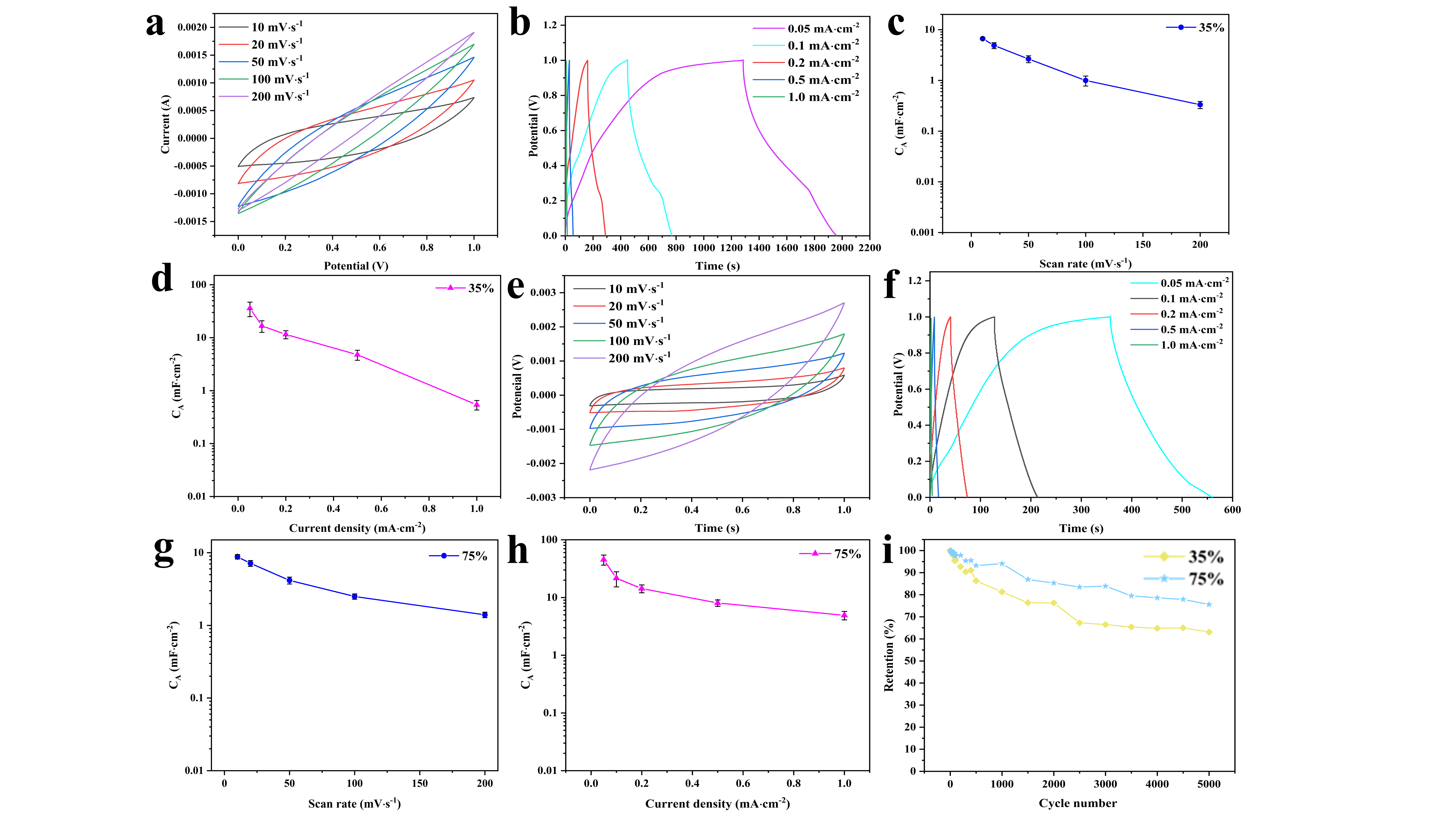


**Figure S9.** Performance testing of lignocellulose-based LIG supercapacitors at different laser power. a) CV curves and b) GCD curves of LIG supercapacitors at 35% laser power; c) C_A_ at different scan rates and d) C_A_ at different current densities for LIG supercapacitors at 35% laser power; e) CV curves and f) GCD curves of LIG supercapacitors at 75% laser power; g) C_A_ at different scan rates and h) C_A_ at different current densities for supercapacitors at 75% laser power; i) C_A_ retention rate after 5,000 charge-discharge cycles of LIG supercapacitors at 0.1 mA∙cm^-2^ current density.

**
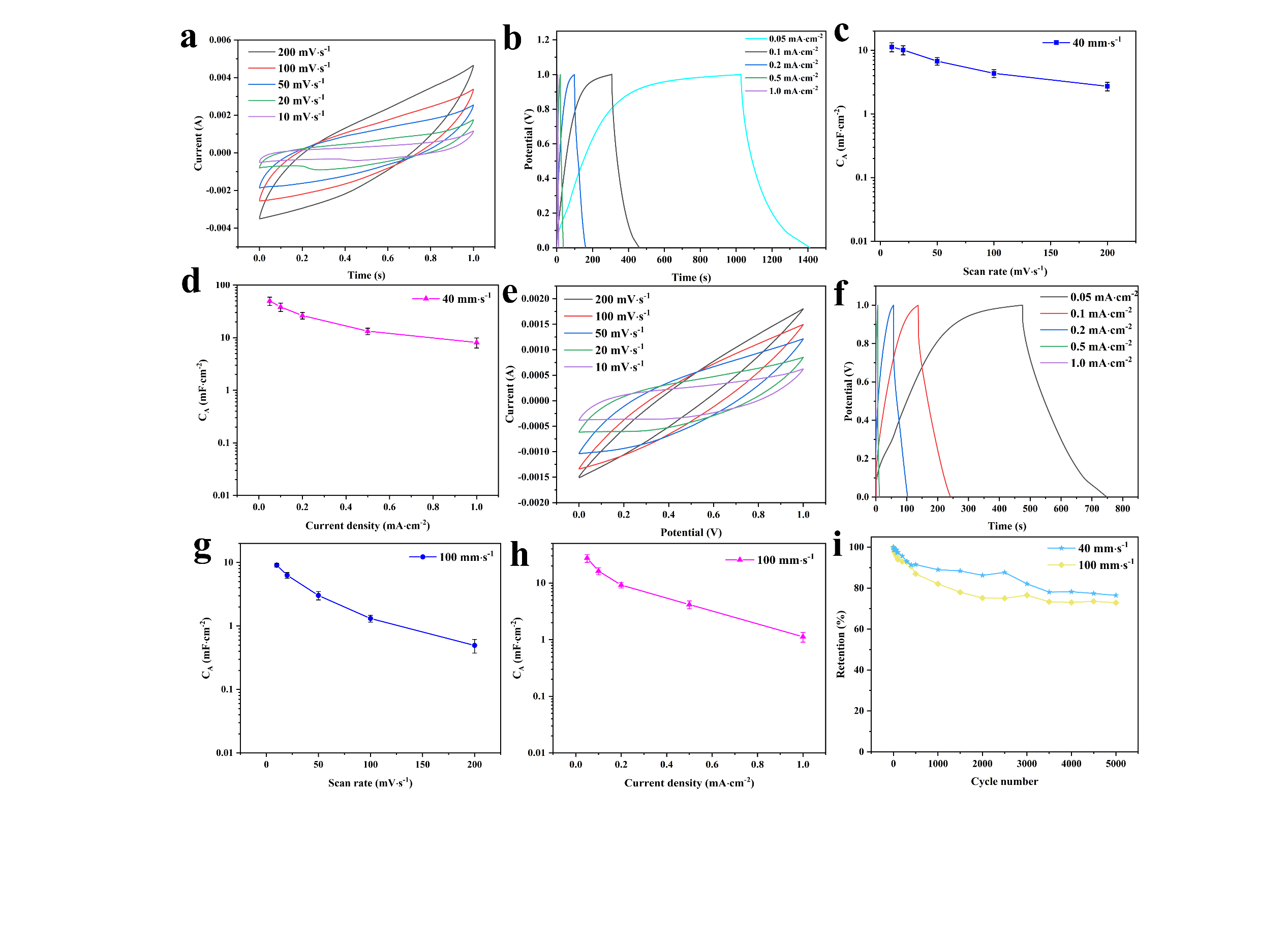
**

**Figure S10.** Performance tests of lignocellulose-based LIG supercapacitors at different etching rates. a) CV test curve of LIG supercapacitor at a laser etching rate of 40 mm∙s^-1^ and b) GCD test curve; c) C_A_ of LIG supercapacitors at different scanning rates at a laser etching rate of 40 mm∙s^-1^ and d) C_A_ at different current densities; e) CV test curve of LIG supercapacitor at a laser etching rate of 100 mm∙s^-1^ and f) GCD test curve; g) C_A_ of LIG supercapacitors at different scanning rates at a laser etching rate of 100 mm∙s^-1^ and h) C_A_ at different current densities; i) C_A_ retention rate of LIG supercapacitors after 5000 cycles of charge and discharge at a current density of 0.1 mA∙cm^-2^.


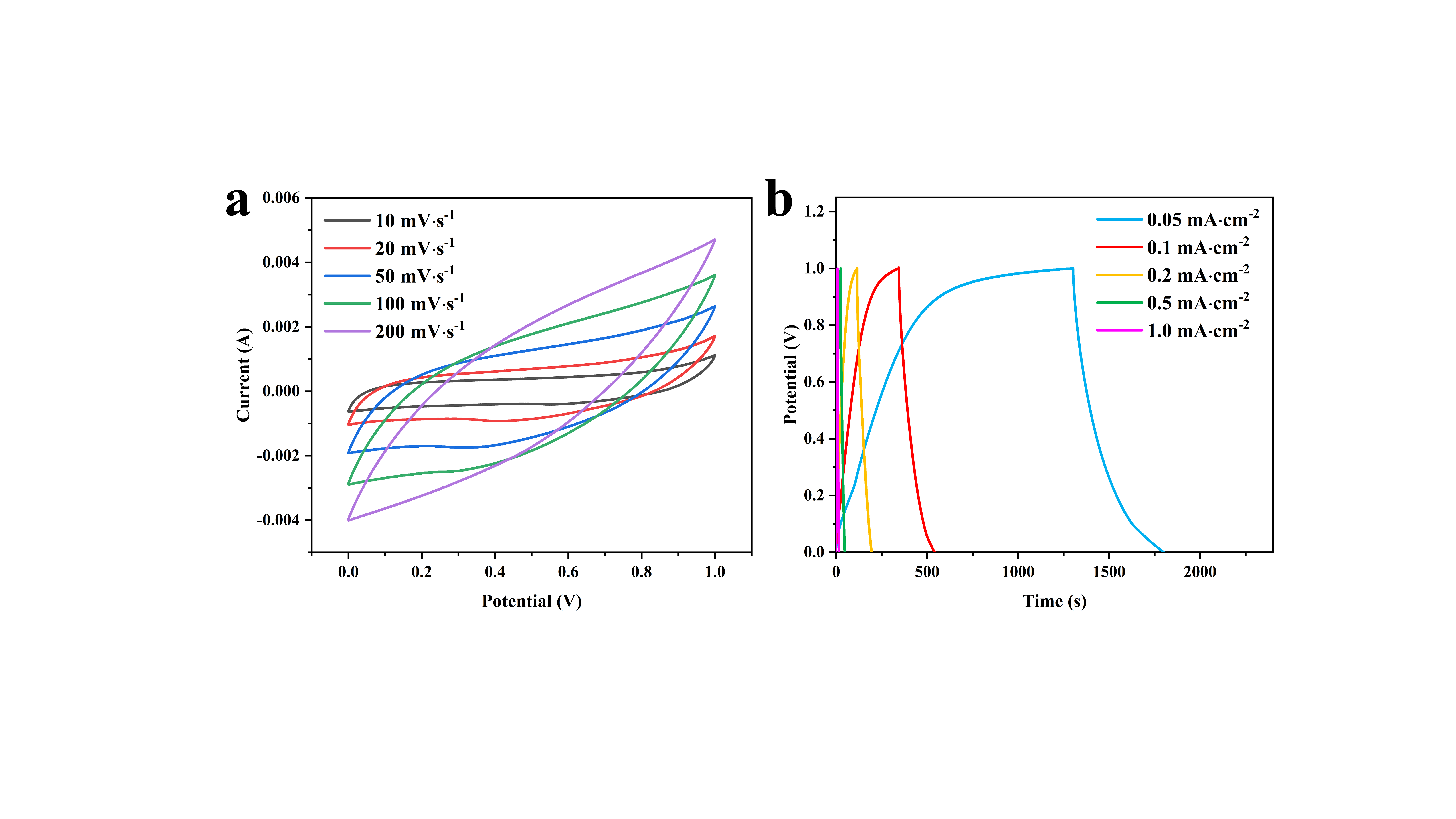


**Figure S11.** Performance test of LIG supercapacitors fabricated at 55% laser power and a laser etching rate of 70 mm∙s^-1^. a) CV; b) GCD.


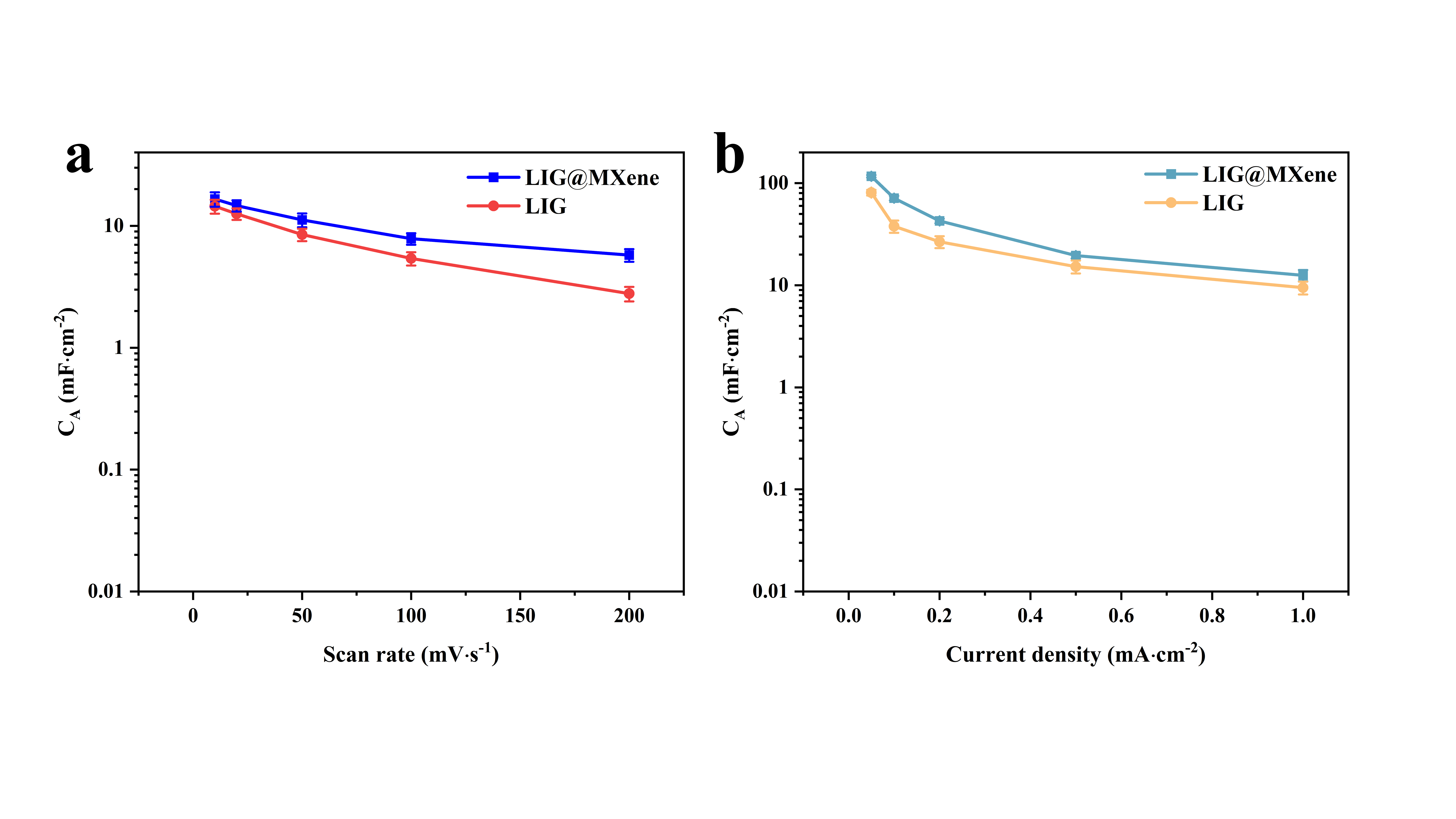


**Figure S12.** The C_A_ of C_A_ of LIG@MXene and LIG supercapacitors. a) C_A_ at different scanning rates; b) C_A_ at different current densities.


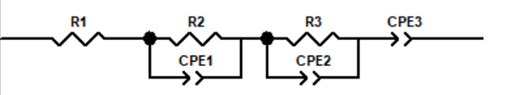


**Figure S13.** Equivalent circuit used to fit EIS spectra.


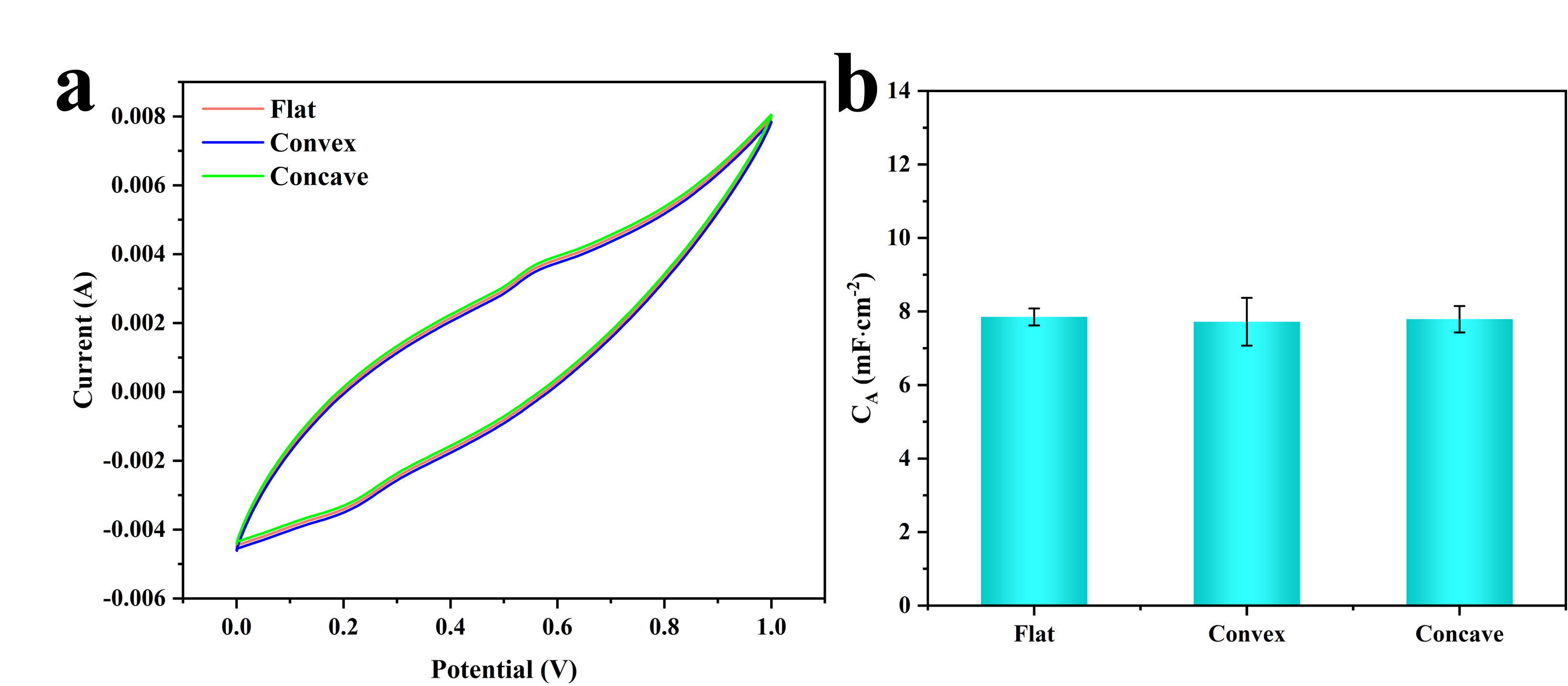


**Figure S14.** Performance of LIG@Mxene supercapacitors under 15° bending. a) CV curve; b) C_A_.


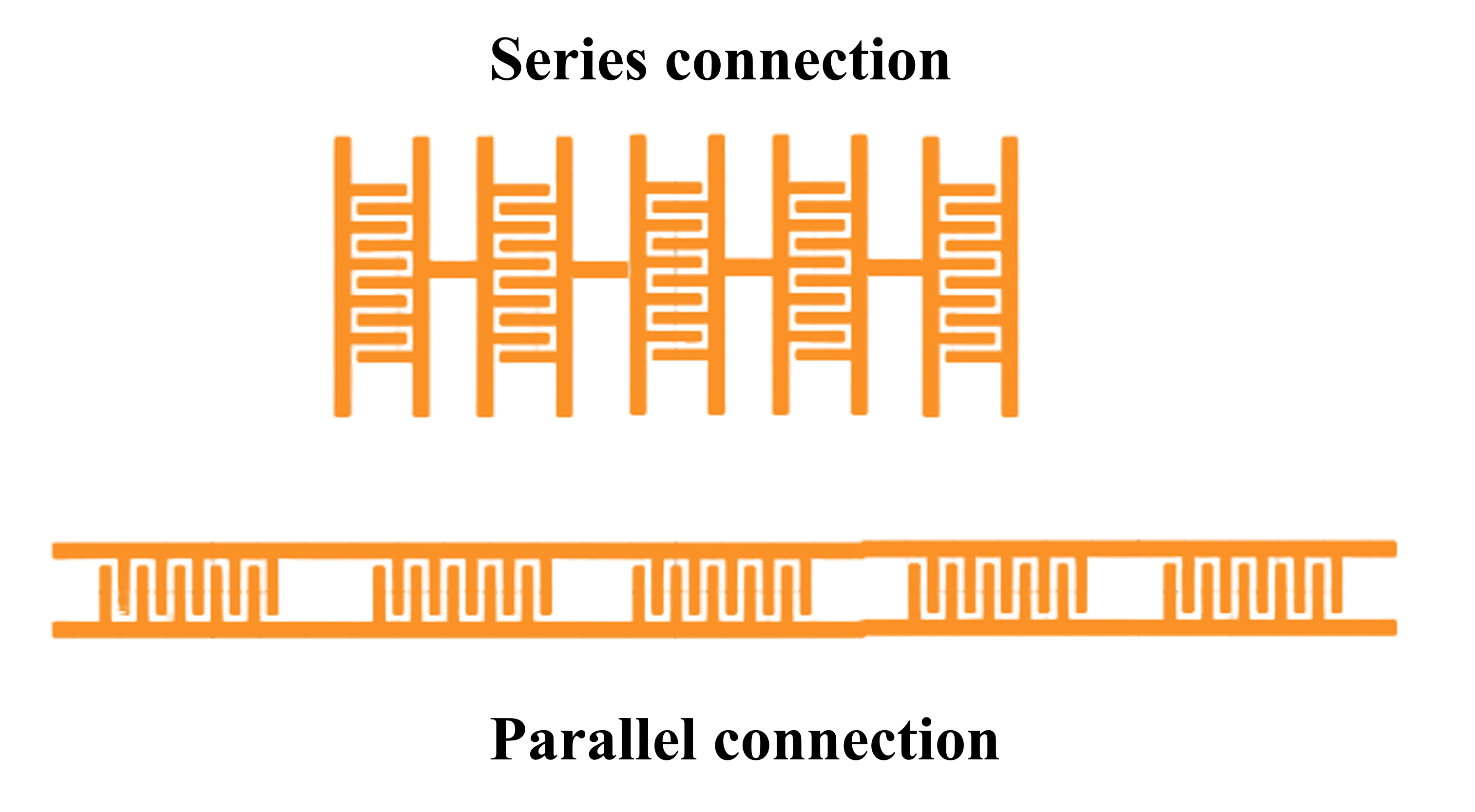


**Figure S15.** Schematic diagrams of series and parallel connection of supercapacitors.


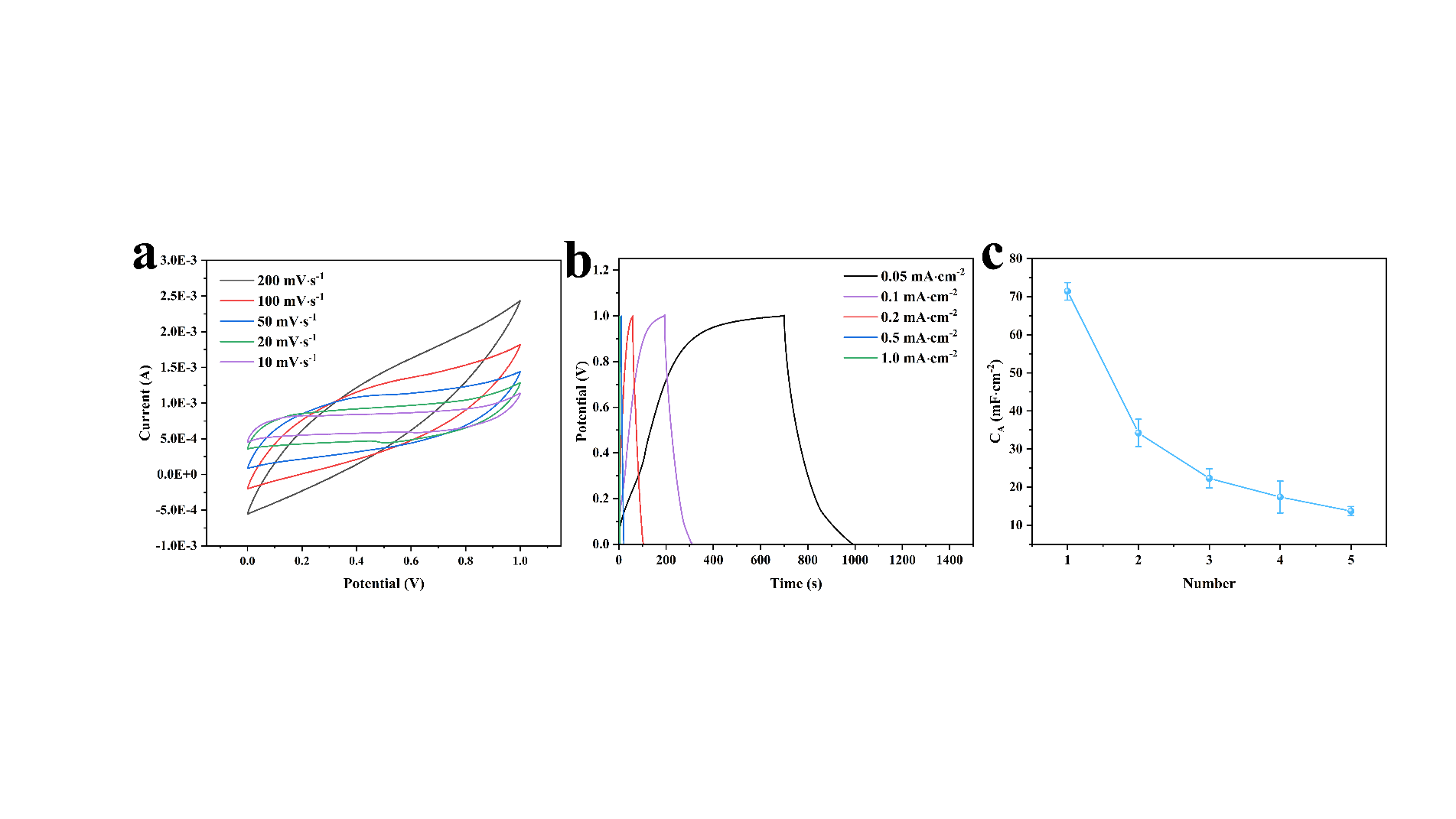


**Figure S16.** Performance of LIG@MXene based supercapacitors in series. a) CV curves of five supercapacitors connected in series and the b) GCD curves; c) the relationship between the capacitance of a supercapacitor and the number of series connections at a current density of 0.1 ma∙cm^-2^.


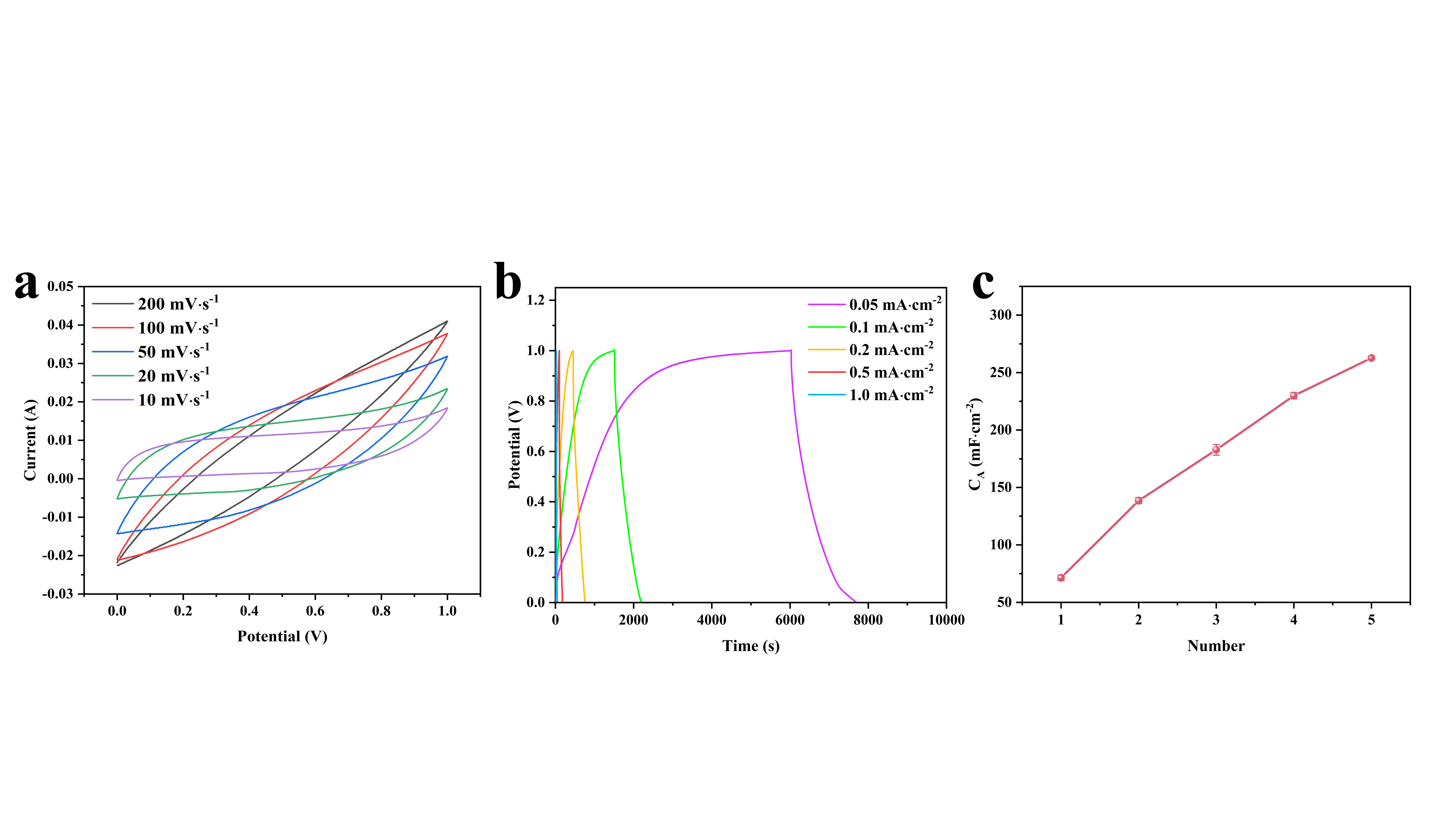


**Figure S17.** Performance of parallel supercapacitors based on LIG@MXene. a) CV curves of five supercapacitors in parallel and b) GCD curves; c) the relationship between the capacitance of a supercapacitor and the number of parallel connections at a current density of 0.1 ma∙cm^-2^.


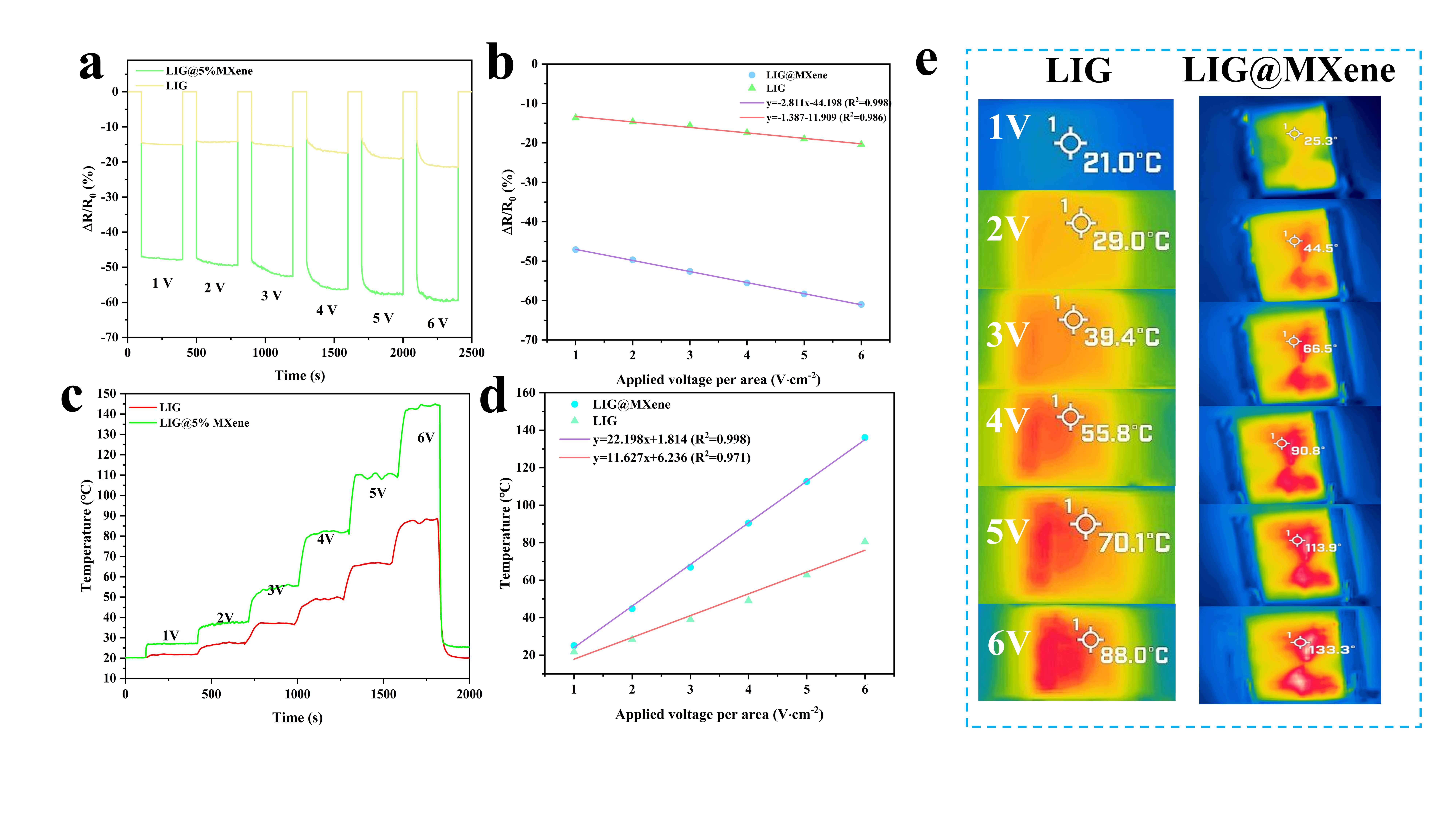


**Figure S18.** Performance tests of lignocellulose-based LIG and LIG@MXene joule heaters. a) ΔR/R_0_ of the Joule heater at different voltages; b) The fitting curve of ΔR/R_0_ and voltage; c) Temperature response of Joule heaters at different voltages; d) Fitting curve of temperature and voltage; e) Infrared camera images of the Joule heater at different voltages.


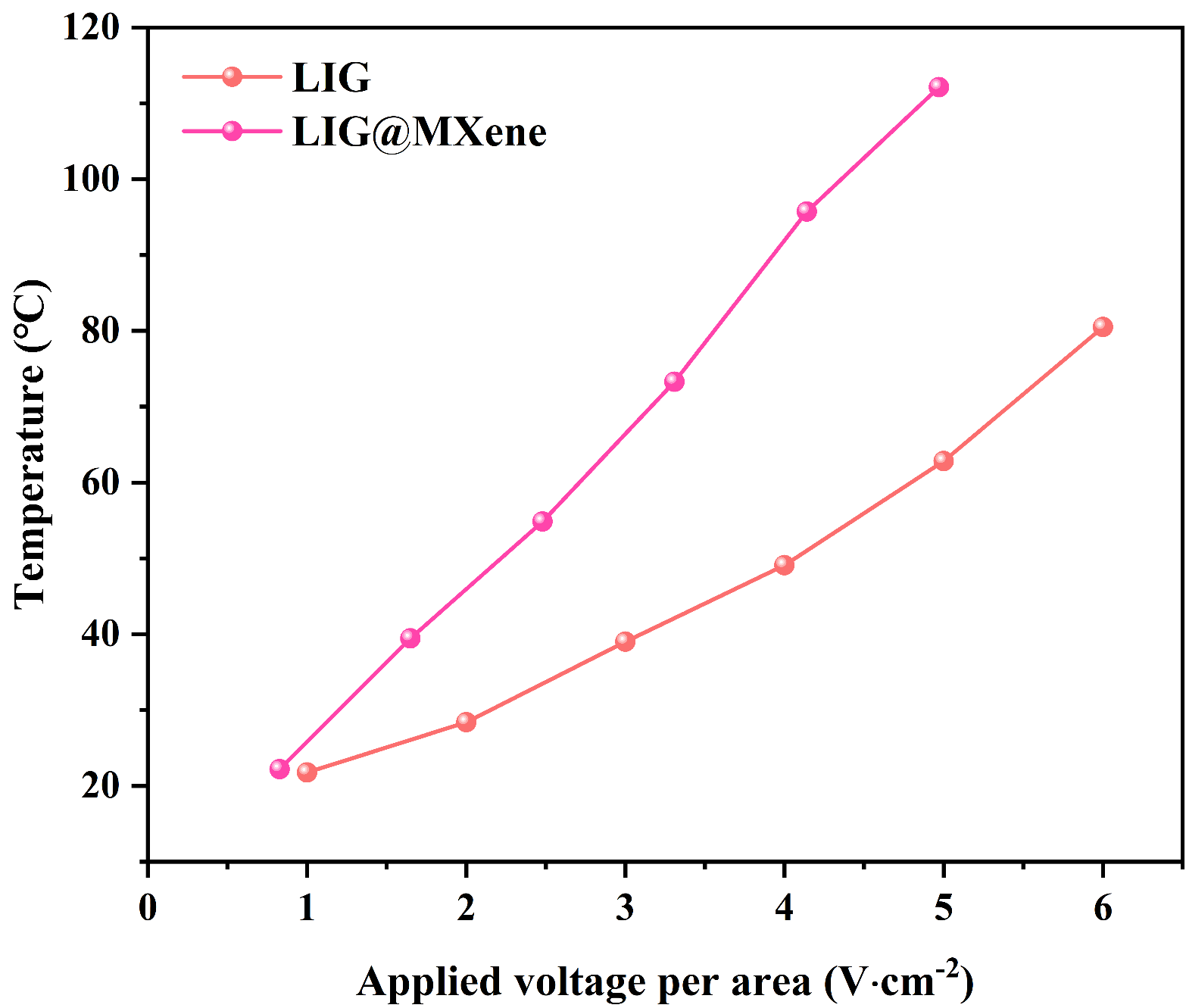


**Figure S19.** Performance Comparison of LIG and LIG@MXene Joule Heaters after Resistance Normalization.


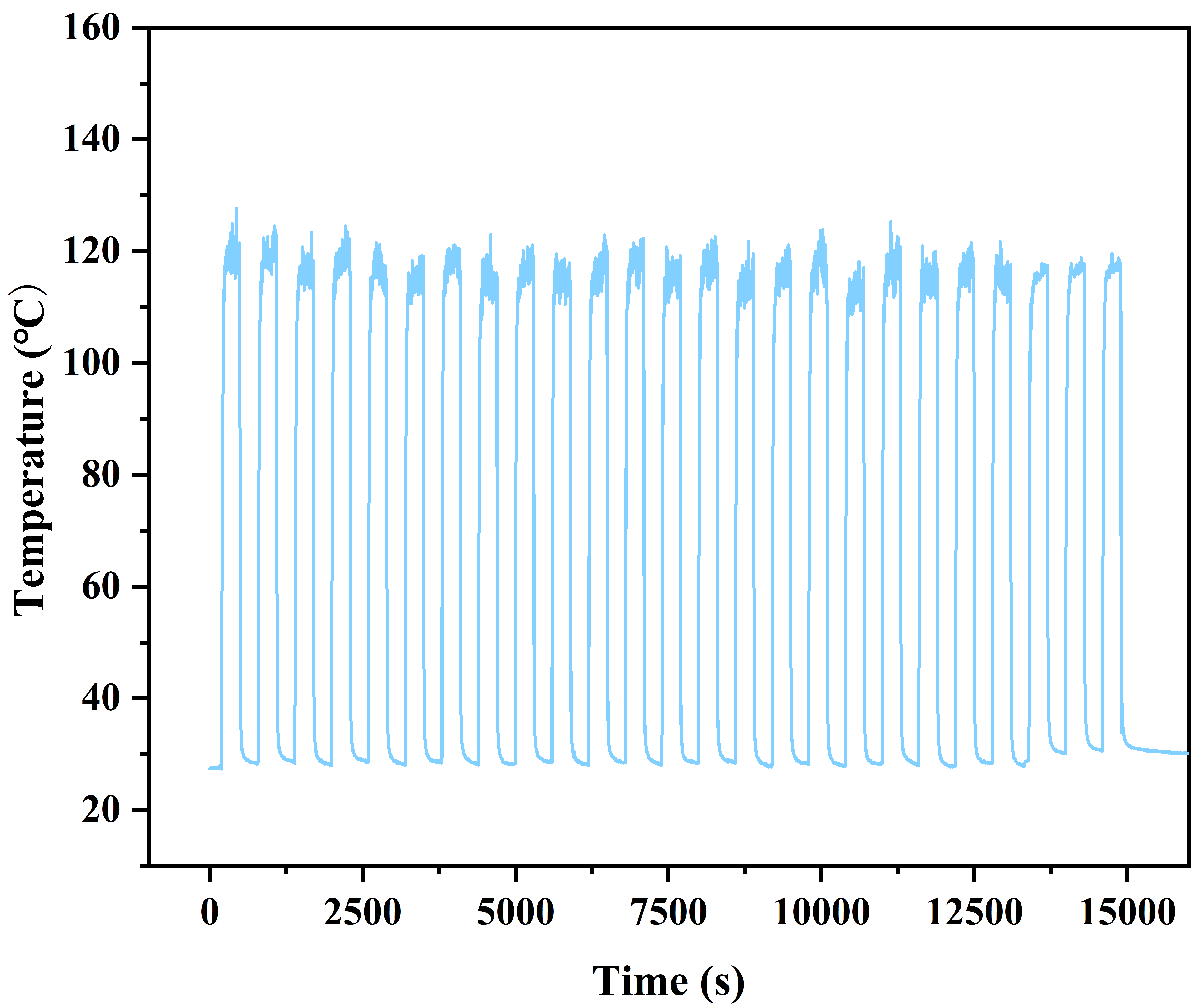


**Figure S20.** Temperature profile of the LIG@MXene Joule heater under 5 V during 25 heating cycles (each cycle consists of 300 s voltage application and 300 s cooling at room temperature).


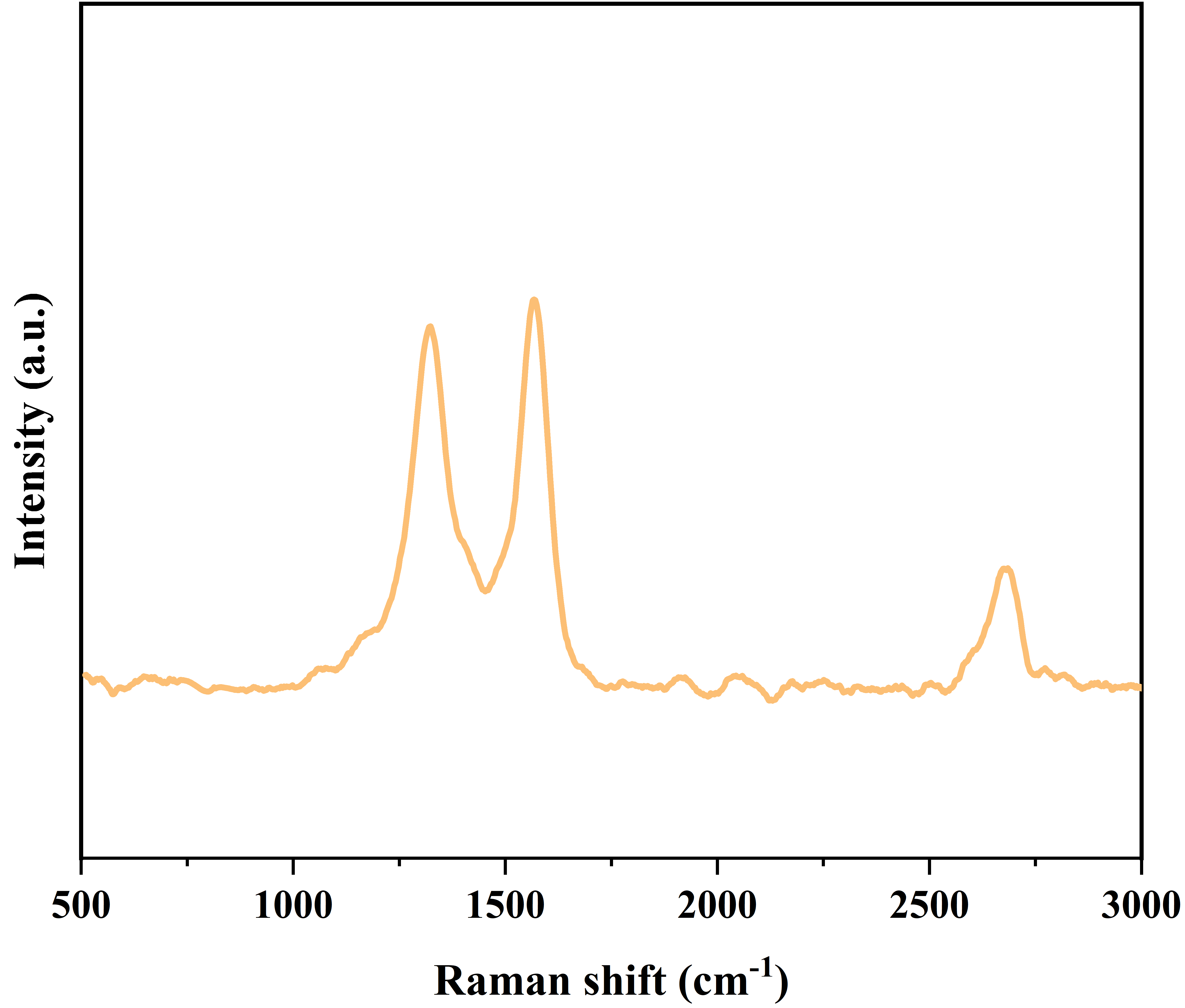


**Figure S21.** Raman spectra of the LIG@MXene Joule heater under 5 V after 25 heating cycles.


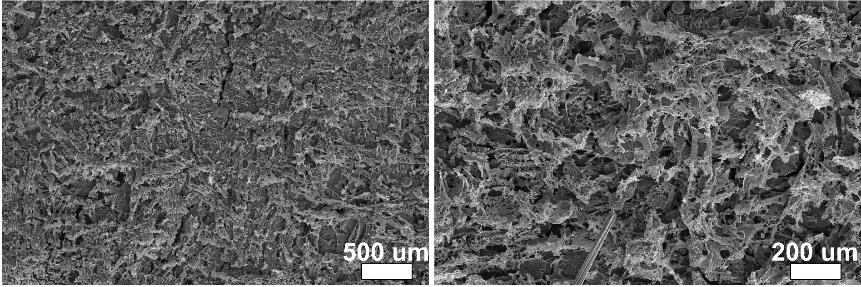


**Figure S22.** SEM image of the LIG@MXene Joule heater after 25 heating cycles at an applied voltage of 5 V.


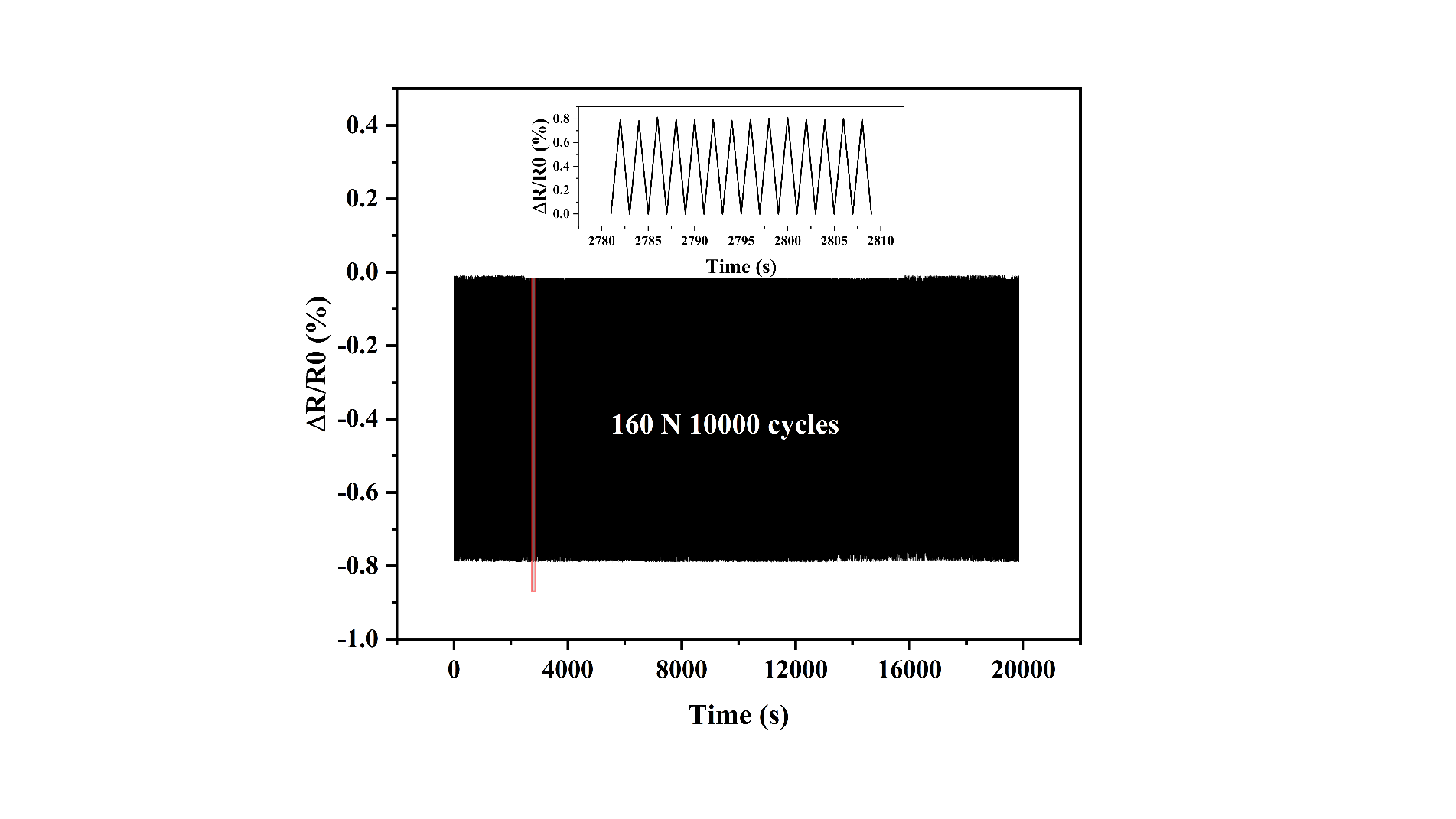


**Figure S23.** Cycle-dependent pressure response of the LIG@MXene pressure sensor over 10,000 loading–unloading cycles under a constant 160 N force (inset: enlarged view of a local segment).


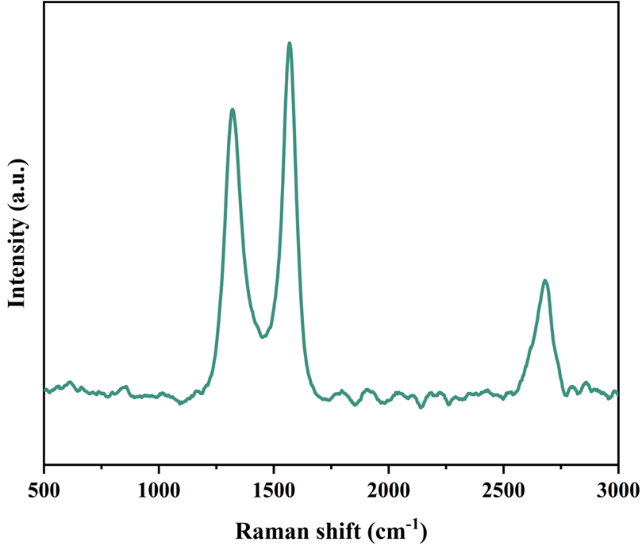


**Figure S24.** Raman spectra of the LIG@MXene pressure sensor after 10,000 tests at 160N pressure.


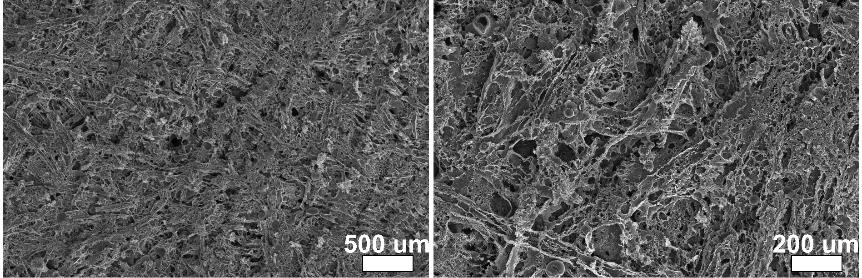


**Figure S25.** SEM image of the LIG@MXene pressure sensor after 10,000 tests at 160N pressure.


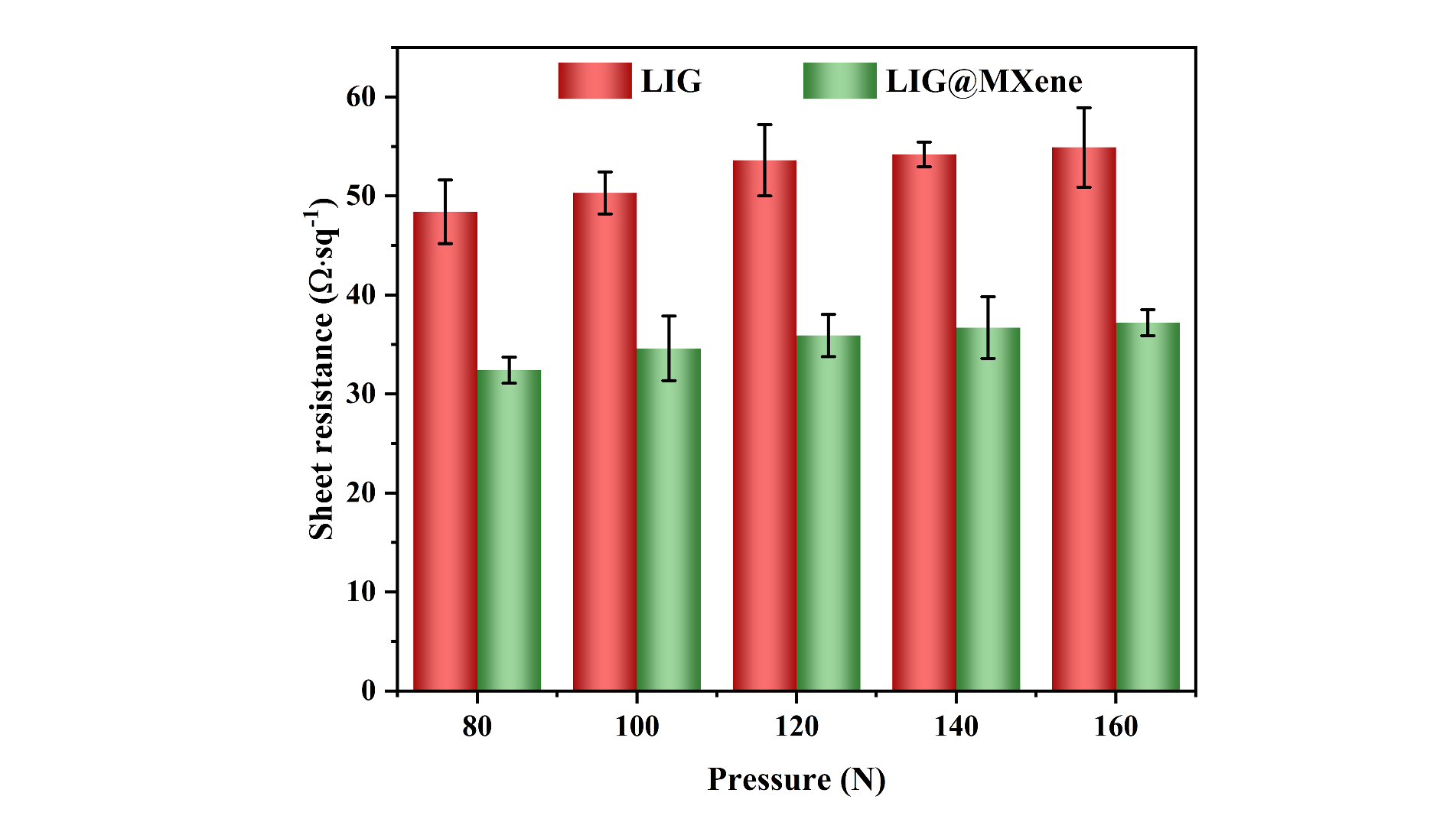


**Figure S26.** Sheet resistance of the LIG@MXene pressure sensor after testing at different pressures.


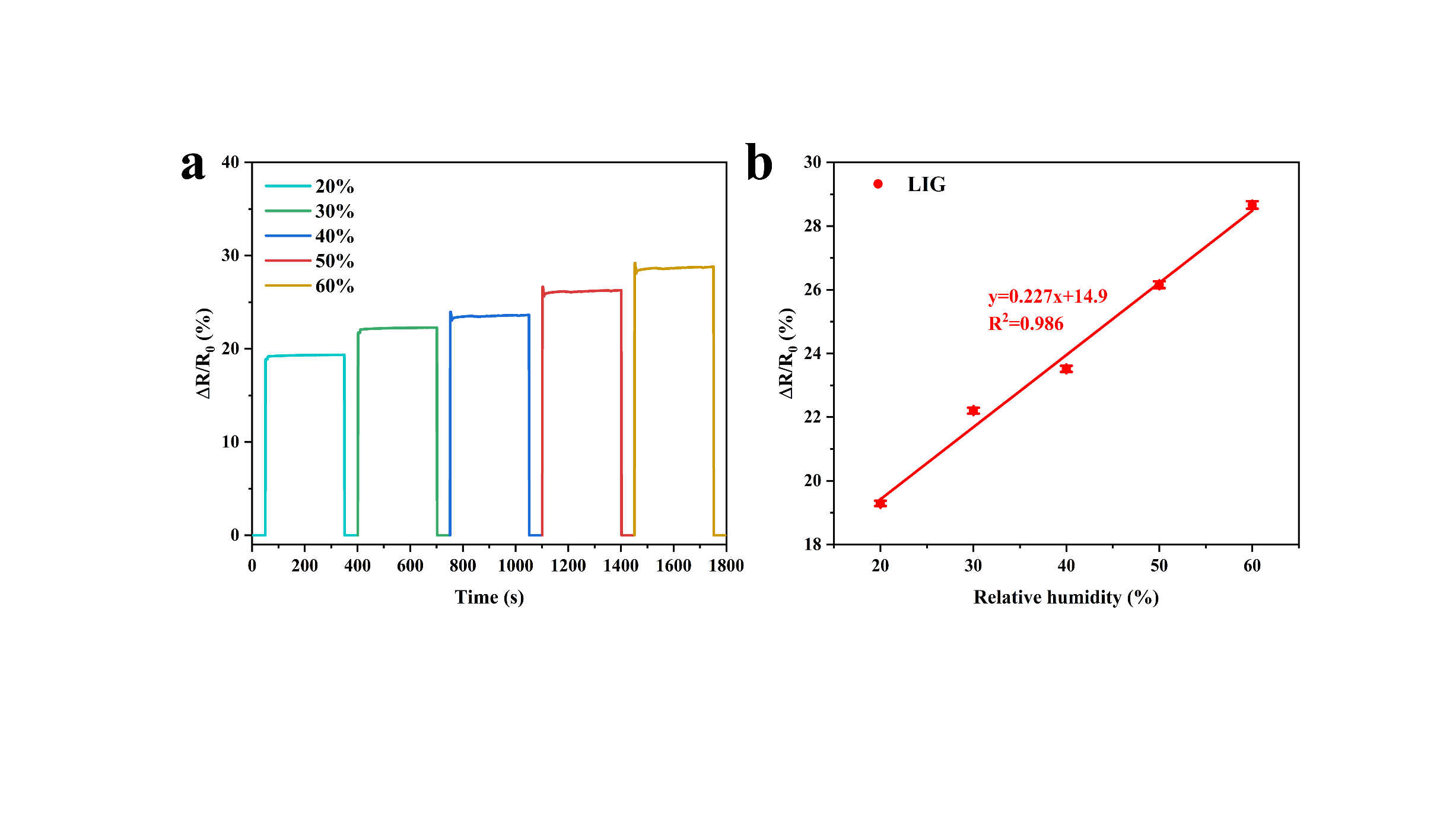


**Figure S27.** Performance testing of humidity sensors. a) Sensor resistance response at different humidity levels and b) fitting curv


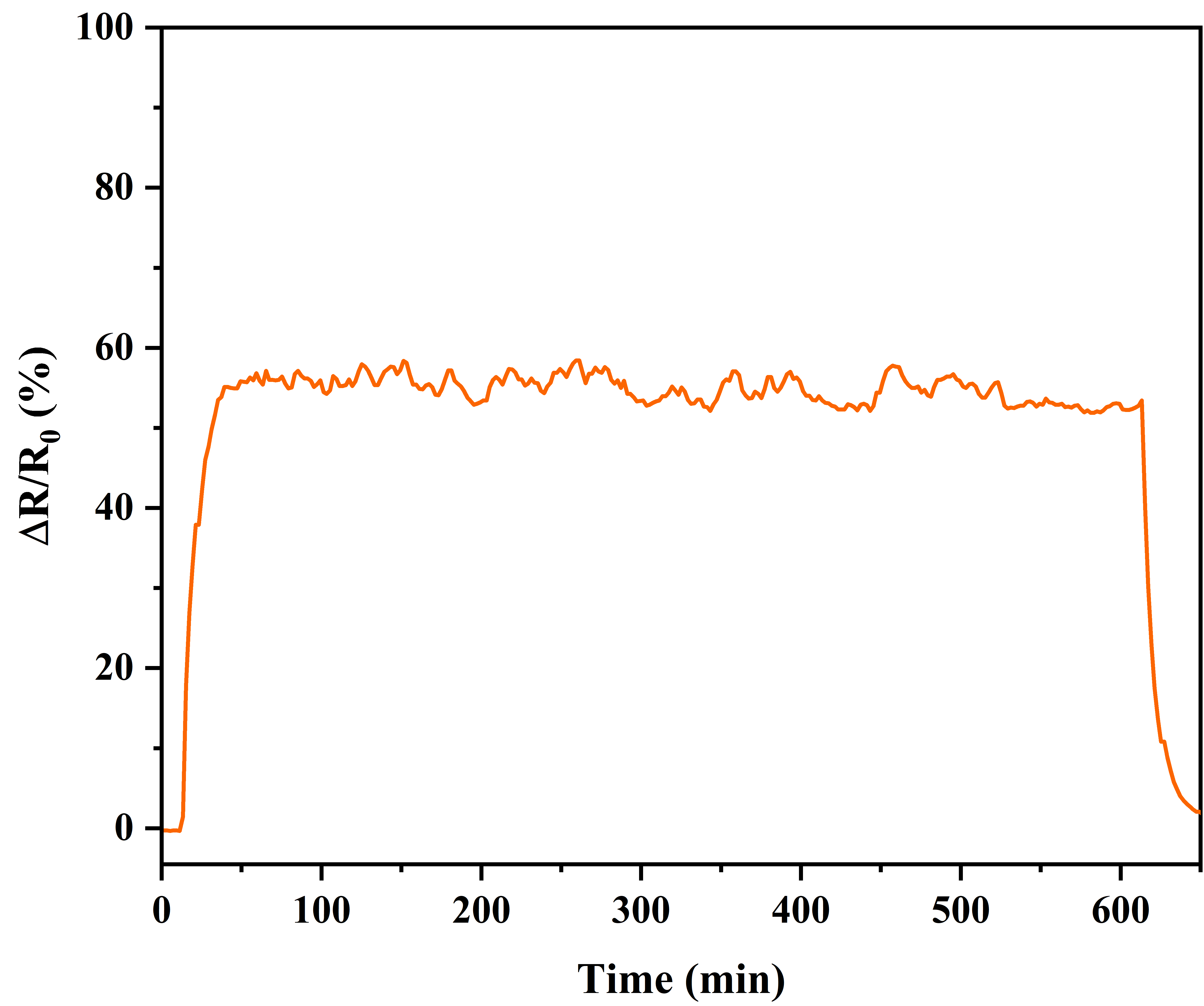


**Figure S28.** The resistance change of the LIG@MXene humidity sensor was tested at 40%RH for 10 hours.


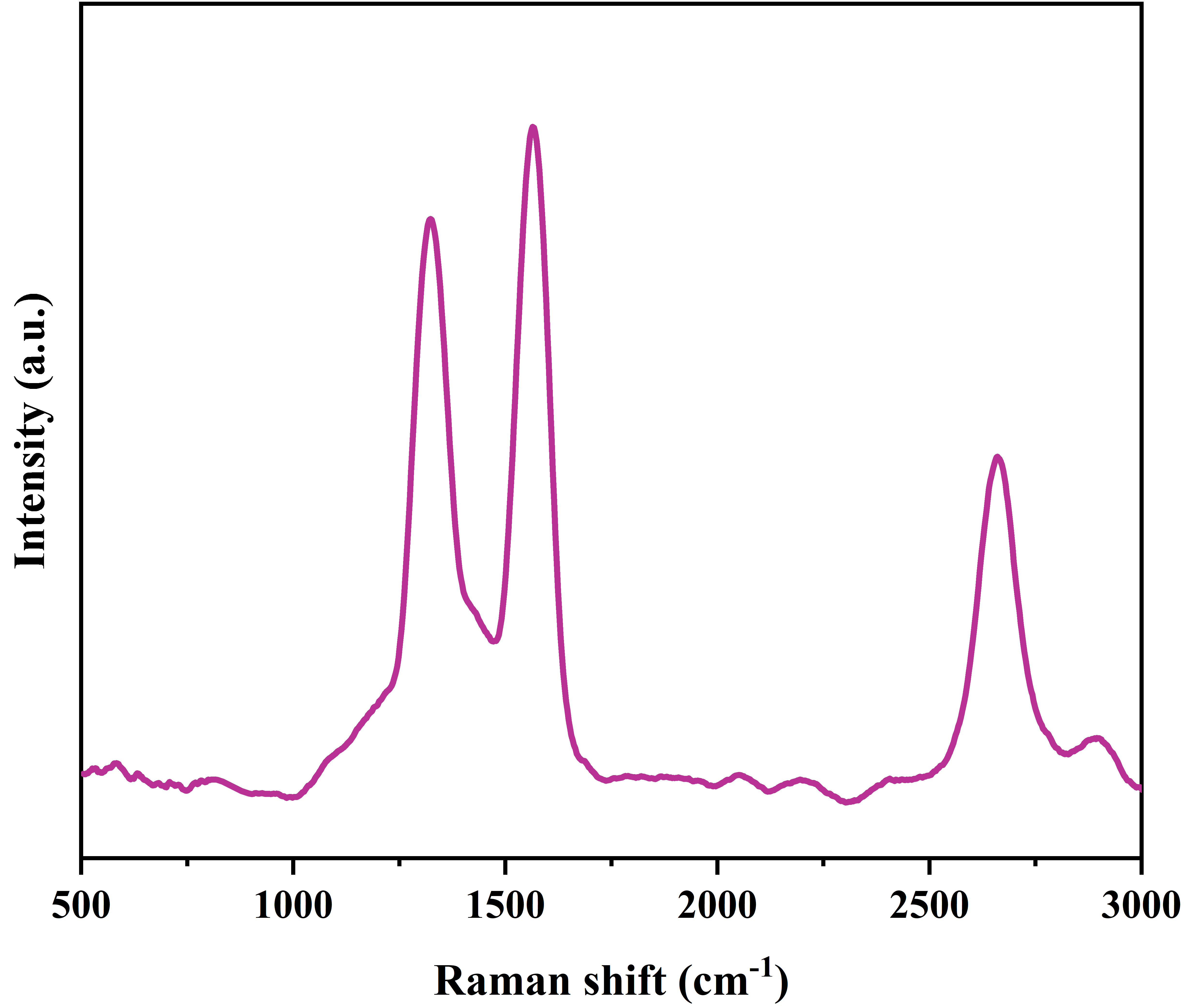


**Figure S29.** Raman spectra of the LIG@MXene humidity sensor after 10 hours of testing at 40%RH.


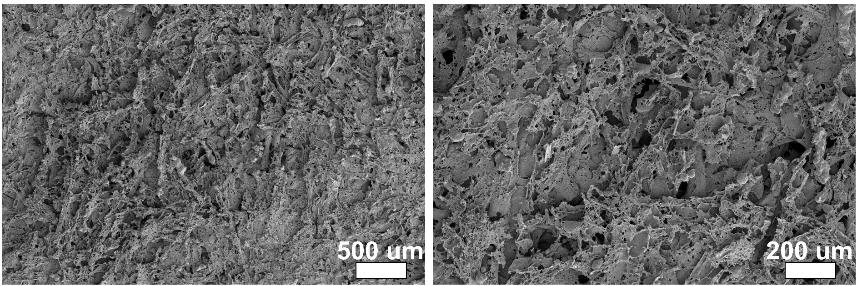


**Figure S30.** SEM image of the LIG@MXene humidity sensor after 10 hours of testing at 40%RH.


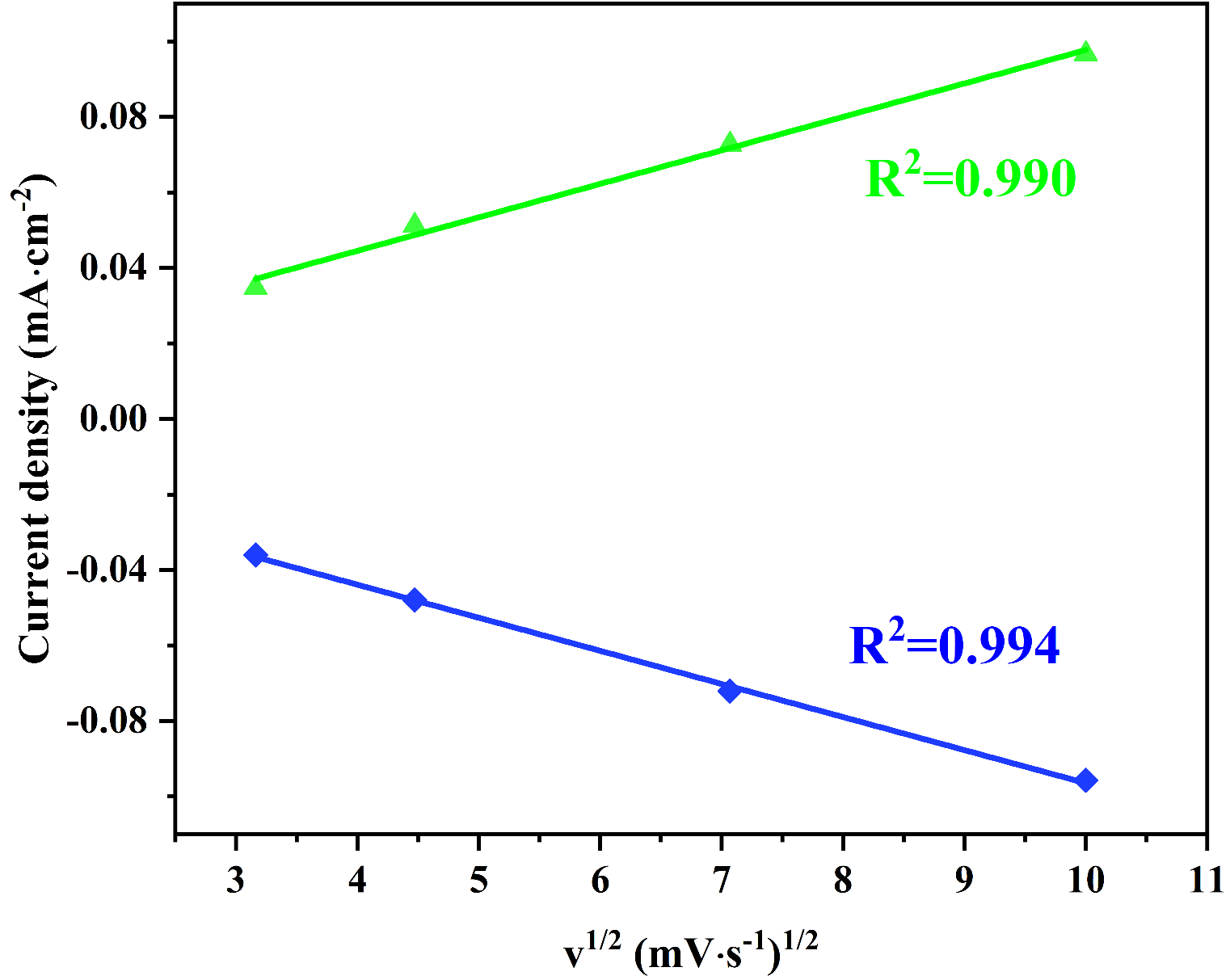


**Figure S31.** The relationship between the peak current and the square root of the scanning rate of the LIG electrochemical sensor at 5 mM K_3_[Fe(CN)_6_] and 0.1 M KCl was plotted using fitted linear regression curves.


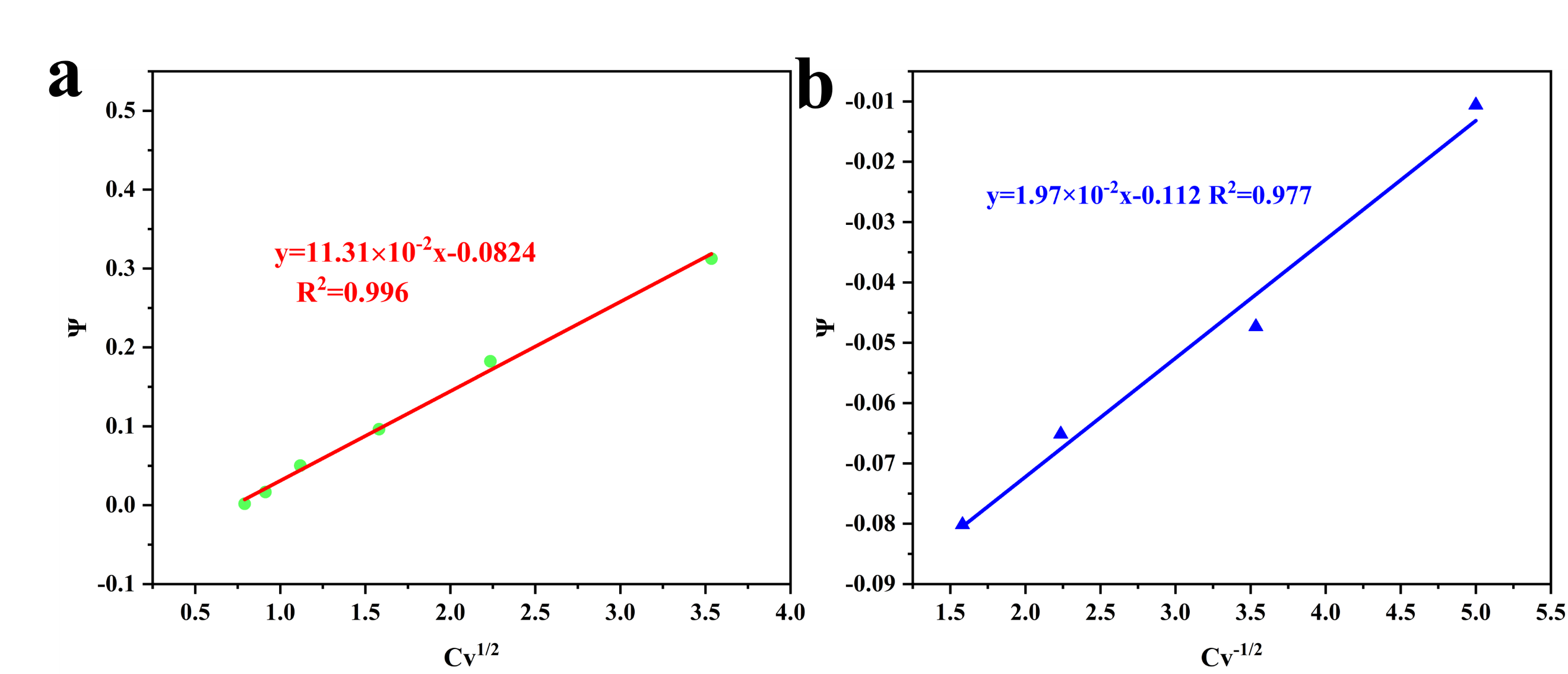


**Figure S32.** The linear fitting curve for calculating the HET constant. a) LIG@MXene; b) LIG.


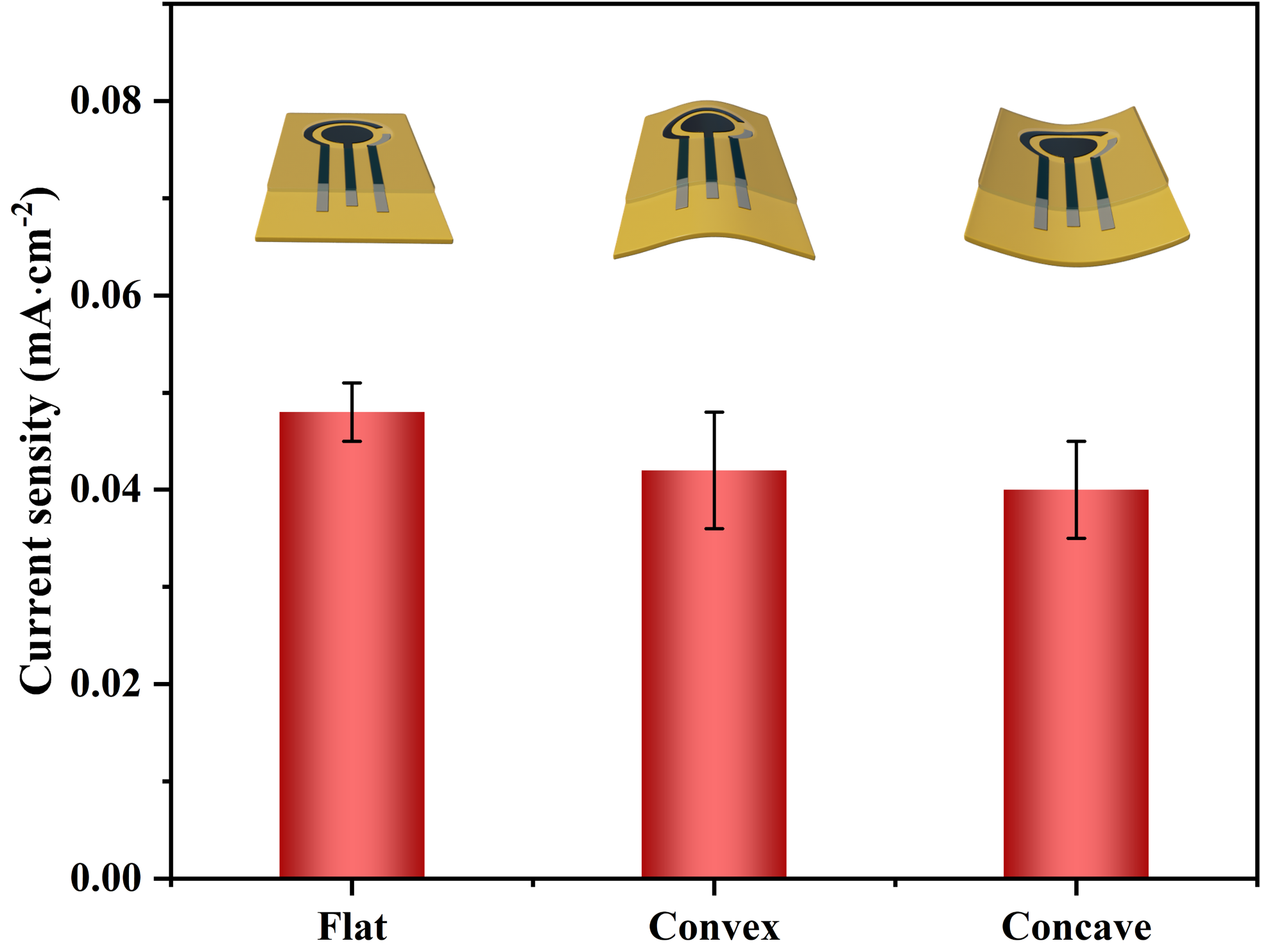


**Figure S33.** Peak currents for different bending states of the sensor in 5 mM K₃[Fe(CN)₆]/K₄[Fe(CN)₆] and 0.1 M KCl, tested at a scan rate of 10 mV∙s^-1^ with the electrode bent at 15°.


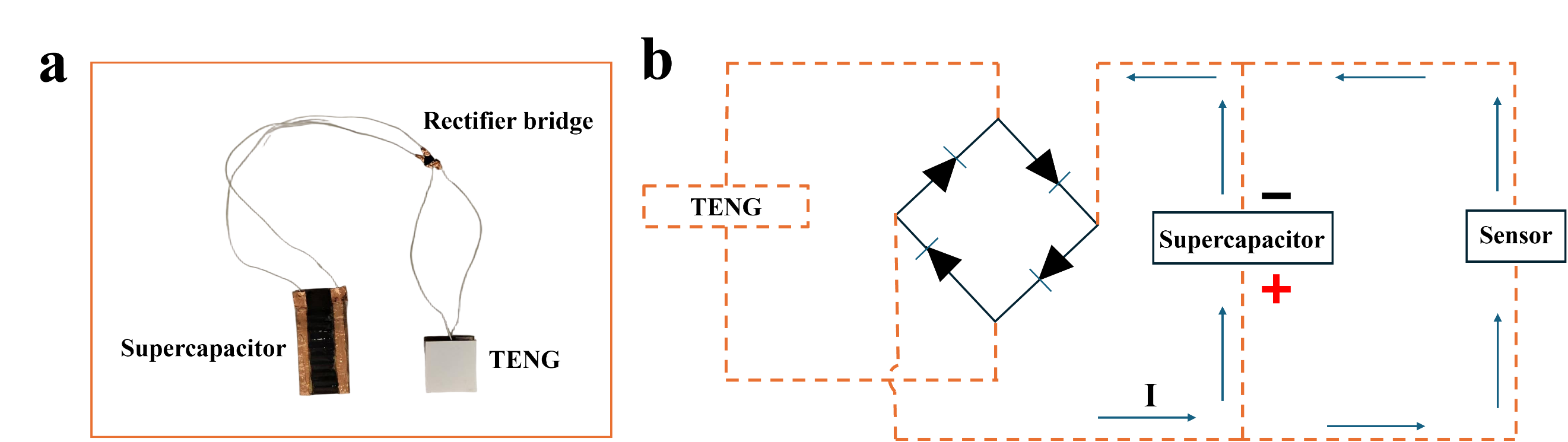


**Figure S34.** Schematic diagram of the self-powered system device.

**
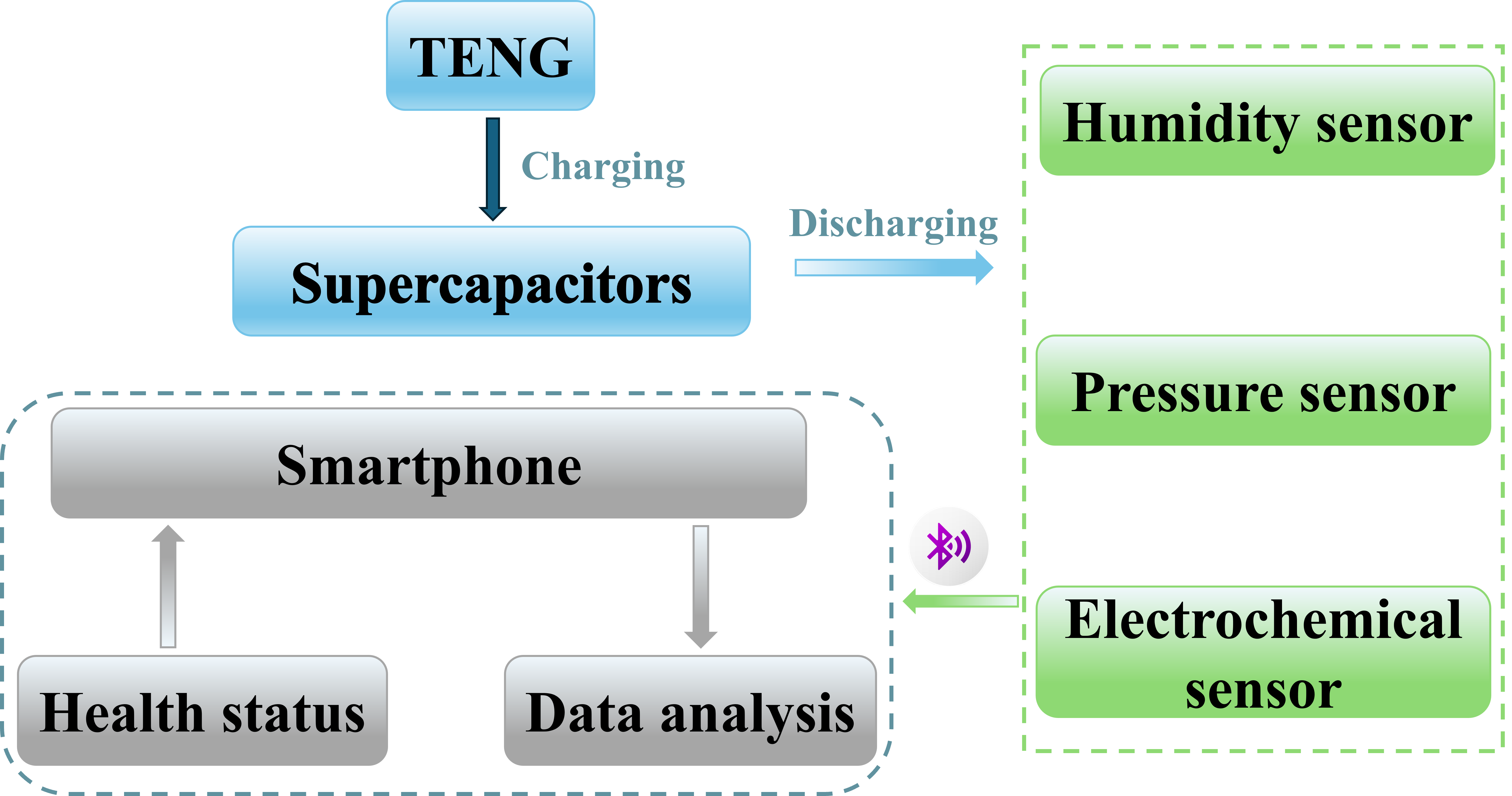
**

**Figure S35.** A working schematic diagram of smart insole


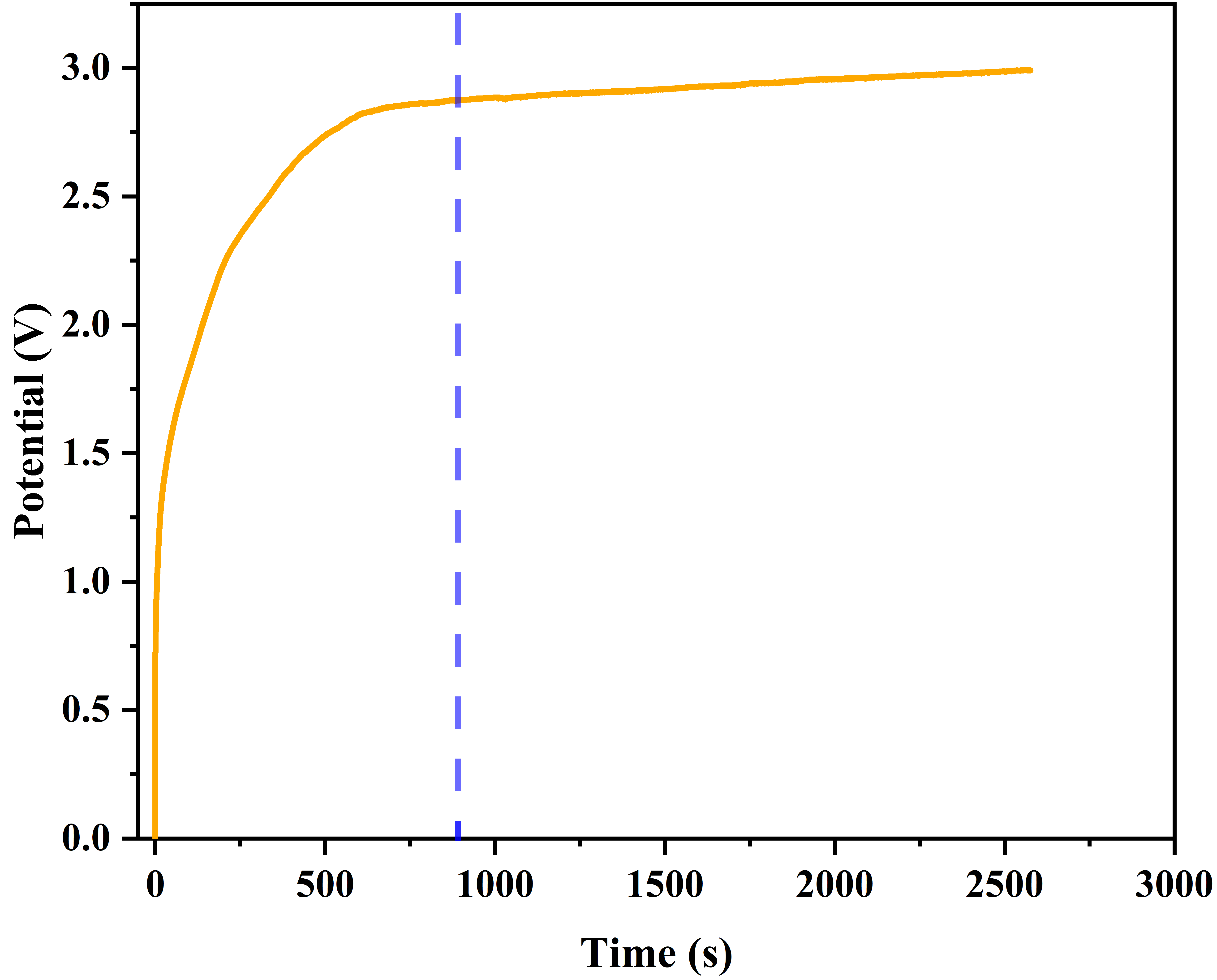


**Figure S36**. TENG charged the supercapacitor for 2,600 seconds.


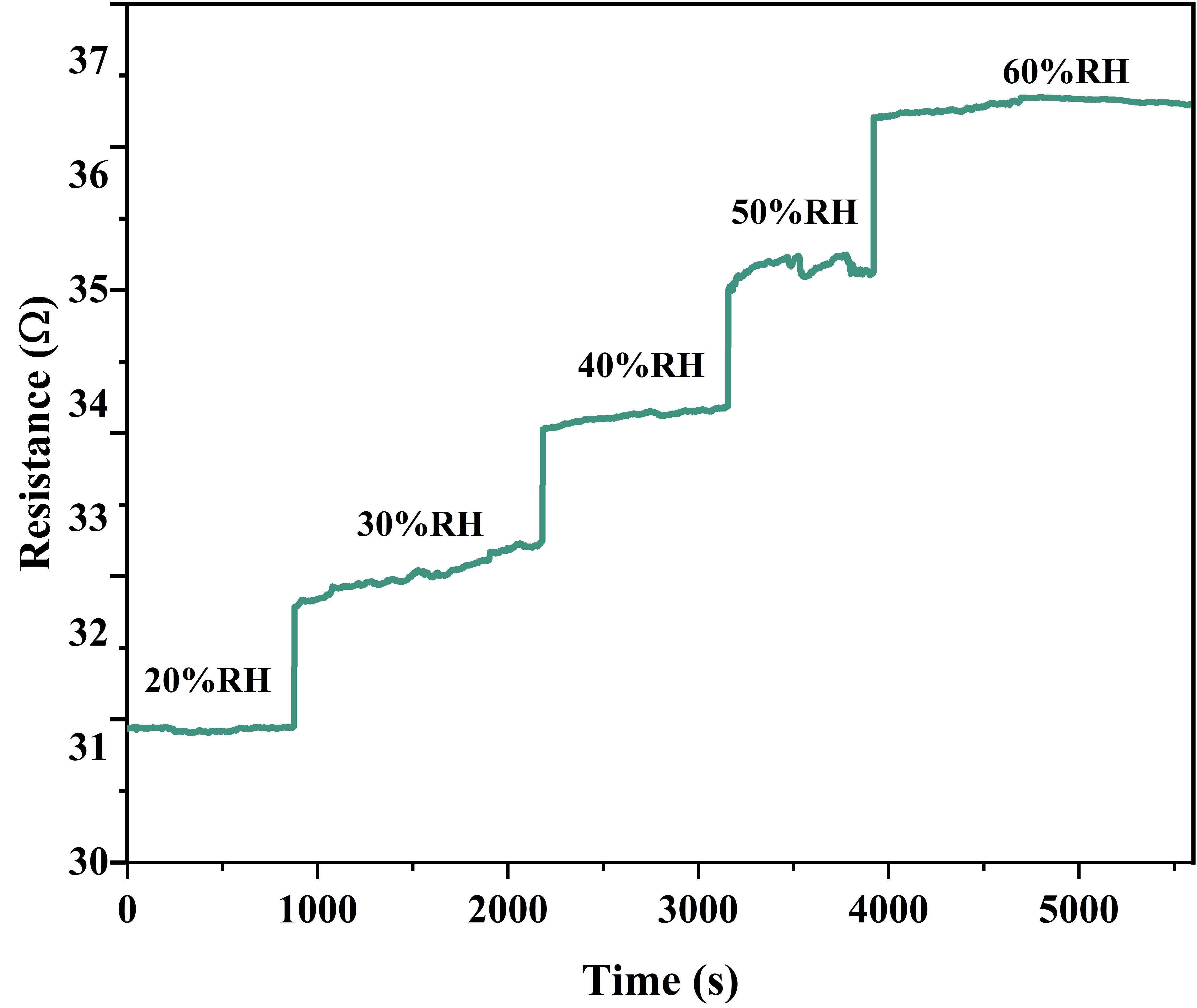


**Figure S37.** The test data after the self-powered module was connected to the humidity sensor.


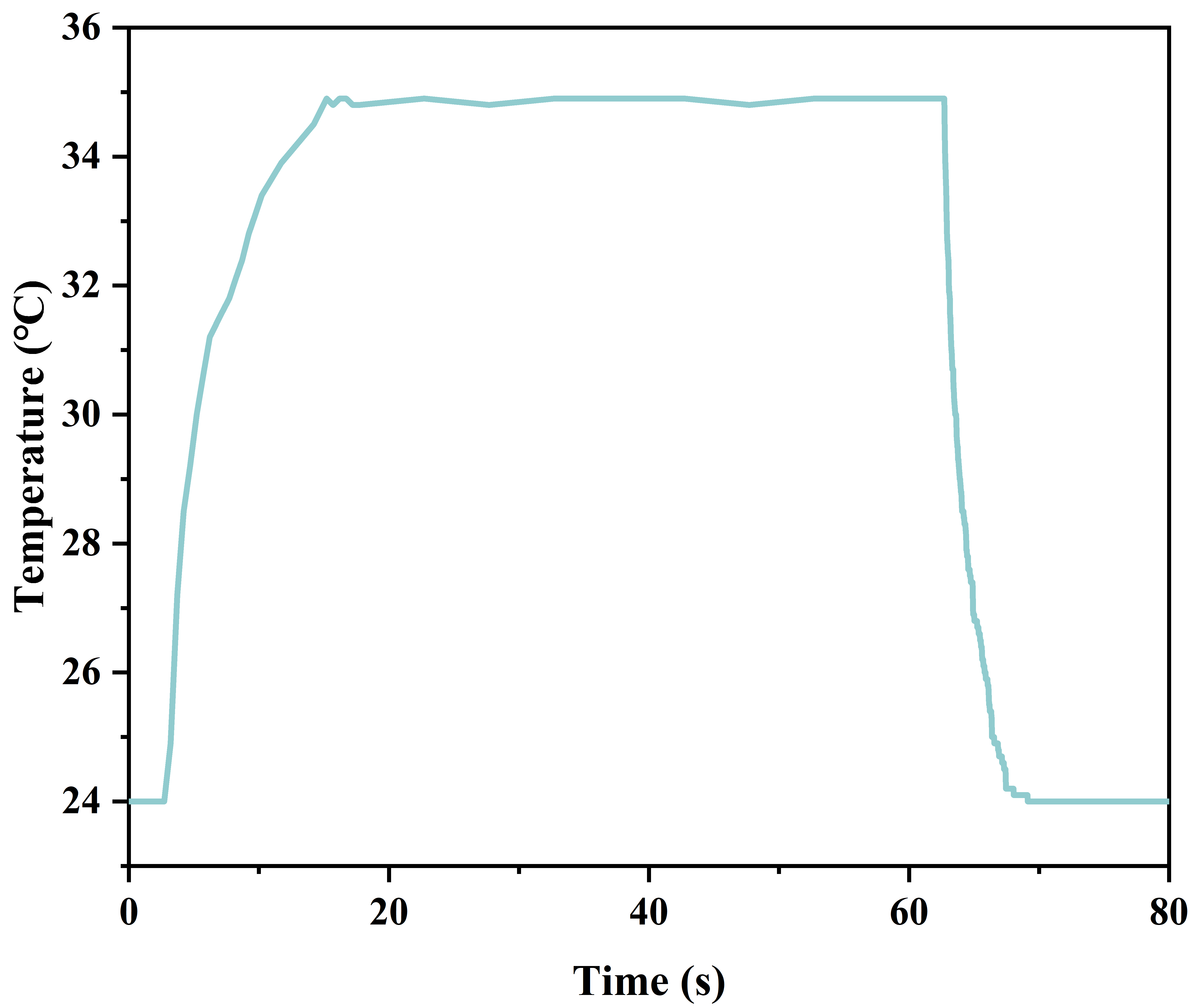


**Figure S38.** The test data after the self-powered module was connected to the Joule heater.


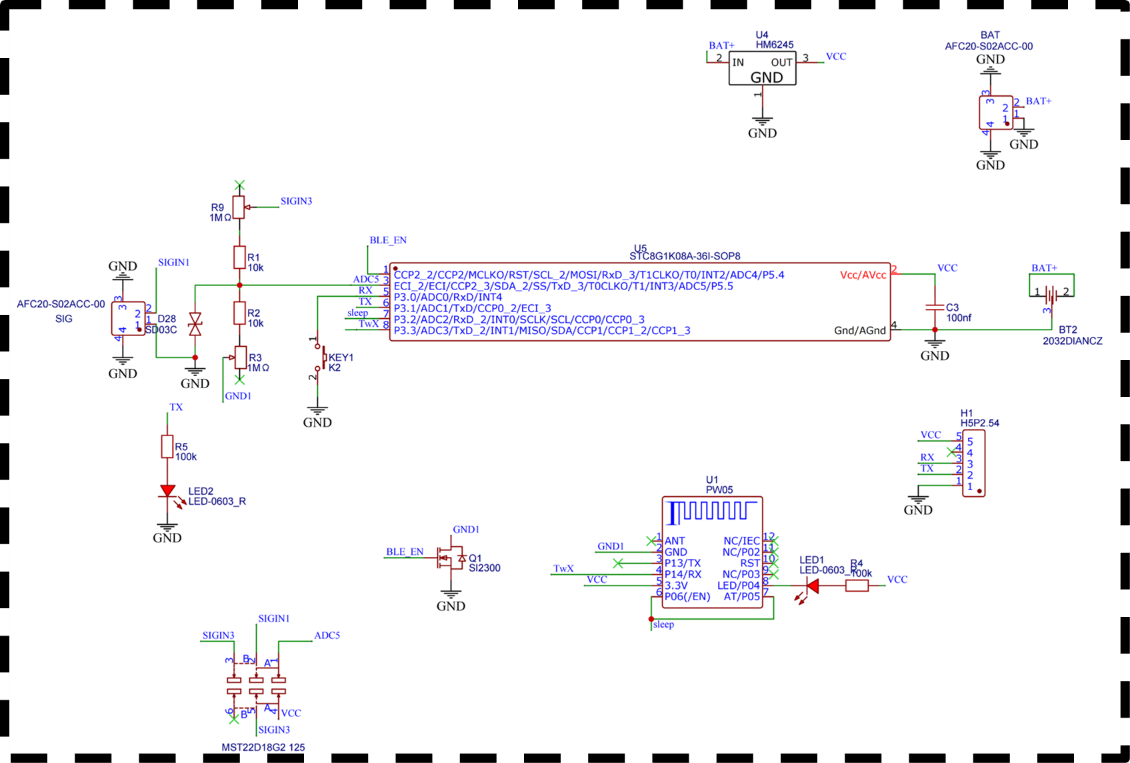


**Figure S39.** Schematic diagram of the working design of the signal conversion device.

**Table S1.** Inventory for 1 LIG@MXene based smart insole.

| *Material/Energy input* | *Functional use* | *Number* | *Units* |
| --- | --- | --- | --- |
| Lignocellulose film | *Sensor material* | *150*2=300* | *cm^2^* |
| Nitrogen-phosphorus flame retardant | *Prevent combustion* | *50* | *mL* |
| MXene (Ti₃C₂Tₓ) | *Sensor material* | *7.5* | *g* |
| Deionized water | *Sensor material* | *142.5+20=162.5* | *mL* |
| DMSO | *Sensor material* | *20* | *mL* |
| *Electricity, low voltage* | *Ultrasonic oscillation* | *0.3* | *kWh* |
| *Electricity, low voltage* | *Centrifugation* | *0.004* | *kWh* |
| *Electricity, low voltage* | *Freeze drying* | *10.4* | *kWh* |
| *Electricity, low voltage* | *Freeze* | *0.7* | *kWh* |
| *Electricity, low voltage* | *Drying* | *2.5* | *kWh* |
| *Electricity, low voltage* | *Spin coating* | *0.002* | *kWh* |
| *Electricity, low voltage* | *Laser etching* | *0.252* | *kWh* |
| *Conductive silver paint* | *Sensor material* | *0.010* | *kg* |
| *PTFE film* | *Sensor material* | *150* | *cm^2^* |
| *Nylon film* | *Sensor material* | *150*2=300* | *cm^2^* |
| *Acrylic film* | *Sensor material* | *150* | *cm^2^* |
| *Conductive copper wire* | *Sensor material* | *20* | *g* |
| *H_2_SO_4_* | *Electrolyte material* | *0.98* | *g* |
| *PVA* | *Electrolyte material* | *1* | *g* |

**Table S2.** Inventory for 1 S-POF smart insole^[1]^.

| *Material/Energy input* | *Functional use* | *Number* | *Units* |
| --- | --- | --- | --- |
| *PMMA optical fiber* | *Sensor core material* | *3.0* | *m* |
| *Solaris silicone rubber* | *Elastomer sensing layer* | *0.3* | *g* |
| *SILASTIC Laboratory Tubing* | *Elastomer encapsulation* | *0.03* | *m* |
| *Black photosensitive resin* | *Connector housing* | *~ 0.48* | *kg* |
| *PLA (Polylactic acid)* | *Fiber fixing module* | *~0.12* | *kg* |
| *Electricity, low voltage* | *3D printing (connector)* | *~1.85* | *kWh* |
| *Electricity, low voltage* | *3D printing (fixing module)* | *~0.26* | *kWh* |
| *Electricity, low voltage* | *Drying* | *~3.0* | *kWh* |
| *Electricity, low voltage* | *Stir* | *~1.5* | *kWh* |
| *Electricity, low voltage* | *Heating* | *~6.0* | *kWh* |

**Table S3.** Inventory for 1 FRdL-insole smart insole^[2]^.

| *Material/Energy input* | *Functional use* | *Number* | *Units* |
| --- | --- | --- | --- |
| PDMS | *Sensor material* | *~150* | *cm^2^* |
| Silicone rubber | *Sensor material* | *~150* | *cm^2^* |
| CNF | *Sensor material* | *~300* | *cm^2^* |
| AgF | *Sensor material* | *~0.5* | *kg* |
| Insulated copper wire | *Sensor material* | *12.8* | *m* |
| Conductive silver paint | *Sensor material* | *~0.12* | *kg* |
| Deionized water | *Ultrasonication* | *~500* | *g* |
| *Electricity, low voltage* | *Drying* | *~0.57* | *kWh* |
| *Electricity, low voltage* | *High-temperature curing* | *~4.61* | *kWh* |

**Table S4.** The performance of the TENG based on LIG@MXene was compared with other reported TENG.

| Materials | Open-circuit voltage (V∙cm^-2^) | Peak current (μA) | Output power (mW∙m^-2^) | Reference |
| --- | --- | --- | --- | --- |
| LIG@MXene | 35 (5 N) | 12.5 (5 N) | 97.1 (5 N) | This work |
| PDMS/MXene | 18.8 (5 N) | 10~11 (5 N） | ~24.7 (5 N) | ^[3]^ |
| LIG | ~ 34.4 (5 N) | / | ~ 5.0 (5 N) | ^[4]^ |
| TPU | < 13 (> 5 N) | / | 51.3 (>5 N) | ^[5]^ |
| LIG | ~26 (10 N) | ~5 (10 N) | 2.25 (50 N) | ^[6]^ |
| CNC/Graphene | <12 | 0.9 | 11.0 | ^[7]^ |

**Table S5.** The performance of the supercapacitor based on LIG@MXene was compared with other reported supercapacitor.

| Materials | Supercapacitor (mF∙cm^-2^) | Current density | Reference |
| --- | --- | --- | --- |
| LIG@MXene | 71.4 | 0.1 mA∙cm^-2^ | This work |
| LIG | 11.2 | 0.1 mA∙cm^-2^ | ^[8]^ |
| MSC-FB | 49.81 | 0.09 mA∙cm^-2^ | ^[9]^ |
| LIG | 15.1 | 0.1 mA∙cm^-2^ | ^[10]^ |
| Mo_3_C_2_/LIG | 23.5 | 0.5 mA∙cm^-2^ | ^[11]^ |
| LIG | 29.94 | 0.15 mA∙cm^-2^ | ^[12]^ |

**Table S6.** The performance of the single-layer piezoresistive pressure sensor based on LIMG was compared with other reported sensors.

| Modified electrode | Manufacturing method | LOD（μM） | Reference |
| --- | --- | --- | --- |
| LIG@MXene | Laser induction | 9.6 | This work |
| Graphene/Polydopamine/Carbon nanotubes | Hydrothermal | 15 | ^[13]^ |
| LIG | Laser induction | 11.43 | ^[14]^ |
| rGO | Hydrothermal | 27 | ^[15]^ |
| LIG/Au/Pd/MXene | Laser induction/self-assembly | 1.47 | ^[16]^ |
| LIG | Laser induction/self-assembly | 15.6 | ^[17]^ |

**Reference**

[1] K. Xiang, M. Liu, J. Chen, Y. Bao, Z. Wang, K. Xiao, C. Teng, N. Ushakov, S. Kumar, X. Li, R. Min, *ACS Applied Materials & Interfaces* **2024**, 16, 32662.

[2] Y. Gao, B. Xu, M. Qiu, Z. Li, T. Ahmed, Y. Yang, X. Guan, H. Fu, *Advanced Functional Materials* **2025**, 35, 2416577.

[3] C. Jiang, X. Li, Y. Yao, L. Lan, Y. Shao, F. Zhao, Y. Ying, J. Ping, *Nano Energy* **2019**, 66, 104121.

[4] W. Yang, M. Han, F. Liu, D. Wang, Y. Gao, G. Wang, X. Ding, S. Luo, *Advanced Science* **2024**, 11, 2310017.

[5] G. B. Pradhan, T. Bhatta, K. Shrestha, S. Sharma, J. Y. Park, *Sensors and Actuators A: Physical* **2024**, 379, 115985.

[6] P. Zhao, G. Bhattacharya, S. J. Fishlock, J. G. M. Guy, A. Kumar, C. Tsonos, Z. Yu, S. Raj, J. A. McLaughlin, J. Luo, N. Soin, *Nano Energy* **2020**, 75, 104958.

[7] M. S. Alghamdi, J. J. Morgan, K. Walsh, D. W. Shin, R. Nigmatullin, Z. Saadi, J. Routledge, A. I. S. Neves, S. Russo, S. J. Eichhorn, M. F. Craciun, *Nano Energy* **2025**, 138, 110816.

[8] A. Imbrogno, J. Islam, C. Santillo, R. Castaldo, L. Sygellou, C. Larrigy, R. Murray, E. Vaughan, M. K. Hoque, A. J. Quinn, D. Iacopino, *ACS Applied Electronic Materials* **2022**, 4, 1541.

[9] G. Yuan, T. Wan, A. BaQais, Y. Mu, D. Cui, M. A. Amin, X. Li, B. B. Xu, X. Zhu, H. Algadi, H. Li, P. Wasnik, N. Lu, Z. Guo, H. Wei, B. Cheng, *Carbon* **2023**, 212, 118101.

[10] A. Ghosh, S. Kaur, G. Verma, C. Dolle, R. Azmi, S. Heissler, Y. M. Eggeler, K. Mondal, D. Mager, A. Gupta, J. G. Korvink, D.-Y. Wang, A. Sharma, M. Islam, *ACS Applied Materials & Interfaces* **2024**, 16, 40313.

[11] C. Yang, X. Zhou, Y. Tian, J. Zhu, M. Xiao, S. Xie, Y. Zhu, Y. Huang, *Chemical Engineering Journal* **2024**, 499, 156519.

[12] L. Du, B. Quan, Z. Xu, X. Sun, Y. Luo, J. Travas-Sejdic, B. Zhu, *Carbon* **2025**, 238, 120225.

[13] T. Song, X. Guo, X. Li, S. Zhang, *Journal of Electroanalytical Chemistry* **2016**, 781, 251.

[14] L. Yang, H. Wang, A. M. Abdullah, C. Meng, X. Chen, A. Feng, H. Cheng, *ACS Applied Materials & Interfaces* **2023**, 15, 34332.

[15] F. Besbes, Z. Hsine, R. Mlika, *Carbon Letters* **2023**, 33, 2109.

[16] Y. Wang, P. Zhao, B. Gao, M. Yuan, J. Yu, Z. Wang, X. Chen, *Microchemical Journal* **2023**, 185, 108177.

[17] A. Soleimani, F. Amirghasemi, A. Al-Shami, S. Khazaee Nejad, A. Tsung, Y. Wang, S. Lara Galindo, D. Parvin, A. Olson, A. Avishai, M. P. S. Mousavi, *Biosensors and Bioelectronics* **2024**, 259, 116321.
